# Sphingolipid signatures at the extremes of energy balance: a comparative study of obesity and anorexia nervosa in women

**DOI:** 10.3389/fendo.2026.1890712

**Published:** 2026-10-08

**Authors:** Antonello E. Rigamonti, Camillo Morano, Michele Dei Cas, Aldijana Sadikovic, Adele Bondesan, Diana Caroli, Francesca Frigerio, Laura Abbruzzese, Sandra Savino, Silvano G. Cella, Rita Paroni, Alessandro Sartorio

**Affiliations:** 1 Department of Clinical Sciences and Community Health, Dipartimento di Eccellenza 2023–2027, University of Milan, Milan, Italy; 2 Department of Health Sciences, University of Milan, Milan, Italy; 3 Experimental Laboratory for Auxo-Endocrinological Research, Istituto Auxologico Italiano, IRCCS (Istituto di Ricovero e Cura a Carattere Scientifico), Piancavallo, Verbania, Italy; 4 Division of Auxology, Istituto Auxologico Italiano, Istituto di Ricovero e Cura a Carattere Scientifico (IRCCS), Piancavallo, Verbania, Italy; 5 Division of Eating and Nutrition Disorders, Istituto Auxologico Italiano, Istituto di Ricovero e Cura a Carattere Scientifico (IRCCS), Piancavallo, Verbania, Italy

**Keywords:** anorexia nervosa, cardiometabolic variables, normal-weight, obesity, sphingolipidomics

## Abstract

**Background:**

Anorexia nervosa (AN) and obesity (OB) represent opposite extremes of energy balance and are both associated with profound metabolic alterations. Increasing evidence suggests that sphingolipids play a central role in cardiometabolic regulation, inflammation, and insulin signaling; however, clinical sphingolipidomic studies have largely investigated AN and OB separately. Direct comparative studies using a harmonized analytical framework are currently lacking.

**Objectives:**

The primary objective of this study was to determine whether women with AN or OB exhibit distinct plasma sphingolipidomic signatures compared with a normal-weight healthy (NWH) group and with each other. Secondary objectives included identifying multivariate sphingolipid patterns associated with nutritional phenotype and evaluating associations between plasma sphingolipids and cardiometabolic parameters.

**Methods:**

In this cross-sectional study, 114 women were enrolled (28 with AN, 37 with OB, and 49 NWH controls). Plasma sphingolipidomics was assessed using targeted liquid chromatography–tandem mass spectrometry (LC–MS/MS). Clinical, anthropometric, biochemical, inflammatory, and hemodynamic variables were collected in all participants. Group comparisons were performed using non-parametric statistics with Benjamini–Hochberg false discovery rate (FDR) correction. Multivariate analyses included principal component analysis (PCA), hierarchical clustering analysis (HCA), partial least squares discriminant analysis (PLS-DA), and variable importance in projection (VIP) analysis. Age-adjusted regression analyses were performed to investigate associations between sphingolipid variables and cardiometabolic predictors.

**Results:**

Clinical and metabolic variables differed among the AN, NWH, and OB groups. After FDR correction, 38 of 45 sphingolipid variables differed significantly among groups, involving ceramides, dihydroceramides, sphingomyelins, glycosphingolipids, sphingoid-base-related metabolites, and ceramide ratio indices. AN was characterized by increased levels of several sphingomyelins, lactosylceramides, GM3 species, S1P, and DhS1P. In contrast, OB showed enrichment of selected dihydroceramides and ceramide ratio indices together with lower levels of several very-long-chain glycosphingolipids. The direct OB-versus-AN comparison yielded the largest number of FDR-significant variables among the three pairwise comparisons. PCA, hierarchical clustering, and PLS-DA consistently demonstrated structured multivariate separation among groups. The supervised PLS-DA model achieved an overall accuracy of 0.851 and identified DHCer 24:1, DHCer 18, HexCer 22, Cer 24:1, SM 24:1, and DHCer 18:1 among the variables with the highest VIP scores. Age-adjusted analyses revealed significant associations between sphingolipid variables and BMI, HOMA-IR, blood pressure, CRP, triglycerides, and HDL-cholesterol.

**Conclusions:**

Women with AN and OB exhibit distinct but partially overlapping plasma sphingolipidomic signatures, characterized by coordinated remodeling across multiple sphingolipid pathways. AN was predominantly associated with sphingomyelin- and glycosphingolipid-enriched patterns, whereas OB was characterized by dihydroceramide- and ceramide-ratio-associated remodeling. These findings support the concept that severe undernutrition and obesity represent biologically distinct states of sphingolipid dysregulation and suggest that plasma sphingolipid signatures may serve as candidate biomarkers of metabolic remodeling and cardiometabolic risk across the extremes of energy balance.

## 1 Introduction

Anorexia nervosa (AN) and obesity (OB) represent opposite extremes of energy balance and are increasingly recognized as systemic metabolic disorders rather than conditions solely characterized by altered body weight. While AN has emerged as a metabo-psychiatric disorder characterized by profound energy deprivation and paradoxical metabolic abnormalities, including persistent dyslipidemia and altered lipid handling (1–7), OB is characterized by energy excess, dysfunctional adiposity, insulin resistance, chronic low-grade inflammation, and extensive disturbances in lipid metabolism (8–11).

Among lipid classes, sphingolipids—including ceramides, dihydroceramides, sphingomyelins, glycosphingolipids, and sphingosine-1-phosphate—have emerged as key bioactive molecules involved in membrane organization, mitochondrial function, insulin signaling, inflammatory regulation, and cardiovascular risk (8, 10–12). In humans, circulating sphingolipid species have been associated with visceral adiposity, insulin resistance, and cardiometabolic dysfunction, suggesting that sphingolipid metabolism represents an important interface linking nutritional status to systemic metabolic health (8–12).

The current evidence, however, has evolved asymmetrically across the two ends of the body-weight spectrum. In OB, numerous clinical and translational studies have implicated ceramides and related sphingolipids in insulin resistance, adipose tissue dysfunction, ectopic lipid accumulation, and cardiometabolic disease progression (8–12). Conversely, recent metabolomic, lipidomic, and sphingolipidomic studies have demonstrated that AN is characterized by complex metabolic remodeling rather than simple lipid depletion, including paradoxically elevated lipid classes, persistent lipid abnormalities after short-term weight restoration, and alterations in ceramide-related pathways (3–7). Collectively, these findings suggest that both severe caloric restriction and excessive adiposity profoundly affect sphingolipid homeostasis, although potentially through different biological mechanisms (3–12).

Despite these advances, an important translational gap remains. Clinical sphingolipidomic investigations have largely examined AN and OB separately, typically comparing each disorder with normal-weight controls. Consequently, direct head-to-head comparisons performed within the same recruitment framework, analytical platform, and pre-analytical conditions are currently lacking (5–11). Such comparisons are particularly important because cross-study differences in sex, nutritional status, treatment phase, analytical methodology, and clinical phenotyping limit the biological interpretation of indirect comparisons (3–11).

Therefore, the present study directly compared women with AN or OB, and normal-weight healthy (NWH) controls using the same analytical and clinical framework. The primary objective was to determine whether AN and OB exhibit distinct plasma sphingolipidomic signatures compared with NWH women and with each other. Secondary objectives were to identify multivariate sphingolipid patterns associated with nutritional phenotype, evaluate whether AN and OB display opposite, overlapping, or partially shared sphingolipidomic remodeling, and investigate the relationships between plasma sphingolipids and clinically relevant anthropometric, inflammatory, hemodynamic, and glucometabolic variables. Overall, the study was designed to explore the potential value of plasma sphingolipid signatures in AN and OB as candidate biomarkers of metabolic remodeling and cardiometabolic risk for both conditions. Restricting the analysis to women was intended to reduce biological heterogeneity arising from well-recognized biological sex differences in adiposity, lipoprotein metabolism, inflammatory profile, and circulating sphingolipid concentrations, thereby improving the internal validity of the present analyses. This methodological choice should not be interpreted as implying that sphingolipid alterations in men are less relevant; rather, future studies including male participants are warranted to determine the generalizability of the present findings.

## 2 Materials and methods

### 2.1 General information

A cross-sectional design was employed in the present study to determine plasma sphingolipidomics in AN and OB (versus NWH), and to assess associations between plasma levels of individual sphingolipids (or sphingolipid ratios) and some relevant cardiometabolic parameters (see below).

### 2.2 Subjects

#### 2.2.1 AN group

Women with AN were consecutively recruited at the Division of Eating and Nutrition Disorders of Istituto Auxologico Italiano, IRCCS, Piancavallo-Verbania, a tertiary referral centre in Northern Italy specialized in the multidisciplinary management of eating disorders and severe obesity, including metabolic, nutritional, and psychological rehabilitation. Among the enrolled patients, 25 were amenorrheic, 1 had preserved spontaneous menstrual cycles, and 2 were receiving oral contraceptive therapy at the time of study participation.

Eligibility criteria included female sex, age between 18 and 60 years, and a diagnosis of AN established according to the Diagnostic and Statistical Manual of Mental Disorders, Fifth Edition (DSM-5) criteria through the Structured Clinical Interview for DSM-5 Disorders, Clinical Version (SCID-5-CV) (13).

Exclusion criteria included the presence of major comorbid psychiatric disorders according to DSM-5 criteria, as well as any concomitant medical condition that the investigators believed would interfere with study participation or with the interpretation of metabolic and clinical assessments.

All enrolled patients were admitted to the centre for the first time, although some had previously undergone outpatient clinical evaluations. Following admission, patients underwent a structured multidisciplinary inpatient rehabilitation program in a residential setting lasting between three and eight weeks, as previously described (14). The rehabilitation intervention integrated medical, nutritional, psychological, and rehabilitative components within a comprehensive multidisciplinary framework.

Psychological and psychotherapeutic interventions included both individual and group sessions aimed at improving emotional regulation, psychopathological symptom burden, and body-related experience. Body image disturbances were specifically addressed through body image (BI) therapy (15). In parallel, low-intensity adapted physical activity programs were implemented to channel patients’ drive for movement into supervised, metabolically appropriate exercise interventions that promote healthy lifestyle behaviors (16–18).

Psychological functioning, including depressive symptoms, anxiety, and post-traumatic symptomatology, was addressed through both individual and group psychotherapy sessions (19). Nutritional rehabilitation consisted of individualized dietary counseling and group-based nutritional education delivered by specialized nutrition professionals, with the aim of promoting appropriate eating behaviors, awareness of hunger and satiety cues, and knowledge of nutrient composition and metabolic requirements (20). During hospitalization, all meals were supervised by trained dietitians and nursing staff within an assisted-meal framework designed to support adherence to the nutritional rehabilitation program.

#### 2.2.2 OB group

Women with OB (body mass index, BMI > 35 kg/m2), who were hospitalized at the Division of Metabolic Diseases, Istituto Auxologico Italiano, Piancavallo-Verbania, Italy, for a 3-week multidisciplinary integrated body weight reduction program (BWRP), were recruited for the current study. All females were eumenorrheic; the study was conducted during the follicular phase of their menstrual cycle.

After verification of the exclusion criteria, particularly the presence of any disease other than OB (with or without metabolic syndrome), clinical, biochemical, and anthropometric data were collected from each participant before starting the BWRP, including body composition assessment by bioimpedance analysis (see below for details).

#### 2.2.3 NWH group

NWH female controls were recruited from the hospital’s medical, research, and administrative staff, as well as among friends and colleagues. The control group excluded persons with relevant disorders, such as psychiatric disorders. Convenience sampling was used because it is practical for accessing readily available populations. Although this approach inherently limits generalizability, participants were recruited from the same geographical area and within the same institutional environment whenever possible. Educational attainment and socioeconomic status were not formally assessed and were therefore not considered matching variables. All females were eumenorrheic; the study was conducted during the follicular phase of their menstrual cycle.

### 2.3 Ethical aspects

The study was approved by the Ethical Committee (EC) of the Istituto Auxologico Italiano, IRCCS, Milan, Italy (EC code: 2021_02_23_11; date of approval: February 23, 2021; research project: 01C126; acronym: SFINGOTRANSADIP) and by the Ethical Committee no. 5, Lombardy Region (registration number: 209/24; date of approval: April 23, 2024; research order code: 01C417, acronym: SFINGOANNER);

All procedures were conducted in accordance with the Declaration of Helsinki of 1975 and its subsequent amendments, or with comparable ethical standards.

The protocol was explained to the subjects, who provided written informed consent before enrollment.

### 2.4 Clinical assessment

#### 2.4.1 Anthropometric measurements

A scale with a stadiometer was used to measure height and weight (Wunder Sa.Bi., WU150, Trezzo sull’Adda, Italy). Waist circumference (WC) was measured with a flexible tape in a standing position, halfway between the inferior margin of the ribs and the superior border of the iliac crest. Body composition was measured by bioimpedance analysis (Human-IM Scan, DS-Medigroup, Milan, Italy) after 20 min of supine rest. BMI, fat mass (FM), and fat-free mass (FFM) were determined in all subjects.

#### 2.4.2 REE measurement

Resting energy expenditure (REE) was measured between 8:00 and 10:00 a.m. in thermoneutral conditions (room temperature: 22–25 °C) using an open-circuit, indirect, computerized calorimeter equipped with a canopy (Vmax 29, Sensor Medics, Yorba Linda, CA, USA). The calorimeter underwent periodic quality control tests to ensure measurement reliability. The gas analyzers were calibrated before each test using a reference gas mixture of 15% O2 and 5% CO2. The participants had fasted for at least 8 h, had not smoked for at least 1 h, and waited 30 min in a sitting position before undergoing REE measurement. REE was assessed in the supine position for at least 30 min, including an acclimation period of 10 min. Data from the acclimation period were discarded. The steady state was defined as at least 5 min with less than 5% variation in the respiratory quotient and ventilation (21). After the steady state was reached, O2 consumption and CO2 production were recorded at 1 min intervals for at least 20 min and averaged over the entire measurement period. REE was calculated from O2 consumption and CO2 production using Weir’s equation (22).

#### 2.4.3 Blood pressure

Blood pressure was measured on the right arm using a sphygmomanometer with an appropriate cuff size, with the subject seated and relaxed. The procedure was repeated three times at 10-min intervals; the means of the three values for systolic (SBP) and diastolic (DBP) blood pressure were recorded.

### 2.5 Laboratory procedures

#### 2.5.1 Metabolic variables

Fasting blood samples (~10 mL) were collected at approximately 8:00 AM after an overnight fast (~12 h).

Serum levels of total cholesterol (T-C), HDL cholesterol (HDL-C), LDL cholesterol (LDL-C), triglycerides (TG), glucose, insulin, C-reactive protein (CRP), and glycated hemoglobin (HbA1c) were measured using standardized laboratory methods.

Lipid parameters (T-C, HDL-C, LDL-C, and TG) and glucose were measured using enzymatic colorimetric assays (Roche Diagnostics, Monza, Italy). Insulin was measured using a chemiluminescent immunometric assay (Elecsys Insulin, Roche Diagnostics), and (high sensivity) CRP by immunoturbidimetric assay (<1 mg/L = low cardiovascular risk; 1–3 mg/L = moderate cardiovascular risk; >3 mg/L = high cardiovascular risk). HbA1c was quantified by high-performance liquid chromatography (HPLC) based on cation-exchange principles (Bio-Rad D-10 system), with calibration traceable to International Federation of Clinical Chemistry (IFCC) standards. Results were expressed as a percentage of total hemoglobin.

Assay sensitivities were within standard analytical ranges (≤9 mg/dL for lipids and triglycerides, 2 mg/dL for glucose, 0.2 µIU/mL for insulin, and 0.03 mg/dL for CRP). Analytical precision was confirmed by intra- and inter-assay coefficients of variation below 5% for all parameters.

Insulin resistance was estimated using the homeostatic model assessment (HOMA-IR), calculated as: insulin (µIU/mL) × glucose (mmol/L)/22.5 (23).

#### 2.5.2 Lipid extraction and sphingolipid content quantification

Sphingolipid extraction and targeted LC–MS/MS analysis were performed as previously described (24, 25). Sphingolipids were assayed in 25 µL of plasma collected as described above. This material was diluted to a final volume of 100 µL with water and, after the addition of 850 µL of a methanol/chloroform mixture (2:1 v/v), the samples were thermos-shaken (1000 rpm) for 1 h at 38 °C. Then, to enhance recovery, alkaline methanolysis was performed by thermos-shaking (1000 rpm) at 38 °C for 2 h with 75 µL of 1 M potassium hydroxide in methanol. After neutralization with 4 µL of pure acetic acid, samples were centrifuged (15 min at 20,000 × g) and the organic phase evaporated. The residues were dissolved in 100 µL of methanol + 0.5 mg/mL of butylated hydroxytoluene (BHT), centrifuged for 10 min at 20,000 ×g, and transferred to a glass vial. Finally, samples were analyzed by LC Dionex 3000 UltiMate (Thermo Fisher Scientific) coupled to a tandem mass spectrometer AB Sciex 3200 QTRAP (AB Sciex, Marlborough, MA, USA). The separation was achieved by reversed-phase chromatography, either using BEH C8 1.7 μm, 100 × 2.1 mm (Waters, Milford, MA, USA) (for ceramides, dihydroceramides, sphingomyelins and glycosylated sphingolipids) or Cortecs C18 1.6 μm, 100 × 2.1 mm (Waters, Milford, MA, USA) (for sphingoid bases) by mixing eluent A (0.2% formic acid, 2 mM ammonium formate water solution) and eluent B (methanol, 0.2% formic acid, 1 mM ammonium formate). Quantitative analysis was performed by interpolating each analyte peak area/internal standard area using a calibration curve for each sphingolipid.

#### 2.5.3 Quality control and analytical validation of lipidomic measurements

To ensure the robustness, reproducibility, and analytical reliability of the sphingolipidomic data, a comprehensive set of quality control (QC) procedures was implemented throughout the experimental workflow, including sample preparation, LC–MS/MS acquisition, and data processing.

First, all samples were processed using a standardized and fully harmonized protocol for plasma collection from blood sampling and lipid extraction, minimizing pre-analytical variability. Sample handling times were tightly controlled, and all specimens were processed within 2 h of blood collection to reduce degradation and ex vivo metabolic alterations.

Quantitative lipidomic analysis was performed using internal standard–based calibration. For each sphingolipid class, appropriate internal standards were added before extraction to correct for extraction efficiency, ionization variability, and instrument response. Calibration curves were generated for each analyte, and only signals within the linear dynamic range were considered for quantification.

To monitor analytical performance, pooled quality control samples were prepared by combining aliquots from multiple plasma extracts and were periodically injected throughout the LC–MS/MS sequence. These QC samples were used to assess instrument stability, retention time consistency, and signal reproducibility across runs. The coefficient of variation (CV) for the main quantified sphingolipid species in QC samples was monitored and confirmed to be within acceptable analytical ranges.

Batch effects were minimized by analyzing samples in a randomized order across experimental runs. In addition, instrument performance was regularly checked using standard tuning procedures and evaluation of key parameters such as mass accuracy, sensitivity, and chromatographic resolution.

Although technical replicates at the extraction level were limited by sample availability, the combination of internal standard normalization, QC sample monitoring, and controlled analytical conditions ensured the reliability and comparability of the lipidomic measurements across all study groups.

### 2.6 Data analysis

#### 2.6.1 Statistical analysis and software

Continuous variables were tested for normality using the Shapiro–Wilk test. As most sphingolipid concentrations were not normally distributed, data are presented as medians and interquartile ranges (IQR), and non-parametric methods were used for group comparisons. Differences among groups (NWH subjects and patients with AN or OB) were assessed using the Kruskal–Wallis test, followed by Dunn’s *post hoc* test for pairwise comparisons. When appropriate, to account for multiple testing across sphingolipid species, p-values were adjusted using the Benjamini–Hochberg false discovery rate (FDR) procedure.

Categorical variables are presented as counts or percentages. Differences across groups were assessed using the chi-square test.

Principal component analysis (PCA) was performed on the complete set of sphingolipid variables to explore the dataset’s global structure and to visualize sample clustering. Before PCA, data were log-transformed to reduce skewness and autoscaled (mean-centered and divided by the standard deviation) to ensure comparability across variables with different dynamic ranges. The proportion of explained variance by each principal component was reported.

Hierarchical clustering analysis was conducted using Euclidean distance and Ward’s linkage method on the subset of sphingolipids that were statistically significant after FDR correction in univariate group comparisons. Data were log-transformed and standardized before clustering. Heatmaps were generated to visualize patterns of relative abundance across samples, with rows representing subjects and columns representing lipid species.

Partial least squares discriminant analysis (PLS-DA) was performed using the subset of sphingolipids identified as significant in univariate analyses to evaluate multivariate discrimination among groups. Model performance was assessed using stratified five-fold cross-validation, and variable importance in projection (VIP) scores were calculated to identify the most influential variables contributing to group separation.

Univariate linear regression analyses were conducted to investigate associations between sphingolipid species and cardiometabolic parameters, including BMI, WC, SBP, DBP, insulin resistance (HOMA-IR), HDL-C, TG, and CRP. Each sphingolipid was modeled as a dependent variable in separate ordinary least squares (OLS) regression models. Regression coefficients (β), standard errors (SE), 95% confidence intervals (CI), standardized coefficients (Std. β), and adjusted R2 values were reported. To account for multiple testing in regression analyses, p-values were adjusted using the Benjamini–Hochberg procedure applied globally (i.e., across all sphingolipids tested for global clinical/biochemical variables), thereby controlling for the number of all comparisons. This approach was chosen to balance type I error control with the correlation structure of lipidomic and clinical/biochemical variables.

A two-sided p-value < 0.05 or FDR-adjusted p-value < 0.05, when appropriate, was considered statistically significant.

All statistical analyses were performed using Python (v. 3.12.9; Python Software Foundation, Wilmington, DE, USA). Data handling and preprocessing were conducted using Pandas (v. 2.2.3) and NumPy (v. 2.1.3). Group comparisons and hierarchical clustering analyses were performed using the SciPy library (v. 1.14.1). Multiple univariate ordinary least squares regression models were computed using Statsmodels (v. 0.14.4). PCA and PLS-based multivariate analyses were performed using Scikit-learn (v. 1.5.2). Volcano plots, heatmaps, boxplots, and additional graphical visualizations were generated using Matplotlib (v. 3.9.2). All software listed above is available as open-source community software.

To facilitate the interpretation of the overall statistical strategy, the sequential rationale and complementary role of each method (Volcano plot, PCA, HCA, heatmap, PLS-DA, and VIP analysis) are summarized in **Supplementary Figure 1**. This schematic workflow illustrates the specific statistical question addressed by each analytical approach and highlights how the individual methods collectively contribute to the biological interpretation of the sphingolipidomic dataset.

#### 2.6.2 Rationale for variable selection in multivariate analyses

In the present study, both exploratory and targeted statistical strategies were applied to the sphingolipidomic dataset in order to balance comprehensive pattern discovery with model interpretability and robustness.

For global exploratory analyses, including PCA and volcano plot visualization, the full set of quantified sphingolipid variables was retained (n = 42 individual species plus 3 ratio indices; total n = 45 variables). The three ceramide ratio indices were intentionally retained because they represent biologically meaningful composite markers of ceramide metabolism that have been associated with cardiometabolic risk in previous studies. Although these ratios share individual analytes with the corresponding ceramide species, they were considered to provide complementary biological information rather than simple mathematical duplication and they were therefore included in the exploratory multivariate analyses.

This inclusive choice was driven by the hypothesis-free nature of these approaches, which aim to capture the global structure of the lipidomic dataset without prior assumptions or variable selection. By including all measured sphingolipid variables, these analyses preserve the intrinsic covariance structure of the data and enable the identification of dominant axes of variation that reflect coordinated metabolic remodeling.

In contrast, for supervised or structure-enhancing analyses, such as hierarchical clustering and PLS-DA, the variable space was restricted to the subset of sphingolipid variables that were statistically significant in univariate group comparisons after FDR correction (n = 35 or 38 variables). This selection was performed to reduce dimensionality relative to sample size, improve model stability, and limit the inclusion of noisy or non-informative variables that could obscure biologically meaningful patterns. Given the study’s moderate sample size, this approach was considered appropriate to mitigate overfitting and enhance the interpretability of clustering and discrimination results.

Similarly, univariate regression analyses of cardiometabolic variables were conducted using only the subset of previously significant sphingolipids (n = 38 variables). This targeted approach was adopted to focus on lipid species most likely to be biologically relevant and to reduce the multiple testing burden inherent in high-dimensional datasets. To further control the type I error rate, FDR correction was applied globally, as detailed above (Section 2.6.1).

#### 2.6.3 Missing data

##### 2.6.3.1 Extent of missing data

The proportion of missing data was generally low for clinical and anthropometric variables (<5%), while slightly higher levels were observed for selected biochemical and sphingolipid measurements (approximately 5–15%), likely reflecting technical and analytical variability. No variable showed excessive missingness.

##### 2.6.3.2 Missing data handling strategy

Missing values were imputed using the median calculated within each study group (NWH, AN, and OB), rather than a single global median across all participants. Group-specific median imputation was chosen to preserve the biological differences among the study groups while minimizing the influence of missing observations. Given the relatively low proportion of missing data and the non-normal distribution of several variables, this approach was considered more appropriate than global median imputation.

##### 2.6.3.3 Assumptions on missingness

Based on the distribution of missing values across groups and variables, data were assumed to be missing at random (MAR).

##### 2.6.3.4 Sensitivity Considerations

Given the limited proportion of missing data, median imputation is unlikely to have materially affected the results.

### 2.6.4 Handling of measurements below the limit of detection

In addition to missing values, a limited number of lipid species yielded concentrations below the analytical limit of detection (LOD). These observations do not represent missing measurements, as the corresponding samples were successfully analyzed, but rather analyte concentrations that could not be reliably quantified under the analytical conditions employed. According to the analytical data-processing procedure, values below the LOD were conventionally recorded as zero and were retained as such in all statistical analyses. Therefore, no imputation procedure was applied to these observations.

Measurements below the LOD involved DHCer 18:1, GM3 18:1, and DhSph. For DHCer 18:1, concentrations below the LOD were observed in 11 of 49 NWH subjects (22.4%), all 28 AN patients (100%), and none of the 37 OB patients (0%). For GM3 18:1, concentrations below the LOD occurred in 13 of 49 NWH subjects (26.5%), all 28 AN patients (100%), and 10 of 37 OB patients (27.0%). For DhSph, concentrations below the LOD were observed in 11 of 49 NWH subjects (22.4%), all 28 AN patients (100%), and none of the 37 OB patients (0%).

Thus, measurements below the LOD were analytically and statistically distinguished from missing values, which were handled separately using group-specific median imputation, as described in Section 2.6.3.

## 3 Results

### 3.1 Comparisons in demographic, clinical, and biochemical parameters

**Table 1** summarizes the demographic, anthropometric, biochemical, and clinical characteristics of the three study groups. The analyzed cohort included 114 subjects: 49 NWH subjects, 28 patients with AN, and 37 patients with OB.

**Table 1:** Descriptive statistics and group comparisons of demographic, biochemical, and clinical parameters in normal-weight healthy (NWH) subjects and patients with anorexia nervosa (AN) or obesity (OB).

Variable	NWH	AN	OB	p_overall	NWH vs AN	NWH vs OB	AN vs OB	Effect size (ϵ²) [95% CI]
n.	49	28	37	/	/	/	/	/
Age (years)	28.10 (26.00-31.90)	23.70 (19.93-30.05)	29.70 (23.50-36.80)	0.0123	0.0165	1.0000	0.0368	0.061 [0.003-0.173]
Weight (kg)	60.00 (56.00-68.00)	39.35 (36.62-42.10)	114.50 (111.50-122.00)	<0.0001	<0.0001	<0.0001	<0.0001	0.863 [0.812-0.879]
Height (m)	1.67 (1.62-1.70)	1.62 (1.56-1.69)	1.64 (1.60-1.68)	0.0846	0.1380	0.2737	1.0000	0.026 [0.000-0.162]
BMI (kg/m2)	22.09 (19.66-23.73)	15.54 (14.03-16.29)	43.02 (41.32-46.20)	<0.0001	<0.0001	<0.0001	<0.0001	0.868 [0.833-0.883]
FFM (kg)	47.47 (42.16-49.68)	33.88 (32.80-38.00)	54.86 (50.55-61.47)	<0.0001	<0.0001	<0.0001	<0.0001	0.621 [0.511-0.711]
FFM (%)	78.10 (71.00-81.70)	88.70 (84.40-93.00)	47.50 (44.90-51.10)	<0.0001	0.0001	<0.0001	<0.0001	0.798 [0.713-0.846]
FM (kg)	12.93 (10.63-18.24)	4.32 (2.49-6.59)	60.21 (52.81-65.39)	<0.0001	<0.0001	<0.0001	<0.0001	0.854 [0.804-0.872]
FM (%)	21.90 (18.30-29.00)	11.30 (7.00-15.20)	52.50 (48.90-55.50)	<0.0001	0.0001	<0.0001	<0.0001	0.802 [0.746-0.852]
SBP (mmHg)	115.00 (110.00-120.00)	100.00 (90.00-106.25)	130.00 (120.00-135.00)	<0.0001	<0.0001	<0.0001	<0.0001	0.573 [0.478-0.673]
DBP (mmHg)	70.00 (65.00-72.00)	60.00 (60.00-70.00)	80.00 (80.00-85.00)	<0.0001	0.1558	<0.0001	<0.0001	0.415 [0.271-0.574]
HR (beats/min)	70.00 (69.00-73.00)	72.50 (64.00-84.25)	88.00 (80.00-96.00)	<0.0001	0.4071	<0.0001	0.0002	0.344 [0.256-0.533]
mREE (Kcal)	1409.00 (1300.00-1555.00)	1222.50 (1193.25-1270.75)	1884.00 (1762.00-2058.00)	<0.0001	<0.0001	<0.0001	<0.0001	0.654 [0.566-0.730]
Glucose (mg/dL)	87.00 (82.00-90.00)	77.50 (71.50-84.00)	86.00 (82.00-93.00)	<0.0001	<0.0001	1.0000	<0.0001	0.181 [0.059-0.323]
Insulin (mU/L)	6.60 (5.10-9.00)	3.40 (2.30-6.70)	20.10 (12.90-28.10)	<0.0001	0.0104	<0.0001	<0.0001	0.508 [0.381-0.633]
HOMA-IR	1.55 (1.06-1.84)	0.6732 (0.4249-1.22)	4.43 (2.55-5.83)	<0.0001	0.0047	<0.0001	<0.0001	0.507 [0.386-0.656]
T-C (mg/dL)	174.00 (157.00-201.00)	188.00 (150.50-217.00)	170.00 (136.00-191.00)	0.1260	1.0000	0.4106	0.1558	0.019 [0.000-0.146]
HDL-C (mg/dL)	68.00 (62.00-73.00)	71.50 (63.75-84.25)	43.00 (36.00-50.00)	<0.0001	0.7216	<0.0001	<0.0001	0.513 [0.384-0.632]
LDL-C (mg/dL)	109.00 (86.00-121.00)	106.50 (79.25-125.25)	106.00 (80.00-125.00)	0.9403	1.0000	1.0000	1.0000	0.000 [0.000-0.061]
TG (mg/dL)	64.00 (54.00-87.00)	79.00 (61.50-92.25)	109.00 (89.00-137.00)	<0.0001	1.0000	<0.0001	0.0002	0.261 [0.124-0.418]
HbA1c (%)	5.10 (5.00-5.20)	5.05 (4.60-5.20)	5.30 (5.10-5.40)	0.0004	0.4014	0.0155	0.0004	0.125 [0.032-0.227]
CRP (mg/dL)	0.1000 (0.1000-0.2000)	0.0000 (0.0000-0.0000)	0.6000 (0.4000-1.10)	<0.0001	0.0003	<0.0001	<0.0001	0.609 [0.533-0.752]

Continuous variables are reported as median (interquartile range, IQR). Differences among groups were assessed using the Kruskal-Wallis test, followed by Dunn *post hoc* pairwise comparisons. The effect size for the overall comparison is reported as epsilon-squared (epsilon2) with bootstrapped 95% confidence intervals. For abbreviations, see the abbreviation list.

The three groups differed significantly in age, although the magnitude of this difference was smaller than the anthropometric and metabolic contrasts: patients with AN were younger than subjects with NWH and OB, whereas individuals with NWH and OB did not differ significantly in age.

Body-size and body-composition variables differed among groups. Body weight and BMI showed the expected three-level distribution, with the lowest values in AN, intermediate values in NWH, and the highest values in OB. The global effect sizes were very large for both weight (ϵ² = 0.863 [0.812-0.879]) and BMI (ϵ² = 0.868 [0.833-0.883]), and all pairwise comparisons were significant. FM (expressed in kilograms and percentage) followed the same direction, with lower values in AN and higher values in OB. Conversely, the FFM percentage was highest in AN and lowest in OB, consistent with the opposite adiposity distribution across the groups. Absolute FFM (i.e., kg) was also significantly different, with the lowest in AN and the highest in OB, indicating that the group contrast involved both fat and lean compartments.

Hemodynamic variables differed among groups. SBP was lowest in AN, intermediate in NWH, and highest in OB; all pairwise comparisons were significant. DBP and HR were particularly displaced in OB: DBP was significantly higher in OB than in both NWH and AN, whereas the AN-NWH contrast for DBP did not reach significance after pairwise adjustment. HR was also highest in OB and differed from both NWH and AN, while NWH and AN were not significantly different. Measured resting energy expenditure (REE) showed the expected body-size-associated gradient, with the lowest values in AN and the highest values in OB.

The biochemical variables differed among groups. Glucose was lower in AN than in both NWH and OB, whereas NWH and OB showed overlapping fasting glucose values. Insulin and HOMA-IR were lowest in AN, intermediate in NWH, and highest in OB, with all pairwise comparisons significant. This pattern indicates that the OB group exhibited the most pronounced insulin-resistance phenotype, whereas AN was characterized by reduced insulin exposure and low HOMA-IR values.

The lipid profile was selectively altered. TC and LDL-C did not differ significantly among groups, indicating that these variables did not drive the strongest biochemical separation. HDL-C was lower in OB than in both NWH and AN, while NWH and AN did not differ significantly. TG were highest in OB and significantly higher than in both other groups, whereas NWH and AN did not differ significantly. HbA_1c_ showed a more modest but significant group effect, driven by higher values in OB than in AN and NWH. CRP was lowest in AN and highest in OB, with all pairwise comparisons significant.

Overall, **Table 1** shows significant between-group differences across multiple anthropometric, hemodynamic, biochemical, and inflammatory variables.

### 3.2 Comparisons in sphingolipid species and ratios

**Table 2** reports the complete plasma sphingolipid profile and ceramide ratio indices across NWH, AN, and OB. After Benjamini-Hochberg FDR correction, 38 of 45 sphingolipid variables remained significantly different among groups. These variables included ceramides, dihydroceramides, sphingomyelins, glycosphingolipids, sphingoid-base-related metabolites, and ceramide ratio indices.

**Table 2:** Descriptive statistics and group comparisons of plasma sphingolipids in normal-weight healthy (NWH) subjects and patients with anorexia nervosa (AN) or obesity (OB).

Variable	NWH	AN	OB	p_overall	p_overall_FDR	NWH vs AN	NWH vs OB	AN vs OB	Effect size (ϵ²) [95% CI]
Cer 14	0.0161 (0.0136-0.0200)	0.0183 (0.0150-0.0211)	0.0137 (0.0107-0.0170)	0.0171	0.0202	0.4049	0.3023	0.0135	0.055 [0.001-0.190]
Cer 16	0.4666 (0.4008-0.5179)	0.5107 (0.4553-0.5888)	0.4298 (0.3882-0.5567)	0.0963	0.1057	0.2947	1.0000	0.1048	0.024 [0.000-0.171]
Cer 18	0.0728 (0.0642-0.1076)	0.0911 (0.0616-0.1186)	0.1184 (0.0960-0.1581)	<0.0001	<0.0001	0.5461	<0.0001	0.0203	0.172 [0.072-0.321]
Cer 18:1	0.0149 (0.0114-0.0167)	0.0137 (0.0093-0.0166)	0.0145 (0.0123-0.0171)	0.3095	0.3166	0.4821	1.0000	0.5455	0.003 [0.000-0.079]
Cer 20	0.0914 (0.0699-0.1375)	0.1826 (0.1402-0.2037)	0.0796 (0.0713-0.1076)	<0.0001	<0.0001	<0.0001	0.8992	<0.0001	0.334 [0.237-0.449]
Cer 22	0.6215 (0.4542-0.6880)	0.5233 (0.3785-0.6082)	0.4087 (0.3471-0.6150)	0.0061	0.0086	0.0651	0.0097	1.0000	0.074 [0.007-0.207]
Cer 24	3.83 (3.09-4.75)	4.67 (3.30-5.68)	2.29 (1.74-3.16)	<0.0001	<0.0001	0.5636	<0.0001	<0.0001	0.295 [0.206-0.458]
Cer 24:1	0.8953 (0.7248-1.02)	1.66 (1.52-1.81)	1.02 (0.8418-1.36)	<0.0001	<0.0001	<0.0001	0.0795	<0.0001	0.487 [0.343-0.584]
DHCer 16	0.0233 (0.0189-0.0322)	0.0174 (0.0144-0.0244)	0.0274 (0.0219-0.0356)	0.0006	0.0009	0.0560	0.1735	0.0003	0.117 [0.047-0.229]
DHCer 18	0.0060 (0.0040-0.0077)	0.0067 (0.0050-0.0094)	0.0187 (0.0120-0.0250)	<0.0001	<0.0001	1.0000	<0.0001	<0.0001	0.372 [0.266-0.514]
DHCer 18:1	0.0046 (0.0015-0.0061)	0.0000 (0.0000-0.0000)	0.0084 (0.0054-0.0130)	<0.0001	<0.0001	<0.0001	0.0011	<0.0001	0.546 [0.442-0.633]
DHCer 24	0.1810 (0.1609-0.2434)	0.1607 (0.1185-0.2058)	0.1951 (0.1254-0.3009)	0.0678	0.0762	0.1504	1.0000	0.0895	0.030 [0.000-0.130]
DHCer 24:1	0.0759 (0.0600-0.1110)	0.1424 (0.1253-0.1653)	0.1870 (0.1178-0.2420)	<0.0001	<0.0001	<0.0001	<0.0001	1.0000	0.294 [0.181-0.478]
SM 16	122.48 (117.79-142.47)	199.39 (181.43-210.67)	116.15 (106.53-131.01)	<0.0001	<0.0001	<0.0001	0.0930	<0.0001	0.522 [0.419-0.640]
SM 18	37.00 (31.68-47.53)	48.60 (34.10-56.53)	44.31 (33.08-51.30)	0.0118	0.0152	0.0159	0.1161	1.0000	0.062 [0.000-0.156]
SM 18:1	23.31 (19.98-27.24)	27.54 (21.63-32.92)	29.56 (26.75-33.20)	<0.0001	0.0001	0.0732	<0.0001	0.3314	0.151 [0.062-0.292]
SM 24	24.93 (12.30-53.18)	74.60 (65.88-88.86)	14.96 (9.19-25.41)	<0.0001	<0.0001	<0.0001	0.0091	<0.0001	0.489 [0.353-0.571]
SM 24:1	46.88 (31.42-67.26)	167.73 (142.94-185.81)	49.89 (37.77-60.90)	<0.0001	<0.0001	<0.0001	1.0000	<0.0001	0.536 [0.418-0.635]
HexCer 16	1.49 (1.22-1.71)	1.44 (1.21-1.69)	1.42 (1.24-1.81)	0.9122	0.9122	1.0000	1.0000	1.0000	0.000 [0.000-0.031]
HexCer 18	0.2449 (0.2014-0.3335)	0.2578 (0.2045-0.3442)	0.2299 (0.1889-0.2616)	0.0571	0.0659	1.0000	0.1160	0.1151	0.034 [0.000-0.122]
HexCer 18:1	0.0176 (0.0109-0.0410)	0.0056 (0.0038-0.0090)	0.0251 (0.0192-0.0435)	<0.0001	<0.0001	<0.0001	0.2702	<0.0001	0.417 [0.270-0.516]
HexCer 20	0.2959 (0.2658-0.3970)	0.3187 (0.2628-0.3875)	0.2608 (0.2207-0.3001)	0.0089	0.0121	1.0000	0.0351	0.0165	0.067 [0.020-0.176]
HexCer 22	4.10 (3.78-5.22)	4.02 (3.56-5.18)	2.74 (2.06-3.57)	<0.0001	<0.0001	0.7989	<0.0001	<0.0001	0.344 [0.184-0.509]
HexCer 24	4.74 (4.22-5.87)	4.75 (4.46-6.27)	3.09 (2.35-4.39)	<0.0001	<0.0001	1.0000	0.0001	<0.0001	0.206 [0.059-0.392]
HexCer 24:1	3.88 (3.37-5.67)	6.91 (5.98-7.97)	5.07 (4.10-6.68)	<0.0001	<0.0001	<0.0001	0.0330	0.0010	0.320 [0.216-0.448]
LacCer 16	7.54 (5.89-10.02)	6.51 (5.74-7.52)	7.26 (6.25-10.27)	0.1162	0.1245	0.1606	1.0000	0.2309	0.021 [0.000-0.121]
LacCer 18	0.1283 (0.0956-0.2091)	0.0975 (0.0775-0.1154)	0.1199 (0.0875-0.2026)	0.0053	0.0077	0.0047	1.0000	0.0426	0.076 [0.025-0.206]
LacCer 18:1	0.0535 (0.0324-0.0713)	0.0692 (0.0500-0.0917)	0.0470 (0.0324-0.0746)	0.0093	0.0123	0.0193	1.0000	0.0179	0.066 [0.025-0.167]
LacCer 20	0.0833 (0.0324-0.1183)	0.1000 (0.0683-0.1204)	0.0373 (0.0243-0.0924)	0.0002	0.0003	0.2440	0.0167	0.0001	0.137 [0.048-0.263]
LacCer 22	0.3582 (0.0810-0.4700)	0.4358 (0.3308-0.5079)	0.0794 (0.0389-0.2334)	<0.0001	<0.0001	0.0681	0.0015	<0.0001	0.234 [0.163-0.393]
LacCer 24	0.1653 (0.0081-0.3617)	0.4017 (0.3279-0.5200)	0.0130 (0.0032-0.1329)	<0.0001	<0.0001	0.0007	0.0351	<0.0001	0.274 [0.156-0.402]
LacCer 24:1	1.29 (0.1815-1.98)	2.78 (2.20-3.32)	0.1556 (0.0956-1.24)	<0.0001	<0.0001	0.0001	0.1257	<0.0001	0.277 [0.152-0.427]
GM3 16	2.12 (1.61-3.04)	2.46 (2.18-2.95)	1.83 (1.44-2.62)	0.0139	0.0170	0.0898	1.0000	0.0123	0.059 [0.019-0.151]
GM3 18	0.3603 (0.2800-0.6246)	0.4267 (0.3567-0.5833)	0.3603 (0.2162-0.6006)	0.2029	0.2124	0.7226	1.0000	0.2245	0.011 [0.000-0.099]
GM3 18:1	0.0240 (0.0000-0.0240)	0.0000 (0.0000-0.0000)	0.0240 (0.0000-0.0480)	<0.0001	<0.0001	<0.0001	1.0000	<0.0001	0.332 [0.233-0.451]
GM3 20	0.1201 (0.0480-0.1467)	0.2067 (0.1600-0.2667)	0.0721 (0.0480-0.1441)	<0.0001	<0.0001	0.0003	0.9450	<0.0001	0.188 [0.085-0.309]
GM3 22	1.13 (0.6667-1.71)	0.6600 (0.5700-0.9500)	0.6246 (0.4084-0.9129)	0.0007	0.0011	0.0226	0.0012	1.0000	0.112 [0.008-0.250]
GM3 24	0.2162 (0.1441-0.3123)	0.3067 (0.2233-0.4300)	0.0961 (0.0480-0.1261)	<0.0001	<0.0001	0.0929	<0.0001	<0.0001	0.336 [0.206-0.488]
GM3 24:1	0.9609 (0.6726-1.35)	1.45 (1.10-2.20)	0.9849 (0.7447-1.36)	0.0002	0.0003	0.0002	1.0000	0.0028	0.134 [0.057-0.271]
S1P	1.73 (1.14-2.17)	2.45 (1.61-3.31)	1.76 (1.39-2.10)	0.0129	0.0161	0.0214	1.0000	0.0293	0.060 [0.000-0.157]
DhSph	0.0138 (0.0049-0.0171)	0.0000 (0.0000-0.0000)	0.0190 (0.0143-0.0246)	<0.0001	<0.0001	<0.0001	0.0097	<0.0001	0.508 [0.398-0.641]
DhS1P	0.3855 (0.2814-0.4892)	0.9781 (0.6939-1.39)	0.3286 (0.2550-0.3883)	<0.0001	<0.0001	<0.0001	0.0890	<0.0001	0.358 [0.248-0.487]
CER16/24	0.1243 (0.1065-0.1505)	0.1224 (0.0965-0.1392)	0.1997 (0.1592-0.2408)	<0.0001	<0.0001	1.0000	<0.0001	<0.0001	0.342 [0.182-0.450]
CER18/24	0.0193 (0.0167-0.0248)	0.0223 (0.0173-0.0236)	0.0543 (0.0405-0.0642)	<0.0001	<0.0001	1.0000	<0.0001	<0.0001	0.602 [0.537-0.689]
CER24:1/24	0.2191 (0.1981-0.2381)	0.4165 (0.3052-0.4811)	0.4517 (0.3528-0.5347)	<0.0001	<0.0001	<0.0001	<0.0001	1.0000	0.643 [0.559-0.700]

Continuous variables are summarized as median (interquartile range, IQR). Differences among the three study groups were assessed using the Kruskal-Wallis rank-sum test. To account for multiple testing across analytes, overall p-values were adjusted using the Benjamini-Hochberg false discovery rate (FDR) procedure. Pairwise comparisons were performed using Dunn *post hoc* tests. Effect size is reported as epsilon-squared (epsilon2) with bootstrapped 95% confidence intervals. For abbreviations, see the abbreviation list.

Among ceramides, the FDR-significant variables were Cer 14, Cer 18, Cer 20, Cer 22, Cer 24, and Cer 24:1. Their distribution was heterogeneous rather than uniformly increased or decreased in one group. Cer 18 was highest in OB and significantly higher than both NWH and AN, whereas Cer 20 and Cer 24:1 were significantly higher in AN than in both NWH and OB. Cer 24 was lowest in OB and differed from both NWH and AN, while Cer 22 was also reduced in OB compared with NWH. Thus, the ceramide compartment showed both obesity-associated enrichment of selected shorter-chain species and anorexia-associated increases in specific very-long-chain or monounsaturated species, resulting in a complex class-specific pattern rather than a single-direction shift.

DHCer 16, DHCer 18, DHCer 18:1, and DHCer 24:1 remained significant after FDR correction. DHCer 18 and DHCer 18:1 were highest in OB, and DHCer 18 differed significantly between OB and both NWH and AN. DHCer 18:1 was nearly absent or very low in AN and clearly higher in OB, while DHCer 24:1 was elevated in both AN and OB compared with NWH. These findings indicate that the dihydroceramide branch contributed substantially to group separation, particularly in distinguishing OB from the other two groups.

SM 16, SM 18, SM 18:1, SM 24, and SM 24:1 differed significantly among groups after FDR correction. SM 16, SM 24, and SM 24:1 were higher in AN than in OB and were also higher in AN than in NWH. Therefore, sphingomyelins contributed importantly to the discrimination of AN from OB, while selected species also contributed to the OB-NWH contrast.

Several glycosphingolipid-related variables differed among groups. HexCer 18:1, HexCer 20, HexCer 22, HexCer 24, and HexCer 24:1 were significant after FDR correction. HexCer 22 and HexCer 24 were lower in OB than in both NWH and AN, whereas HexCer 24:1 was highest in AN and remained significantly different from both NWH and OB. LacCer 18, LacCer 18:1, LacCer 20, LacCer 22, LacCer 24, and LacCer 24:1 also differed significantly among groups after FDR correction. Several LacCer species, particularly LacCer 22, LacCer 24, and LacCer 24:1, were highest in AN and lowest in OB, with significant differences between AN and OB. GM3 species showed a similarly structured pattern, with GM3 18:1, GM3 20, GM3 22, GM3 24, and GM3 24:1 contributing to the significant signature. Overall, glycosphingolipids strongly separated AN and OB and also identified selected differences between OB and NWH.

Sphingoid-base-related variables were also involved. S1P, DhSph, and DhS1P retained FDR significance. S1P and DhS1P were higher in AN than in NWH and OB, whereas DhSph was lowest in AN and highest in OB. These opposing trends indicate that even small sphingoid intermediates contributed to the dataset’s multicomponent lipidomic architecture.

Ceramide ratio indices showed some of the strongest group effects across the entire lipidomic panel. CER16/24, CER18/24, and CER24:1/24 all remained significant after FDR correction. CER18/24 and CER24:1/24 had among the largest effect sizes (respectively 0.602 [0.537-0.689] and 0.643 [0.559-0.700]). CER18/24 was markedly higher in OB than in both NWH and AN, whereas CER24:1/24 was increased in both AN and OB compared with NWH, with no significant AN-OB difference. CER16/24 was higher in OB than in both NWH and AN. These ratio variables, therefore, captured relative remodeling of ceramide chain-length composition and provided a particularly strong statistical contribution to group discrimination.

Taken together, **Table 2** indicates that a generalized monotonic shift of all sphingolipids did not distinguish the three groups. Instead, the significant signature consisted of coordinated but class-specific changes: several AN-enriched sphingomyelins, lactosylceramides, GM3 species, and DhS1P; several OB-enriched dihydroceramides and ceramide ratios; and a subset of OB-depleted very-long-chain ceramide/glycosphingolipid species. This pattern explains why subsequent multivariate analyses were able to separate the groups using the FDR-significant subset of lipids.

**Supplementary Figures S2–S39** in the **Supplementary Material** represent boxplots of significant sphingolipid species and ratios across NWH, AN, and OB groups.

**Figure 1** summarizes the same FDR-significant variables as a z-score heatmap, with subjects ordered by group. This visualization emphasizes the coordinated structure underlying the univariate results. The samples from the AN group displayed a block-like pattern of high standardized values for several sphingomyelins and glycosphingolipids, including SM 16, SM 24, SM 24:1, LacCer 22, LacCer 24, LacCer 24:1, GM3 20, GM3 24:1, S1P, and DhS1P. Conversely, OB samples showed higher standardized values for DHCer 18, DHCer 18:1, DhSph, CER16/24, and CER18/24, together with lower values for several long-chain or very-long-chain glycosphingolipid species. NWH subjects showed a more mixed and generally less extreme profile, with many values closer to the central z-score range.

**Figure 1:**
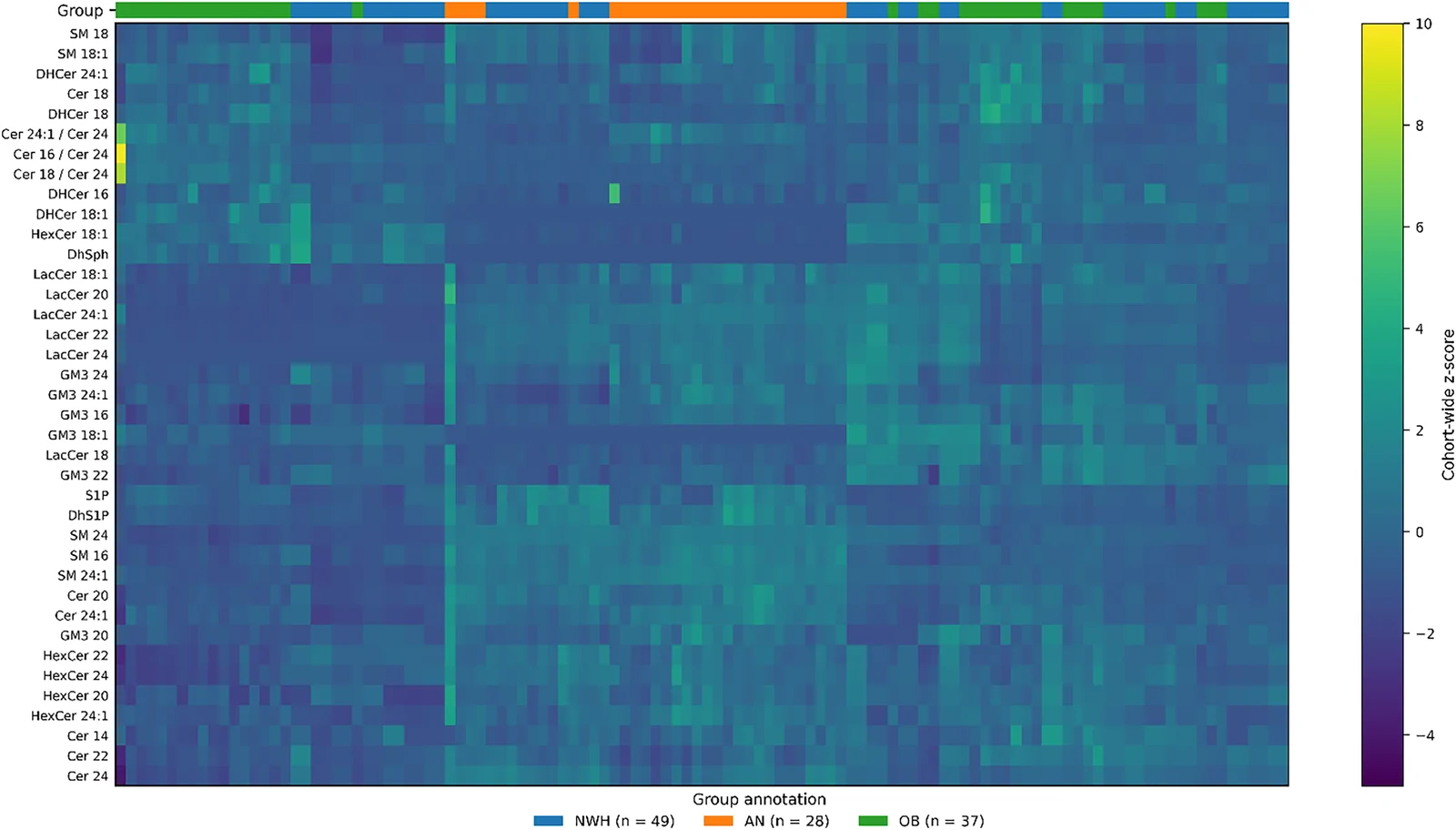
Heatmap of cohort-wide z-score-standardized significant sphingolipid variables across all participants. Rows represent significant sphingolipid variables, and columns represent individual participants. Data were log-transformed and standardized. The annotation bar above the columns indicates group membership (AN, anorexia nervosa; NWH, normal-weight healthy; OB, obesity). Cell colors represent z-scores calculated for each variable across the entire cohort, not relative to NWH controls; therefore, colors indicate values above or below the cohort-wide mean and should not be interpreted as fold changes relative to controls.

The heatmap, therefore, indicates, from an exploratory perspective, that the lipidomic signature generated a structured separation among the three phenotypes. AN and OB were the most divergent groups because they showed opposite color patterns across multiple lipid blocks. NWH did not form a uniformly extreme cluster but instead appeared as a reference distribution that partially overlapped with either AN or OB, depending on the lipid class. This observation is consistent with the idea that the significant sphingolipid panel captures nutritional-state-dependent remodeling of plasma sphingolipid composition.

### 3.3 Exploratory lipidomic visualization: all sphingolipid variables

#### 3.3.1 Volcano plot analysis of sphingolipid variables across NWH, AN, and OB groups

Volcano plot analysis was performed on all sphingolipid variables reported in **Table 2**, including both FDR-significant and non-significant variables.

The volcano analyses identified FDR-significant differences in each pairwise comparison, with the largest number of significant variables observed between OB and AN. Importantly, the directionality of the altered features was not homogeneous across lipids, indicating class- and species-selective lipidomic shifts rather than a uniform increase or decrease in sphingolipid abundance.

Compared with NWH, AN showed 25 sphingolipid variables that remained significant after FDR correction. Most significant features were shifted toward higher median values in AN, whereas a smaller but biologically coherent cluster was reduced. The most prominent AN-enriched signals involved long-chain/very-long-chain ceramide-related and sphingomyelin species, particularly Cer 24:1, SM 24:1, CER24:1/24, and SM 16. In contrast, DHCer 18:1, DhSph, GM3 18:1, and HexCer 18:1 were among the most strongly reduced features in AN. This pattern suggests that AN is associated with a marked enrichment of selected very-long-chain ceramide/sphingomyelin-related signals, accompanied by depletion of specific sphingoid-base and dihydroceramide-related components (**Figure 2**).

**Figure 2:**
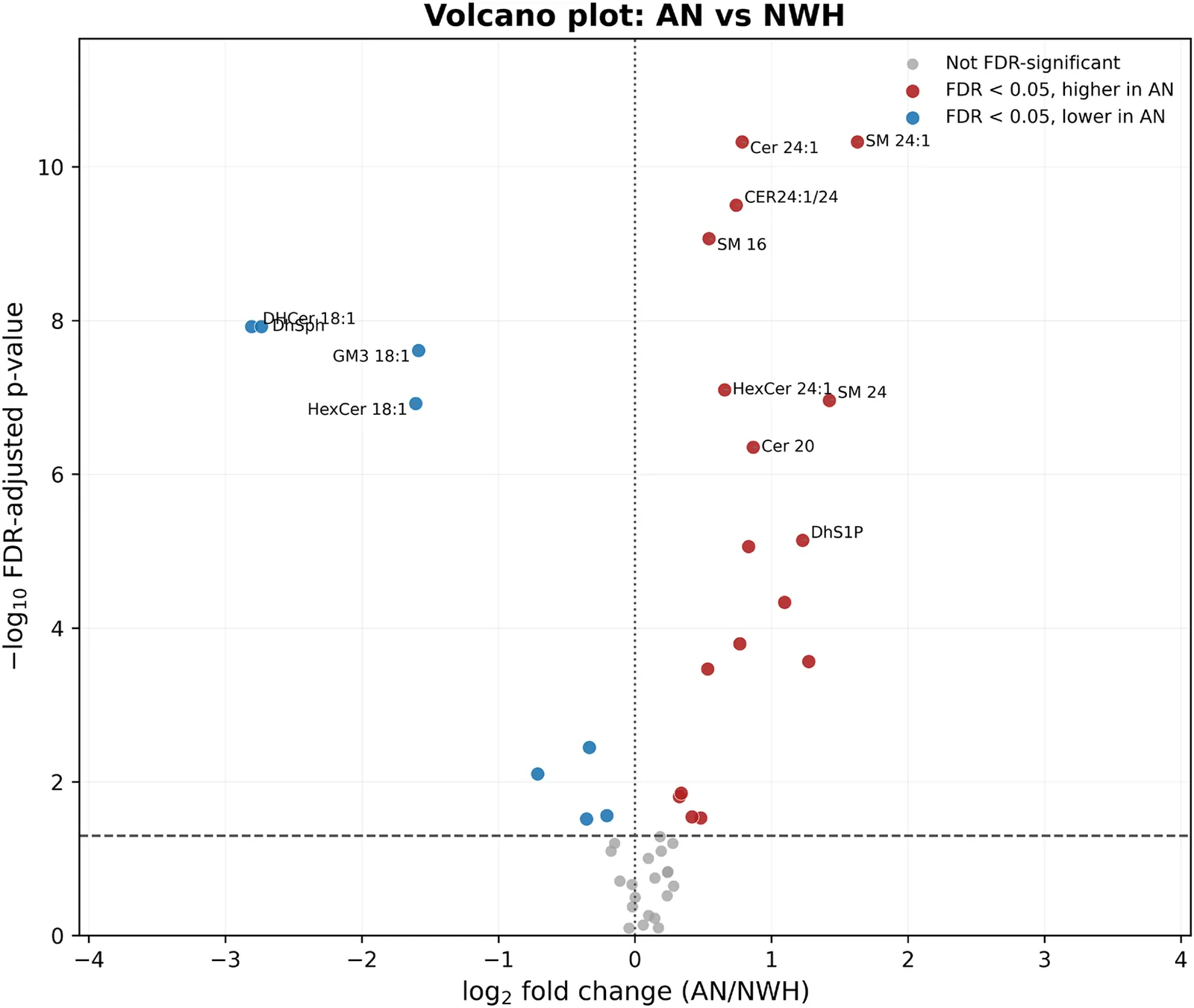
Volcano plot of the AN versus NWH comparison. Each point represents one sphingolipid variable. The x-axis reports the median-based log2 fold change for the comparison shown in the plot title. The y-axis reports −log10 of the FDR-adjusted pairwise p-value. Red points denote variables significantly higher in the numerator group of the comparison, blue points denote variables significantly lower in that group, and grey points denote variables not significant after FDR correction. The vertical dotted line indicates log2FC = 0 and therefore no median shift between groups. The horizontal dashed line corresponds to FDR = 0.05; variables located above this line are significant after Benjamini-Hochberg correction. Labels are shown for the most statistically prominent features to improve interpretability while avoiding overcrowding. The analysis includes all sphingolipid variables, regardless of whether they were significant in the global three-group table.

The OB versus NWH comparison also identified 25 FDR-significant variables, but the volcano distribution differed from the AN pattern. In OB, the most statistically robust features were ceramide ratio indices, especially CER18/24 and CER24:1/24, together with higher DHCer 18 and DHCer 24:1. At the same time, several complex glycosphingolipids and very-long-chain species were lower in OB, including HexCer 22, Cer 24, GM3 24, HexCer 24, and LacCer 22. Thus, the OB phenotype appears to be characterized by a relative shift in ceramide acyl-chain balance, with enrichment of specific ceramide ratios and dihydroceramide species rather than the global sphingomyelin-rich profile observed in AN (**Figure 3**).

**Figure 3:**
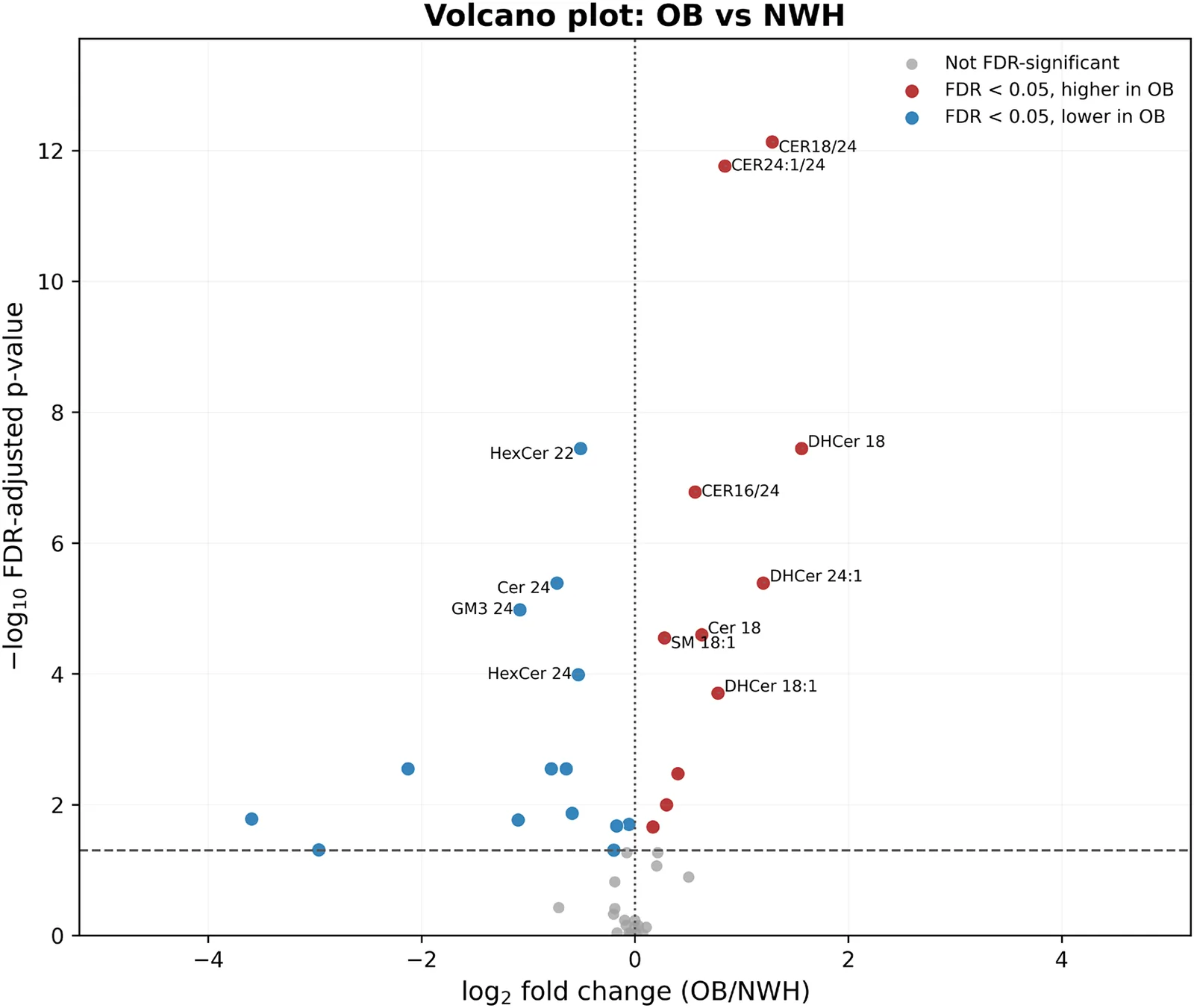
Volcano plot of the OB versus NWH comparison. Each point represents one sphingolipid variable. The x-axis reports the median-based log2 fold change for the comparison shown in the plot title. The y-axis reports −log10 of the FDR-adjusted pairwise p-value. Red points denote variables significantly higher in the numerator group of the comparison, blue points denote variables significantly lower in that group, and grey points denote variables not significant after FDR correction. The vertical dotted line indicates log2FC = 0 and therefore no median shift between groups. The horizontal dashed line corresponds to FDR = 0.05; variables located above this line are significant after Benjamini-Hochberg correction. Labels are shown for the most statistically prominent features to improve interpretability while avoiding overcrowding. The analysis includes all sphingolipid variables, regardless of whether they were significant in the global three-group table.

The direct OB versus AN comparison produced the largest number of FDR-significant variables, with 32 altered sphingolipid features. This finding indicates that the lipidomic divergence between the two pathological phenotypes exceeds their respective divergence from NWH for several lipid classes. OB showed higher DHCer 18:1, DhSph, HexCer 18:1, CER18/24, and GM3 18:1, whereas AN showed higher SM 24:1, SM 24, SM 16, DhS1P, Cer 20, GM3 24, Cer 24:1, LacCer 22, LacCer 24, and LacCer 24:1. Overall, OB showed higher values for selected dihydroceramide, sphingoid-base, and ceramide-ratio variables, whereas AN showed higher values for several sphingomyelins and very-long-chain glycosphingolipid-related species (**Figure 4**).

**Figure 4:**
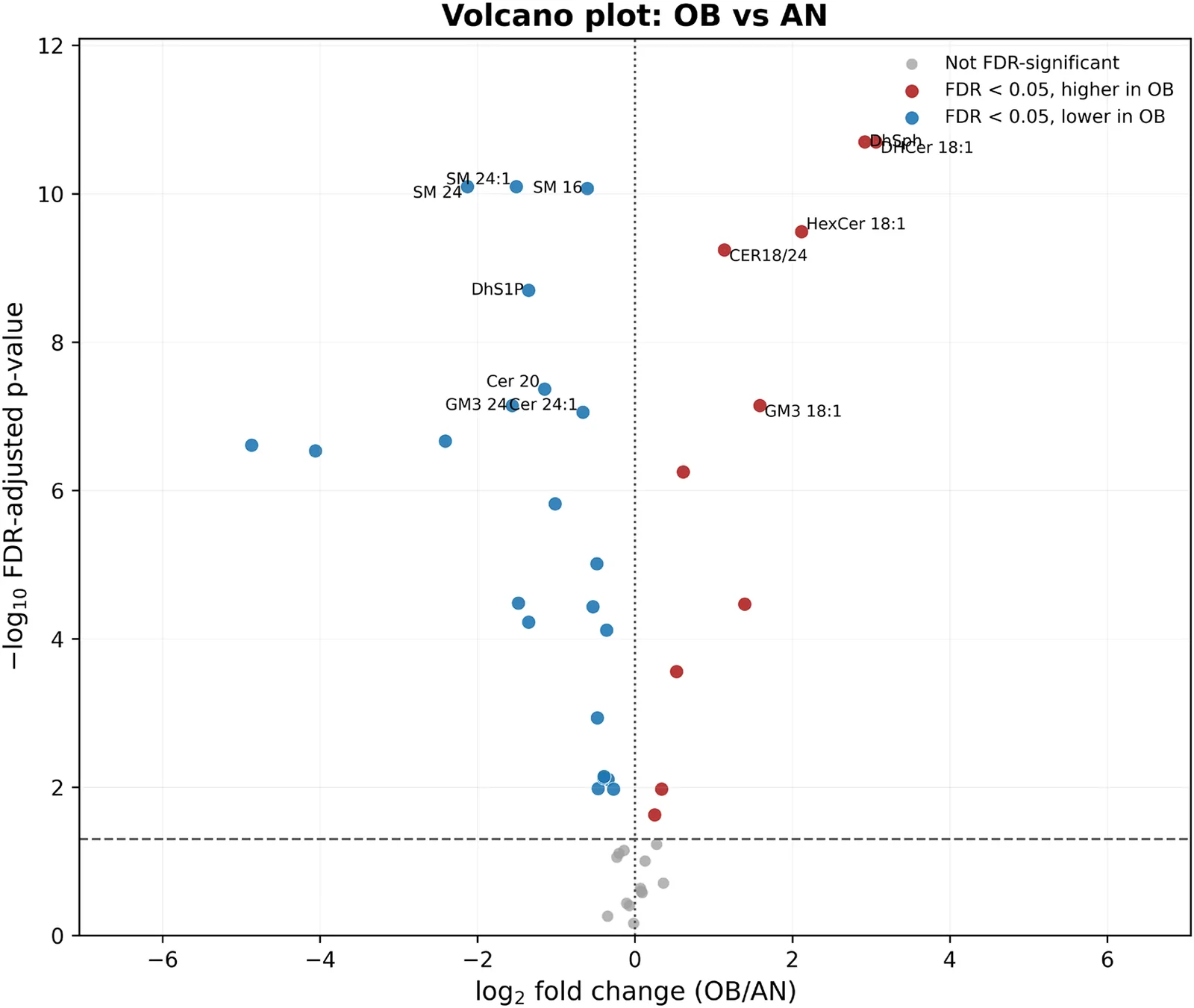
Volcano plot of the OB versus AN comparison. Each point represents one sphingolipid variable. The x-axis reports the median-based log2 fold change for the comparison shown in the plot title. The y-axis reports −log10 of the FDR-adjusted pairwise p-value. Red points denote variables significantly higher in the numerator group of the comparison, blue points denote variables significantly lower in that group, and grey points denote variables not significant after FDR correction. The vertical dotted line indicates log2FC = 0 and therefore no median shift between groups. The horizontal dashed line corresponds to FDR = 0.05; variables located above this line are significant after Benjamini-Hochberg correction. Labels are shown for the most statistically prominent features to improve interpretability while avoiding overcrowding. The analysis includes all sphingolipid variables, regardless of whether they were significant in the global three-group table.

Overall, AN and OB displayed distinct sphingolipidomic signatures rather than non-specific changes in sphingolipid variables, including circulating sphingolipid levels and sphingolipid ratios. AN was dominated by increases in selected sphingomyelins and very-long-chain ceramide-related species, with reductions in DHCer 18:1, DhSph, GM3 18:1, and HexCer 18:1. OB displayed a different profile, driven by higher ceramide ratio indices and selected dihydroceramides, together with lower levels of several very-long-chain hexosylceramide, lactosylceramide, GM3, and ceramide species. The direct OB versus AN comparison confirmed and amplified this divergence, providing the largest number of FDR-significant variables and highlighting opposite remodeling of sphingomyelin-rich, glycosphingolipid-rich, and dihydroceramide/sphingoid-base compartments.

#### 3.3.2 Principal component analysis of plasma sphingolipid variables

An unsupervised PCA was performed on the full sphingolipid panel rather than on the FDR-filtered subset alone. This strategy was used to evaluate the global sphingolipidomic structure of the dataset without imposing prior feature selection based on univariate significance. Thus, the analysis included all 45 sphingolipid variables reported in **Table 2**.

The first two principal components accounted for 32.4% and 15.8% of the total sphingolipid variance, respectively, yielding a cumulative explained variance of 48.2%. Inclusion of PC3 increased the cumulative explained variance to 58.8%. Thus, the first two axes captured a meaningful, but not exhaustive, proportion of the global sphingolipidomic variability, consistent with a complex multianalyte structure rather than a single dominant lipid axis.

In the PC1-PC2 score space, the three groups showed a structured but partially overlapping distribution. The AN centroid was displaced from NWH predominantly along the component(s) capturing variation in long-chain sphingomyelins, glycosphingolipids and selected sphingoid-base-related variables. In contrast, the OB centroid occupied a different region of the score space, reflecting a distinct multivariate lipid configuration rather than a simple linear extension of the AN pattern. Centroid distances in the first two PCs were 4.78 for NWH versus AN, 2.08 for NWH versus OB, and 6.85 for AN versus OB, supporting a greater multivariate divergence between AN and OB than between either condition and NWH in the reduced PCA plane. The presence of residual overlap indicates that group differences are not absolute at the individual-subject level, but the centroid shift suggests that nutritional extremes are associated with coordinated remodeling of plasma sphingolipid profiles (**Figure 5**).

**Figure 5:**
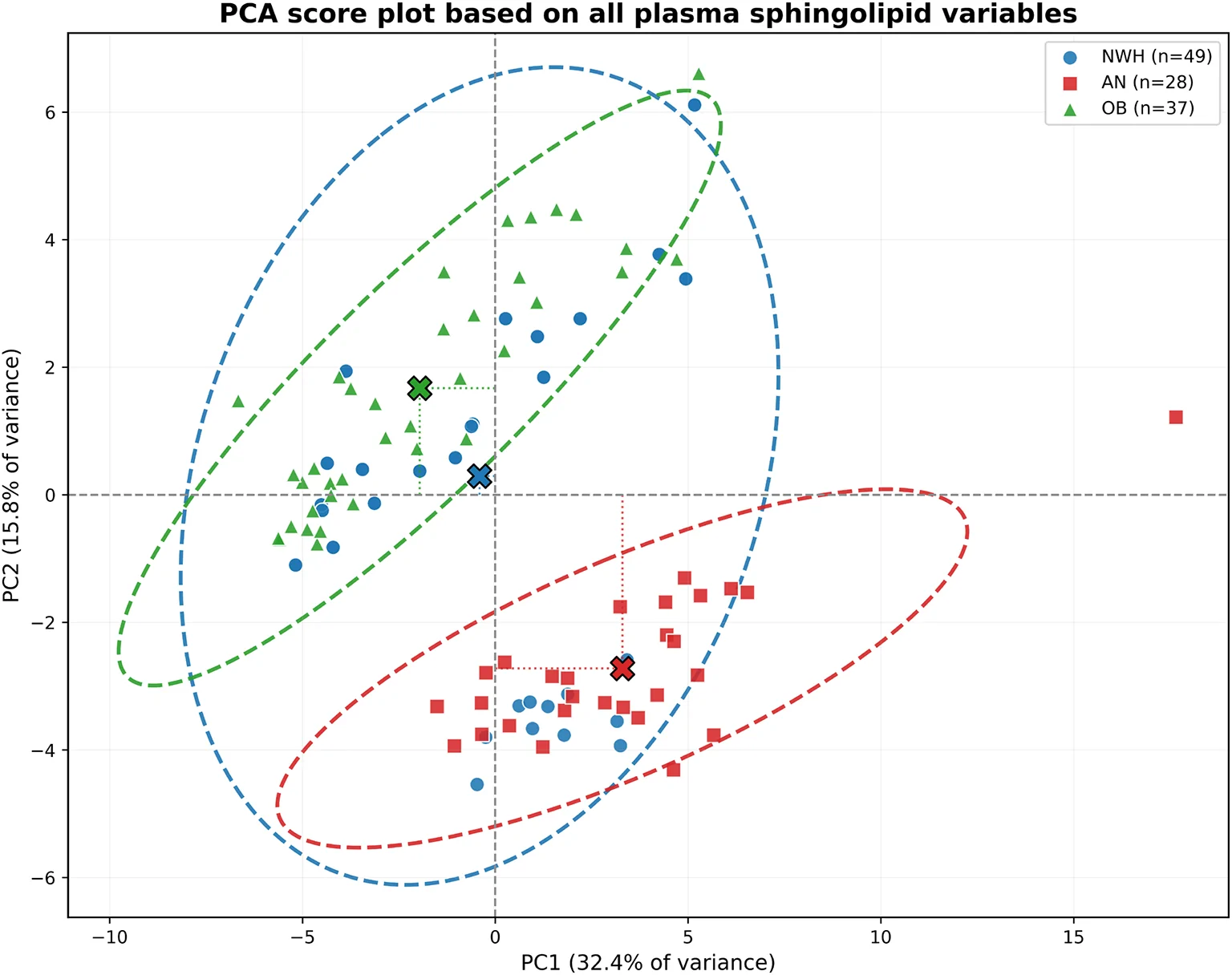
PCA score plot of all sphingolipid variables. Each point represents one subject projected onto PC1 and PC2. PC1 explains 32.4% of the total variance, whereas PC2 explains 15.8%. Subjects are colored according to experimental group (NWH, AN or OB). Group centroids are shown as large X symbols. Dashed ellipses represent 95% confidence regions around the group distribution in the PC1-PC2 plane. The grey dashed horizontal and vertical reference lines indicate zero values for PC2 and PC1, respectively, and divide the score space into quadrants. Dotted colored projection lines connect each group centroid to the PC axes, and help visualize the direction and magnitude of group displacement from the origin. Because PCA is unsupervised, separation among groups should be interpreted as the emergent structure of the sphingolipid dataset rather than as a classification result. The ellipse size reflects within-group dispersion in the multivariate score space, whereas the centroid position summarizes the average sphingolipidomic profile of each group. Overlap between ellipses indicates shared variance or biological heterogeneity, while centroid displacement indicates systematic differences in the global sphingolipid pattern.

The loading structure indicated that the first two PCs were driven by a coordinated contribution of multiple sphingolipid subclasses rather than by isolated analytes (**Figure 6**). The strongest variables in the PC1-PC2 plane included LacCer 18, SM 24, GM3 18:1, SM 24:1, LacCer 16, SM 16, GM3 22, DhS1P, DHCer 18:1, GM3 16. Variables with positive PC1 loadings included LacCer 20, HexCer 24:1, LacCer 22, LacCer 24:1, HexCer 20, SM 18, whereas negative PC1 loadings were mainly observed for DhSph, HexCer 18:1, DHCer 18:1, Cer 18/Cer 24, Cer 16/Cer 24, DHCer 16. PC2 was influenced positively by GM3 18:1, LacCer 18, LacCer 16, DHCer 18:1, GM3 22, HexCer 18:1 and negatively by DhS1P, SM 24, SM 24:1, SM 16, S1P, Cer 20. This pattern suggests that the PCA separation is not attributable to one lipid class alone, but reflects concerted variation across ceramides, dihydroceramides, sphingomyelins, glycosphingolipids, ganglioside GM3 species, sphingoid bases and ceramide-ratio indices.

**Figure 6:**
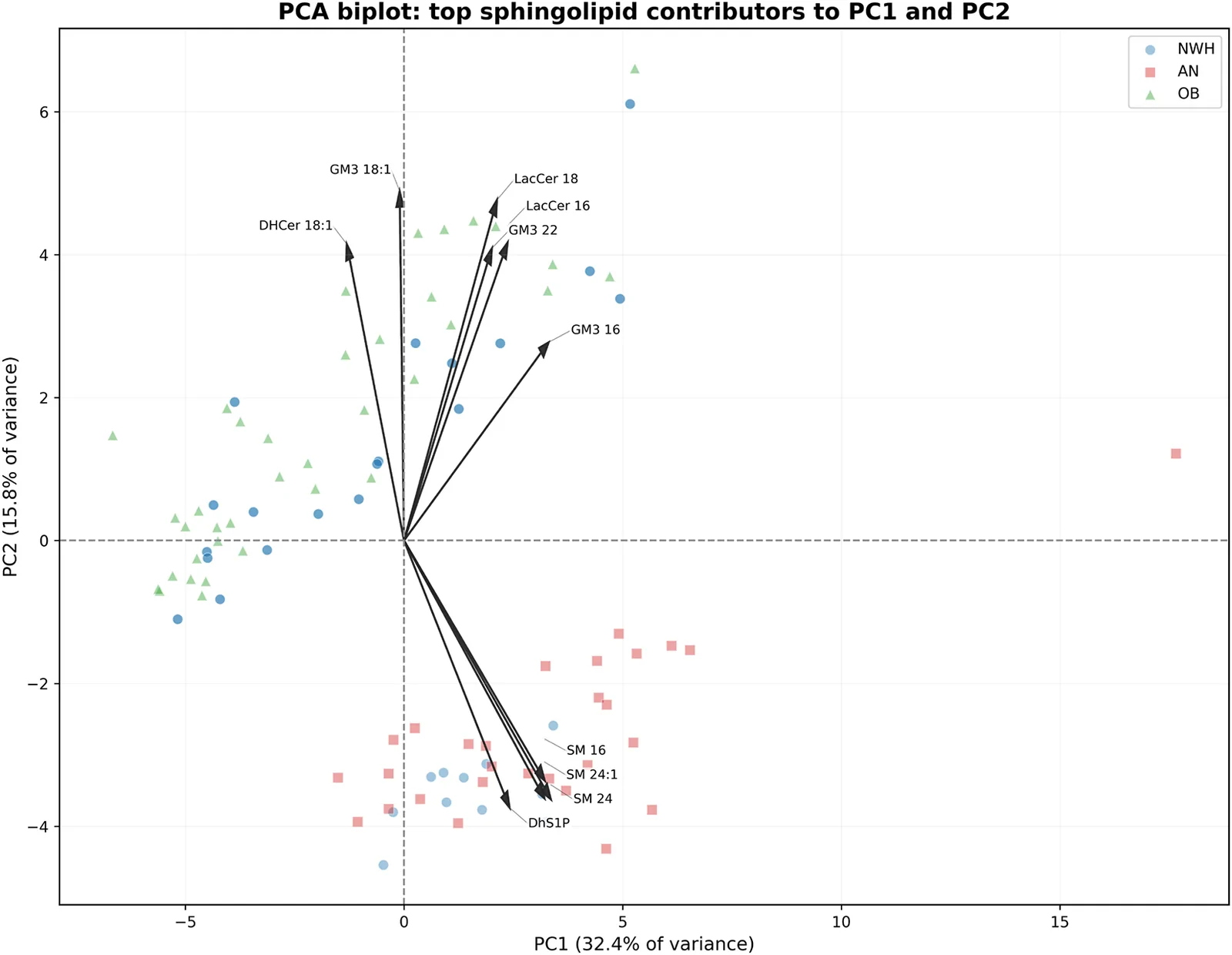
PCA biplot showing subject scores and the strongest sphingolipid contributors to PC1 and PC2. Semi-transparent points correspond to individual subjects, colored by group. Arrows represent the loading vectors for the sphingolipid variables that contribute most to PC1 and PC2. Arrow direction indicates the direction of increasing analyte abundance in the PCA space, whereas arrow length approximates the relative contribution of that variable to the displayed components. The grey dashed axes mark the PC1 = 0 and PC2 = 0 reference lines. Variables pointing in similar directions are positively correlated in the reduced PCA plane, whereas variables oriented in opposite directions contribute inversely to the main pattern of variation. The biplot should be interpreted qualitatively because only the first two PCs are displayed; variables contributing mainly to higher-order PCs are not fully represented in this figure. Labels were restricted to the top-loading variables to preserve readability and avoid overplotting.

The scree plot showed that no single principal component accounted for the majority of the total variance (**Figure 7**). PC1 explained 32.4% of the variance and PC2 explained 15.8%, while subsequent components accounted for progressively smaller proportions of variance.

**Figure 7:**
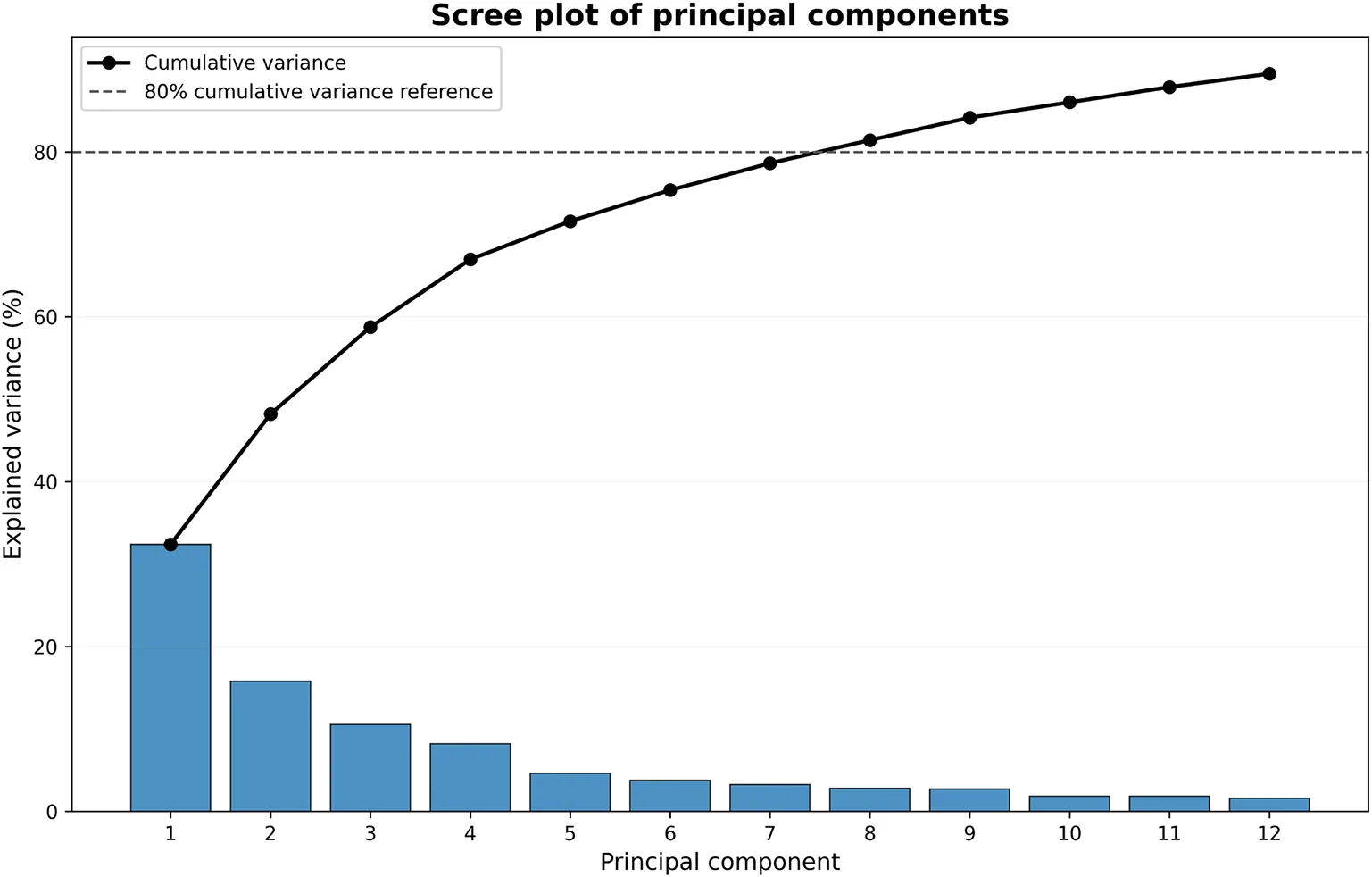
Scree plot of PCA explained variance. Bars indicate the percentage of variance explained by each principal component. The black line shows the cumulative explained variance across components. The dashed horizontal reference line marks 80% cumulative explained variance, a conventional descriptive threshold used to assess how many components are required to summarize most of the dataset’s variability. The gradual accumulation of explained variance indicates that the sphingolipidomic dataset is distributed across several correlated but non-redundant dimensions. Therefore, PC1 and PC2 are useful for visualizing the dominant multivariate structure, but they do not capture the entire biological complexity of the plasma sphingolipidome.

Overall, unsupervised PCA revealed that plasma sphingolipid profiles exhibit a multivariate structure that partially organizes subjects according to nutritional phenotype. The AN group showed a distinctive displacement characterized by variables related to sphingomyelin and selected glycosphingolipid/sphingoid-base axes. In contrast, the OB group was separated along a partially different component pattern involving ceramide-related and ratio-based features. Importantly, the observed group overlap argues against an overly deterministic interpretation and supports a more nuanced view in which AN and OB are associated with coordinated, yet heterogeneous, sphingolipidomic remodeling. These findings complement the univariate results by demonstrating that both statistically significant and non-significant analytes contribute to the overall biochemical architecture of the dataset, even when their individual effects are modest.

### 3.4 Clustering and discrimination: selected sphingolipid variables

#### 3.4.1 Hierarchical clustering analysis of FDR-significant sphingolipid variables

Hierarchical clustering based on the FDR-significant sphingolipid subset revealed a non-random organization of the lipidomic space across the three experimental groups. Overall, 35 sphingolipid variables remained statistically significant after FDR correction and were therefore used to construct the clustering matrix. The three derived ceramide ratio indices were excluded from this analysis to avoid redundancy and mathematical dependence on the parent ceramide species. This broad representation indicates that the discriminant signal was not restricted to a single lipid class, but reflected a coordinated rearrangement of several sphingolipid branches (**Figure 8**).

**Figure 8:**
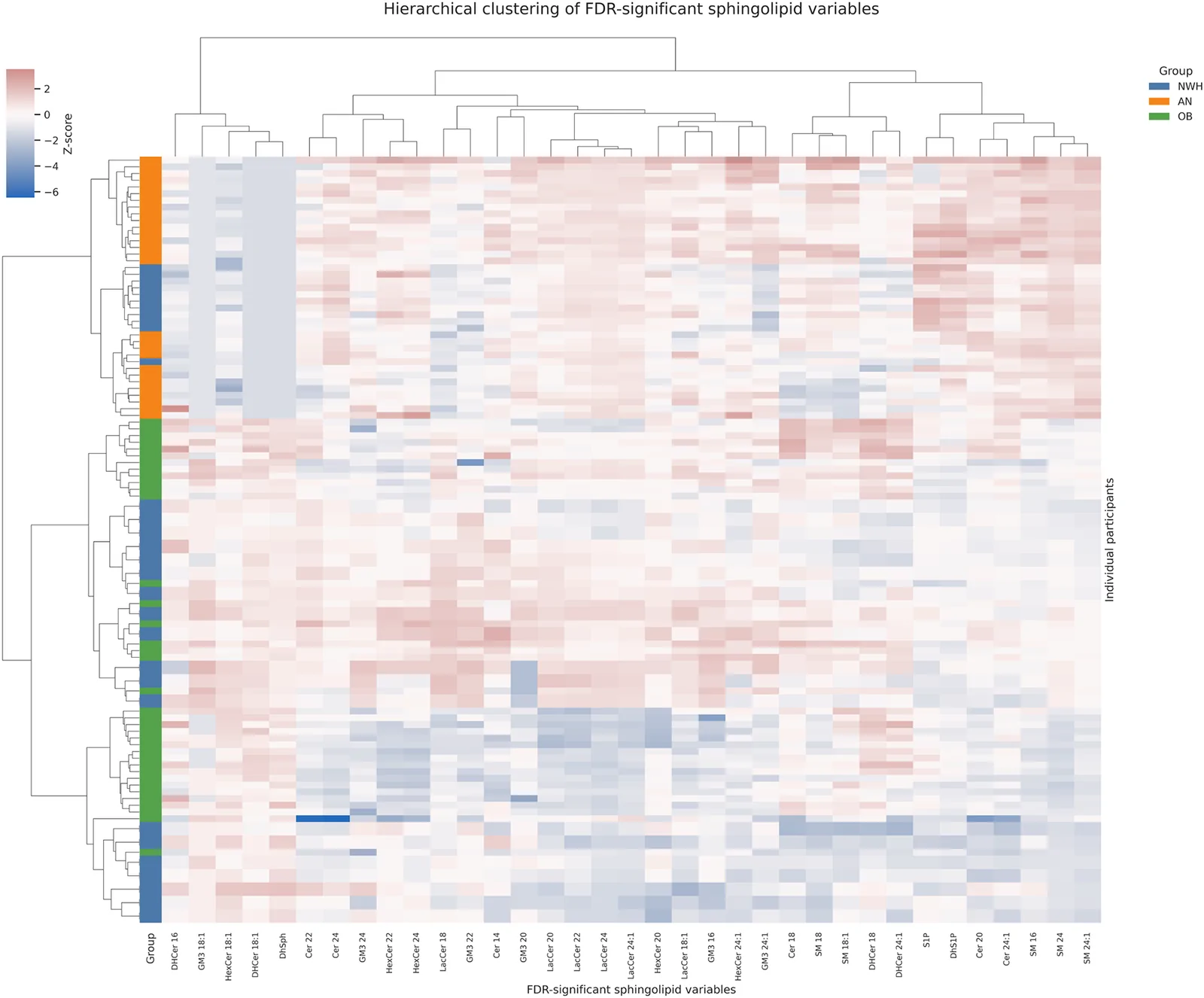
Individual-level hierarchical clustering heatmap. Unsupervised hierarchical clustering heatmap of FDR-significant sphingolipid variables across individual participants. Rows correspond to individual subjects and columns to sphingolipid variables. The analysis included only variables with an overall FDR-adjusted p-value < 0.05 in the three-group comparison. Each cell represents the standardized abundance of a given analyte after log10 transformation and Z-score scaling across subjects. Red shades indicate values above the overall mean for that variable, whereas blue shades indicate values below the overall mean. The upper dendrogram shows hierarchical relationships among sphingolipid variables; the left dendrogram shows hierarchical relationships among participants. The colored sidebar denotes group membership: NWH, AN, or OB. The branching lines in both dendrograms represent the successive agglomeration steps of the hierarchical clustering algorithm. The left (vertical) dendrogram clusters individual participants according to the similarity of their sphingolipid profiles, whereas the upper (horizontal) dendrogram clusters sphingolipid analytes according to their abundance patterns across participants. Shorter branches indicate greater similarity, whereas longer branches indicate greater dissimilarity. The color bar does not represent statistical significance but standardized relative abundance (Z-score). Group labels (NWH, AN, and OB) were not used to generate the clustering solution and are shown only as external annotations to facilitate interpretation of the unsupervised clusters.

At the group level, the heatmap showed distinct phenotype-related sphingolipid patterns across the three study groups (**Figure 9**). AN samples were characterized by a distinct lipid configuration driven by a higher relative abundance of several sphingomyelin species, selected long-chain ceramide-related species, lactosylceramides, GM3 species, and sphingoid phosphate-related measures. Conversely, OB samples showed a divergent pattern dominated by enrichment of selected dihydroceramides, along with a lower relative abundance of several complex glycosphingolipid species. NWH subjects occupied an intermediate or reference-like region of the clustered space, with partial overlap depending on the analyte module considered.

**Figure 9:**
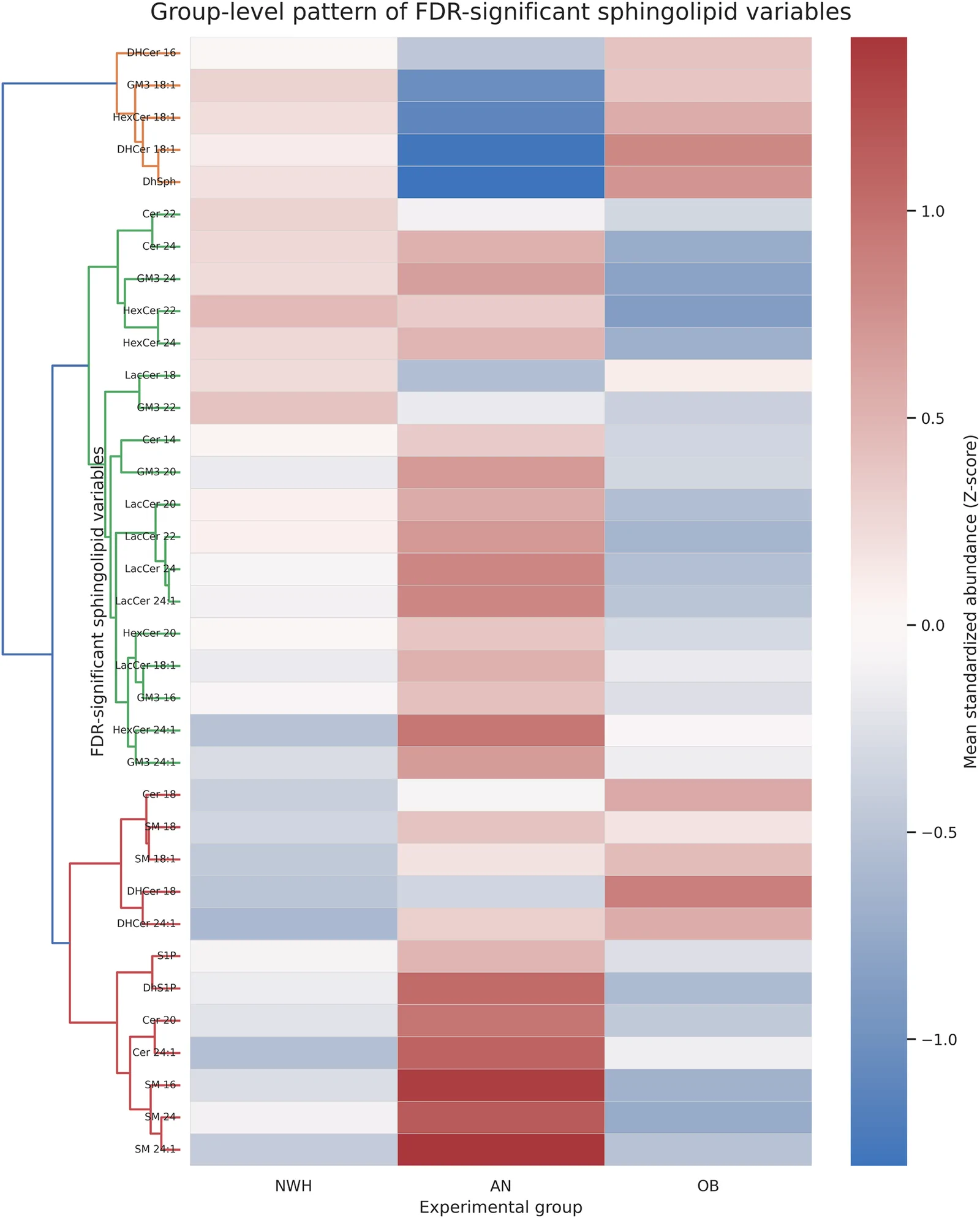
Group-level heatmap of clustered sphingolipid patterns. Group-level heatmap summarizing the mean standardized abundance of the FDR-significant sphingolipid variables in NWH, AN, and OB. Variables are ordered according to the feature dendrogram obtained from hierarchical clustering of the individual-level data. Values are expressed as mean Z-scores for each group and analyte. Positive values indicate higher-than-average relative abundance, and negative values indicate lower-than-average relative abundance across the full study cohort. The dendrogram on the left indicates the similarity among sphingolipid variables based on their standardized abundance pattern across participants. The heatmap complements the individual-level clustering by showing the direction of group-level shifts, making it easier to identify analyte modules preferentially increased in AN, preferentially increased in OB, or reduced relative to NWH. As in **Figure 8**, the dendrogram lines represent hierarchical fusion distances, not inferential p-values. The figure should therefore be interpreted as an exploratory, unsupervised visualization of multivariate lipid architecture rather than a formal classification model.

The three principal sample clusters derived from the dendrogram were compositionally related to group membership (cluster 1: n=39, 11 NWH, 28 AN, 0 OB; predominant group AN (71.8%); cluster 2: n=43, 24 NWH, 0 AN, 19 OB; predominant group NWH (55.8%); cluster 3: n=32, 14 NWH, 0 AN, 18 OB; predominant group OB (56.2%)) (**Figure 10**). This distribution indicates that the FDR-significant sphingolipid signature carries phenotypic information sufficient to partially segregate NWH, AN, and OB subjects in an unsupervised manner. Several participants clustered outside the branch dominated by their own clinical group, supporting inter-individual lipidomic heterogeneity within each phenotype.

**Figure 10:**
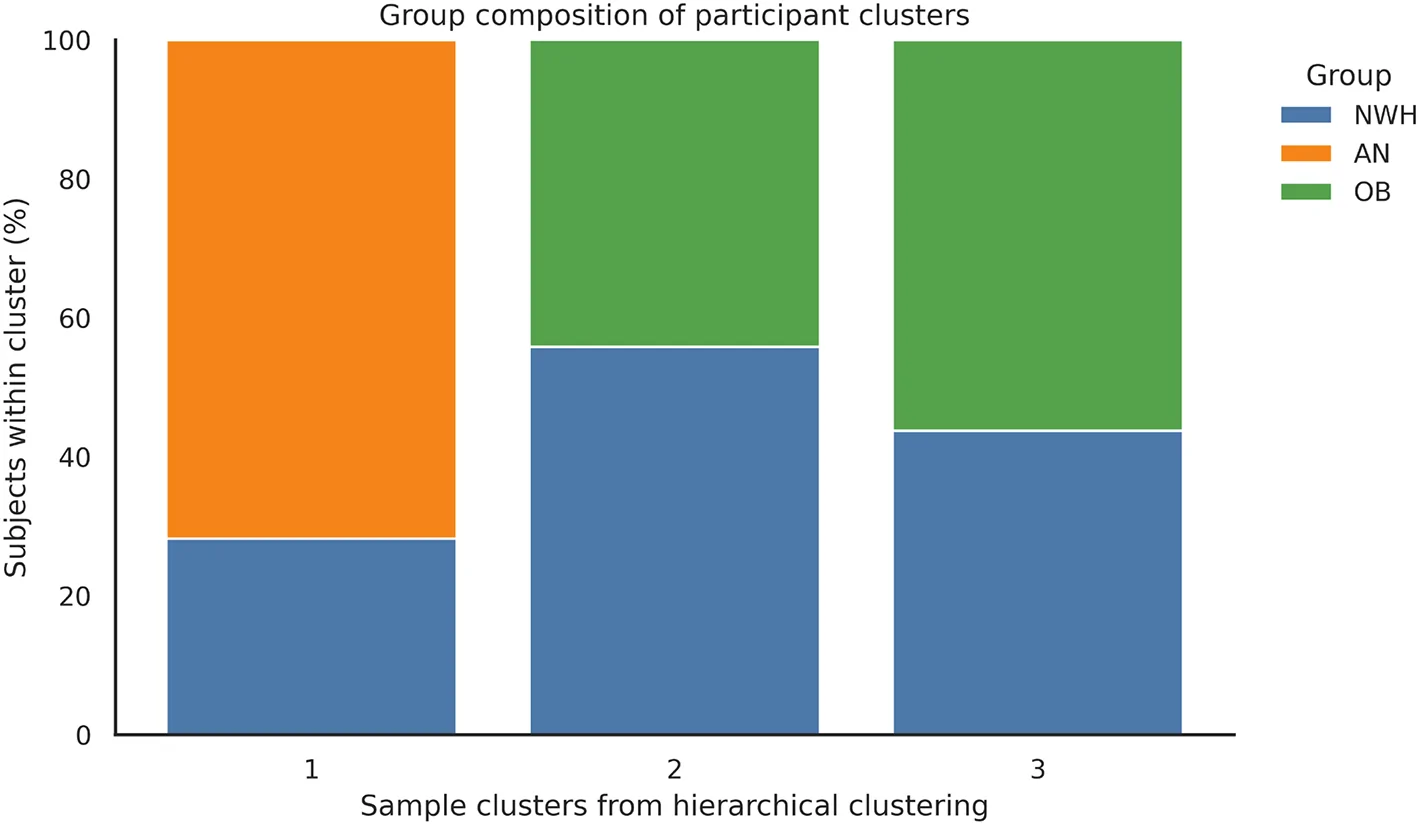
Group composition of participant clusters. Stacked bar plot showing the percentage composition of the three participant clusters obtained by cutting the sample dendrogram into three major branches. Each bar represents one unsupervised cluster, and colored segments indicate the proportion of NWH, AN, and OB participants within that cluster. The figure provides a quantitative summary of how the unsupervised sample dendrogram relates to the experimental groups. Cluster composition was as follows: cluster 1: n=39, 11 NWH, 28 AN, 0 OB; predominant group AN (71.8%); cluster 2: n=43, 24 NWH, 0 AN, 19 OB; predominant group NWH (55.8%); cluster 3: n=32, 14 NWH, 0 AN, 18 OB; predominant group OB (56.2%). The association between sample clusters and experimental groups was evaluated using a contingency table approach (chi-square test: p=5.78e-16), used descriptively to quantify the extent to which unsupervised lipid-based clustering recapitulated group structure. This analysis does not replace formal differential testing but helps interpret whether the multivariate lipid pattern is phenotypically organized.

Feature clustering identified analyte modules with coherent abundance patterns across participants (**Figures 8**, **9**). The variables contributing most strongly to between-group heterogeneity at the group-mean standardized level included SM 24:1, SM 16, DHCer 18:1, DhSph, SM 24, HexCer 18:1, Cer 24:1, DhS1P, among others (**Figure 9**). The group-level heatmap highlighted two major biological axes: an AN-associated axis characterized by relative increases in several sphingomyelin, lactosylceramide, and GM3-related variables, and an OB-associated axis characterized by elevations in selected *de novo* ceramide pathway-related measures (**Figure 9**). These patterns are consistent with a multivariate sphingolipid remodeling that differs between undernutrition-associated and obesity-associated phenotypes (i.e., AN and OB, respectively).

Taken together, the hierarchical clustering analysis supports the presence of a structured sphingolipidomic signature across NWH, AN, and OB. By using only FDR-significant variables while retaining all selected analytes in an unsupervised framework, the analysis integrates the univariate evidence into a multivariate representation of lipid architecture. The resulting heatmaps suggest that AN and OB are not simply opposite deviations from NWH, but are associated with partially distinct sphingolipid modules, involving differential contributions of sphingomyelin, glycosphingolipid, dihydroceramide, and sphingoid-base pathways (**Figures 8**-**10**).

#### 3.4.2 PLS-DA and VIP analysis of FDR-significant sphingolipid variables

The PLS-DA model based on the FDR-significant sphingolipid subset showed a structured distribution of participants across the first two latent variables (**Figure 11**). The first latent variable accounted for approximately 37.8% of the standardized sphingolipid variance captured in the X matrix, whereas the second latent variable accounted for approximately 11.1%. The score plot showed that AN and OB subjects tended to occupy different regions of the latent-variable space. NWH subjects were positioned closer to the central/intermediate portion of the plot, consistent with a reference-like lipidomic configuration rather than with a strongly polarized disease-associated profile.

**Figure 11:**
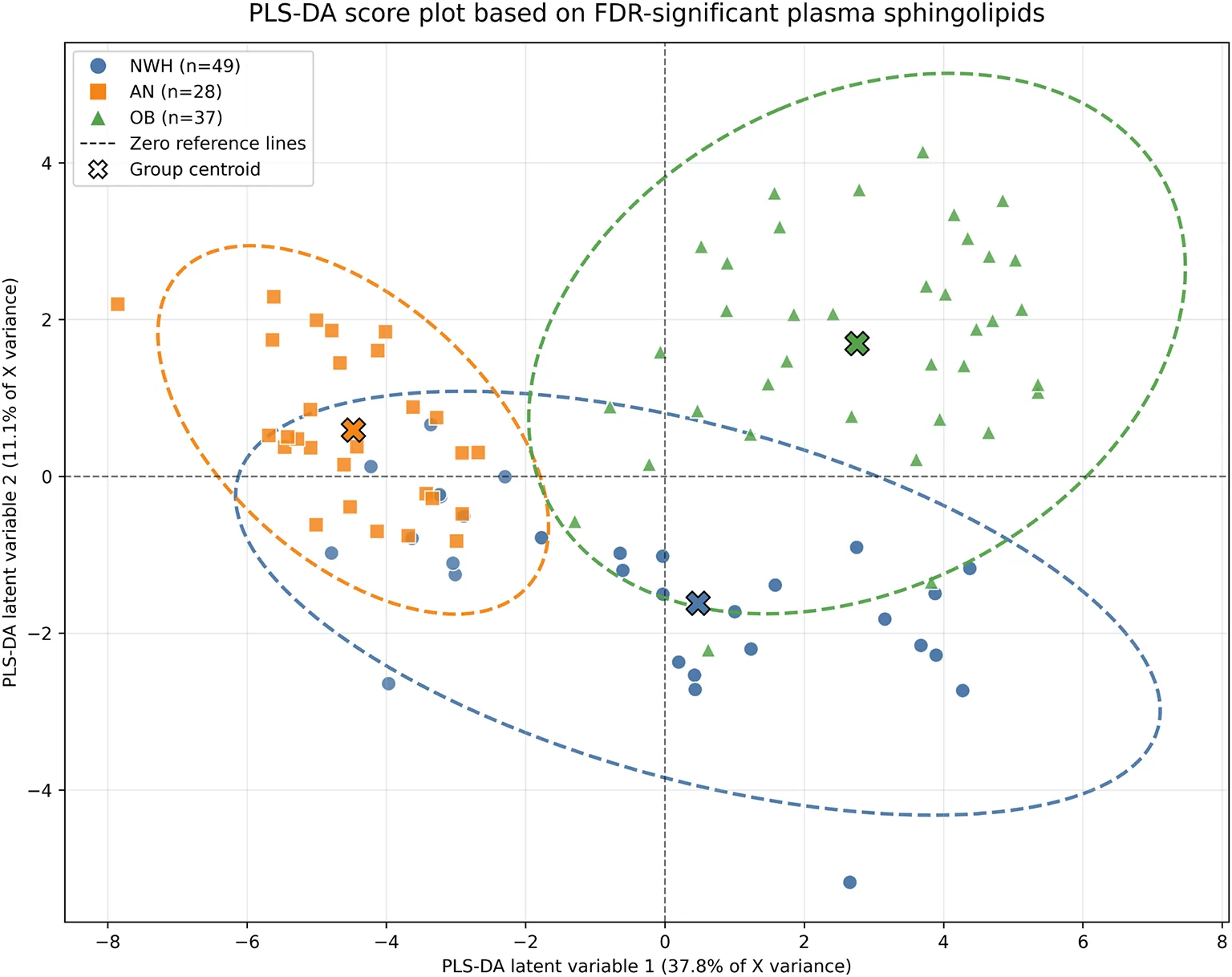
PLS-DA score plot based on FDR-significant sphingolipid variables. The score plot displays individual participants projected onto the first two PLS-DA latent variables derived from the FDR-significant sphingolipid matrix. Each point represents one participant and is colored according to experimental group: NWH, AN, or OB. The black X symbols indicate group centroids. The dashed vertical and horizontal lines mark the zero values of latent variable 1 and latent variable 2, respectively. Ellipses represent 95% confidence regions around the distribution of each group in the latent-variable space. The zero reference lines are included to facilitate interpretation of the directionality of sample projection along the two supervised axes; they are not statistical thresholds. The ellipses summarize within-group dispersion and overlap, therefore providing a visual assessment of group separation and heterogeneity. Because PLS-DA is supervised, group labels were used during model fitting; the plot should therefore be interpreted as a discrimination-oriented representation rather than an unsupervised map of the data.

The separation was not absolute, as expected for an exploratory biological dataset and is also consistent with the partially overlapping metabolic nature of nutritional and adiposity-related phenotypes. Nevertheless, the distribution of group centroids and the orientation of the confidence ellipses indicated that the supervised model captured phenotype-related information embedded in the FDR-significant sphingolipid panel (**Figure 11**).

The VIP analysis identified 15 variables with VIP values ≥ 1.0, indicating above-average contribution to the multigroup PLS-DA solution (**Figure 12**). The highest-ranking analytes were DHCer 24:1, DHCer 18, HexCer 22, Cer 24:1, HexCer 24:1, SM 24:1, Cer 18, SM 18:1, GM3 24, SM 16, DHCer 18:1, DhSph. This ranking suggests that a single sphingolipid family did not carry the discriminant signal, but rather a coordinated pattern involving sphingomyelin species, ceramide-related indices, dihydroceramides, glycosphingolipids, and sphingoid-base measures, several of which also contributed prominently to the PCA loading structure (**Figure 6**). Therefore, the supervised structure appears to reflect pathway-level remodeling rather than isolated analyte-level perturbations.

**Figure 12:**
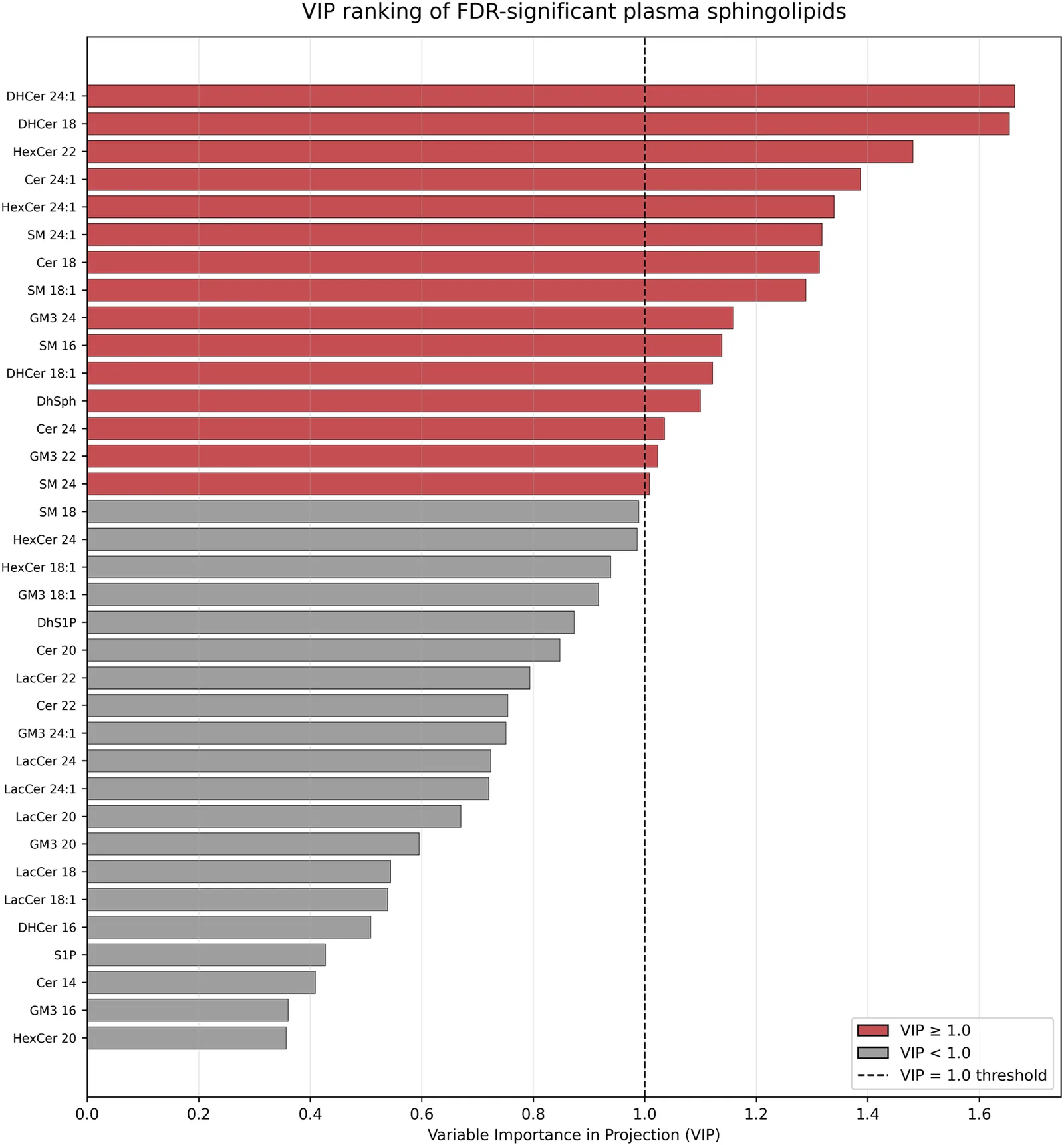
Variable Importance in Projection (VIP) ranking. Horizontal bars show VIP scores for all FDR-significant sphingolipid variables included in the PLS-DA model. Variables are ordered from highest to lowest VIP value. Red bars identify variables with VIP >= 1.0, whereas grey bars indicate variables with VIP < 1.0. The dashed vertical line marks the conventional VIP = 1.0 threshold. The dashed VIP = 1.0 line denotes the average expected contribution of a variable to the multicomponent PLS-DA model. Variables above this threshold are considered to contribute more than average to class separation. VIP scores quantify multivariate importance, not univariate statistical significance; in this analysis, all variables entered into the model were already selected because they were significant after FDR correction in the overall group comparison.

The class-coefficient heatmap for the top VIP-ranked variables provided additional insight into the directionality of the supervised discrimination (**Figure 13**). Variables with relatively stronger positive coefficients for AN included SM 24:1, SM 16, Cer 24:1, HexCer 24:1, SM 24, Cer 20, whereas OB-associated coefficients were more prominent for DHCer 18, DHCer 24:1, Cer 18, SM 18:1, DHCer 18:1, SM 18 (**Figure 13**). NWH-related coefficients were comparatively less extreme, supporting the interpretation that the two pathological phenotypes diverge from the normal-weight reference along partly distinct sphingolipid axes. This pattern is consistent with the observation that AN and OB are not merely opposite quantitative deviations in body weight, but are associated with qualitatively different sphingolipid configurations.

**Figure 13:**
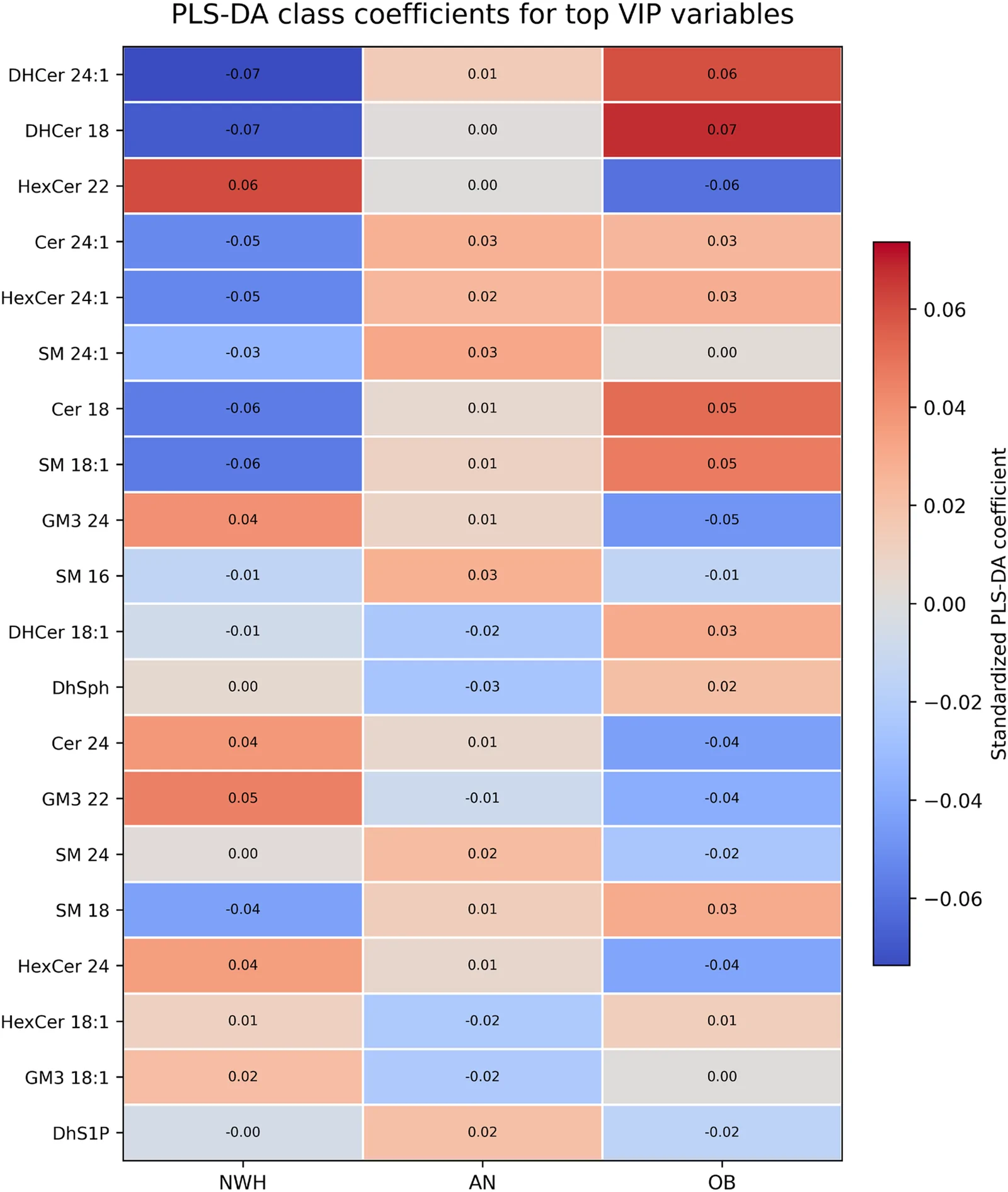
PLS-DA class-coefficient heatmap for the top VIP variables. The heatmap shows standardized PLS-DA coefficients for the top VIP-ranked sphingolipid variables across the three response classes: NWH, AN, and OB. Warmer tones indicate positive coefficients for a given class, whereas cooler tones indicate negative coefficients. The thin white grid lines separate variables and class columns to improve readability. Coefficients provide directional information complementary to VIP scores. A high VIP score indicates that a variable contributes strongly to discrimination, whereas the class-specific coefficient pattern suggests which group response is most associated with that variable within the fitted model. Coefficients should not be interpreted as causal effects or as independent biomarkers without external validation.

In cross-validation, the two-component model achieved an overall accuracy of 0.851, a balanced accuracy of 0.854, and an exploratory Q2Y of 0.552. These values suggest that the selected FDR-significant sphingolipid profile contains group-related discriminatory information.

Taken together, the PLS-DA and VIP analyses support the presence of a multivariate sphingolipidomic signature differentiating NWH, AN, and OB. The model integrates the FDR-significant univariate evidence into a supervised latent-variable framework and highlights the analytes that most strongly contribute to between-group discrimination (**Figures 11**-**13**). The resulting pattern reinforces the concept that undernutrition-associated and obesity-associated phenotypes involve distinct rearrangements of plasma sphingolipid metabolism, with differential contributions from sphingomyelin, ceramide, dihydroceramide, glycosphingolipid, and sphingoid-base pathways.

### 3.5 Age-adjusted associations between FDR-significant plasma sphingolipids and selected clinical/metabolic predictors

#### 3.5.1 Total of significant associations

Across the 266 age-adjusted sphingolipid-clinical association models, 141 associations remained statistically significant after global Benjamini-Hochberg FDR correction, involving 34 distinct sphingolipid variables. Overall, the strongest and most consistent association pattern was observed for selected cardiometabolic predictors, indicating that the FDR-significant sphingolipidomic signature is quantitatively related to clinically relevant cardiometabolic gradients after accounting for age.

The complete list of associations is reported in **Supplementary Table 1**.

#### 3.5.2 BMI as predictor

After FDR correction, 30 significant associations emerged, including 12 positive and 18 inverse relationships. The strongest associations were observed for DHCer 18, SM 24, CER18/24, SM 16, and DHCer 18:1. In particular, higher BMI values were positively associated with DHCer 18 (β=0.0006, standardized β=0.6535, p-FDR ≤ 0.0001) and CER18/24 (β=0.0016, standardized β=0.6029, p-FDR ≤ 0.0001), whereas inverse relationships were detected for SM 24 (β=-1.4742, standardized β=-0.5969, p-FDR ≤ 0.0001) and SM 16 (β=-1.9511, standardized β=-0.5680, p-FDR ≤ 0.0001). Overall, the BMI-related sphingolipid profile was characterized by prominent associations involving dihydroceramides and ceramide ratios, together with an inverse relationship with several sphingomyelin species.

#### 3.5.3 HOMA-IR as predictor

Eighteen associations remained significant after FDR correction, of which 12 were positive and 6 inverse. The most prominent relationships involved DHCer 18, DHCer 24:1, DHCer 18:1, Cer 18, and GM3 18:1. Notably, HOMA-IR showed strong positive associations with DHCer 18 (β=0.0027, standardized β=0.6847, p-FDR ≤ 0.0001), DHCer 24:1 (β=0.0158, standardized β=0.5095, p-FDR ≤ 0.0001), DHCer 18:1 (β=0.0011, standardized β=0.4429, p-FDR ≤ 0.0001), and Cer 18 (β=0.0083, standardized β=0.4420, p-FDR ≤ 0.0001). These findings indicate a marked association between insulin resistance and alterations in both ceramide and dihydroceramide metabolism.

#### 3.5.4 DBP as Predictor

Sixteen significant associations persisted after FDR correction, including 11 positive and 5 inverse associations. The strongest relationships were observed for Cer 18, DHCer 18, DHCer 18:1, SM 18:1, and SM 24. Among these, DBP was positively associated with Cer 18 (β=0.0023, standardized β=0.4592, p-FDR ≤ 0.0001), DHCer 18 (β=0.0005, standardized β=0.4392, p-FDR ≤ 0.0001), DHCer 18:1 (β=0.0002, standardized β=0.3818, p-FDR=0.0001), and SM 18:1 (β=0.2859, standardized β=0.3856, p-FDR=0.0002). Collectively, these results support a close relationship between DBP and multiple sphingolipid subclasses, particularly ceramides and sphingomyelins.

#### 3.5.5 SBP as predictor

Twenty-six associations remained significant after FDR correction, comprising 8 positive and 18 inverse associations. The most relevant associations involved SM 24:1, SM 24, SM 16, DhSph, and DHCer 18:1. Inverse relationships predominated, particularly for SM 24:1 (β=-2.3610, standardized β=-0.5766, p-FDR ≤ 0.0001), SM 24 (β=-1.1333, standardized β=-0.5337, p-FDR ≤ 0.0001), and SM 16 (β=-1.5605, standardized β=-0.5284, p-FDR ≤ 0.0001). By contrast, DhSph displayed a positive association with SBP (β=0.0005, standardized β=0.4923, p-FDR ≤ 0.0001). Overall, SBP was associated with a broad sphingolipid remodeling pattern, characterized mainly by inverse associations with sphingomyelin species.

#### 3.5.6 CRP as predictor

Eighteen associations survived FDR correction, including 10 positive and 8 inverse relationships. The strongest associations involved DHCer 18, DHCer 18:1, CER18/24, Cer 18, and DHCer 24:1. CRP levels were positively associated with DHCer 18 (β=0.0095, standardized β=0.5153, p-FDR ≤ 0.0001), DHCer 18:1 (β=0.0053, standardized β=0.4605, p-FDR ≤ 0.0001), CER18/24 (β=0.0243, standardized β=0.4091, p-FDR ≤ 0.0001), and Cer 18 (β=0.0358, standardized β=0.3963, p-FDR ≤ 0.0001). These data suggest that systemic inflammation is closely linked to alterations in ceramide and dihydroceramide metabolism.

#### 3.5.7 HDL-C as predictor

Twenty-three associations remained significant after FDR correction, with a relatively balanced distribution between positive (n=12) and inverse (n=11) associations. The strongest relationships involved DHCer 18, DHCer 18:1, DhS1P, SM 16, and CER18/24. HDL-C was inversely associated with DHCer 18 (β=-0.0003, standardized β=-0.5609, p-FDR ≤ 0.0001) and DHCer 18:1 (β=-0.0002, standardized β=-0.4685, p-FDR ≤ 0.0001), whereas positive associations were observed for DhS1P (β=0.0148, standardized β=0.4812, p-FDR ≤ 0.0001) and SM 16 (β=1.1168, standardized β=0.4753, p-FDR ≤ 0.0001). Taken together, these findings indicate that HDL-C is associated with a mixed sphingolipid signature involving both protective and potentially detrimental lipid species.

#### 3.5.8 TG as Predictor

Ten associations remained significant after FDR correction, of which 9 were positive and only 1 was inverse. The strongest associations were observed for DHCer 24:1, DHCer 18, Cer 18, SM 18:1, and DHCer 16. In particular, TG levels showed strong positive associations with DHCer 24:1 (β=0.0010, standardized β=0.5819, p-FDR ≤ 0.0001) and DHCer 18 (β=0.0001, standardized β=0.5815, p-FDR ≤ 0.0001), while additional positive relationships were observed for Cer 18 (β=0.0003, standardized β=0.3221, p-FDR=0.0018) and SM 18:1 (β=0.0497, standardized β=0.3122, p-FDR=0.0025). Overall, TG levels were predominantly associated with increased concentrations of ceramide and dihydroceramide species.

## 4 Discussion

The present study provides a comprehensive and methodologically harmonized characterization of plasma sphingolipid signatures across the extremes of energy balance by directly comparing women with AN, OB, and NWH controls within the same analytical framework (26–29). By integrating univariate analyses, exploratory visualization approaches, unsupervised multivariate analyses, hierarchical clustering, supervised discrimination models, and regression analyses, the study demonstrates that AN and OB are associated with profoundly remodeled but biologically distinct sphingolipidomic configurations (30–33). Importantly, the observed remodeling was characterized by coordinated and class-specific changes in individual sphingolipid species and ceramide-ratio indices, rather than by a uniform directional change across the entire sphingolipid panel (34–36).

One of the most important findings of the present study is the robustness of the sphingolipidomic signal after multiple-testing correction (37, 38). After Benjamini-Hochberg FDR adjustment, 38 of 45 sphingolipid variables remained significant, indicating that isolated nominal associations did not drive the lipidomic separation among AN, OB, and NWH but instead reflected a biologically coherent and statistically stable multivariate pattern (37–39). Notably, the variables that survived FDR correction included several of the most biologically informative lipid classes, including ceramides, dihydroceramides, sphingomyelins, glycosphingolipids, sphingoid-base-related metabolites, and ceramide-ratio indices (30–36). The persistence of these associations after correction for multiple comparisons strongly supports the interpretation that the sphingolipidomic remodeling identified in the present study reflects genuine metabolic biology rather than stochastic analytical variability (37–39).

At the global multivariate level, PCA, hierarchical clustering analysis, and PLS-DA consistently demonstrated structured separation among the three nutritional phenotypes (37–41). AN and OB occupied clearly divergent regions of the multivariate lipidomic space, whereas NWH subjects generally exhibited an intermediate and less polarized distribution (26–33). Importantly, the direct AN-versus-OB comparison generated the largest number of FDR-significant variables, indicating extensive lipidomic divergence between the two pathological phenotypes. However, this interpretation is supported not only by the greater number of statistically significant differences, but also by the distinct class-specific sphingolipid remodeling observed in the two groups. Specifically, AN was characterized predominantly by enrichment of sphingomyelins, glycosphingolipids, and sphingoid-base-related metabolites, whereas OB showed enrichment of selected dihydroceramides and ceramide-ratio indices. Together, these complementary quantitative and qualitative findings argue against a simplistic interpretation of AN and OB as merely opposite ends of a linear BMI-related metabolic continuum and instead support the concept that severe caloric restriction and severe caloric excess induce distinct adaptive states within partially interconnected sphingolipid regulatory networks (26–36).

Within the ceramide compartment, the remodeling pattern was strongly dependent on acyl-chain composition (30–36). OB was characterized by enrichment of selected ceramide-ratio indices and dihydroceramide species, particularly DHCer 18, DHCer 18:1, CER16/24, and CER18/24 (30–33). This profile is consistent with activation of *de novo* sphingolipid synthesis pathways, which are known to be stimulated in obesity, insulin resistance, and lipotoxic states (31–36, 42–45). Experimental and translational studies have shown that ceramide accumulation contributes to impaired insulin signaling, mitochondrial dysfunction, inflammation, and metabolic inflexibility (42–46). The present findings are therefore coherent with the established role of ceramide metabolism in obesity-associated cardiometabolic dysfunction (31–36, 42–46).

In contrast, AN showed a markedly different sphingolipidomic architecture (26–30). Several very-long-chain ceramide-related species, including Cer 20, Cer 24:1, and CER24:1/24, together with multiple sphingomyelins and glycosphingolipids, were increased in AN compared with both NWH and OB (26–30). Particularly striking was the AN-centered elevation of SM 16, SM 24, SM 24:1, LacCer 22, LacCer 24, LacCer 24:1, GM3 20, GM3 24, GM3 24:1, S1P, and DhS1P (26–30). This pattern strongly argues against the traditional concept of AN as a metabolically “hypolipidemic” condition characterized simply by substrate depletion (26–29). Instead, the data indicate active and selective metabolic remodeling involving membrane-associated and signaling-active sphingolipid pathways (27–30, 47).

The marked enrichment of sphingomyelins and glycosphingolipids in AN may reflect adaptive changes in membrane organization, lipoprotein metabolism, cellular stress responses, or the composition of membrane microdomains (47–50). Sphingomyelins are major structural membrane lipids involved in lipid-raft organization and receptor signaling, whereas glycosphingolipids participate in immune regulation, cell-cell communication, and membrane stability (47–51). The coordinated increase in these species in AN therefore suggests that severe chronic caloric restriction induces compensatory remodeling processes rather than a passive reduction of lipid pools (26–30, 47). Likewise, the elevation of S1P and DhS1P in AN suggests activation of sphingosine kinase pathways, which are implicated in vascular signaling, stress adaptation, immune regulation, and endothelial biology (52–55).

An additional important observation is that AN and OB were not differentiated by a monotonic shift across all sphingolipid classes (30–36). Instead, the lipidomic remodeling was highly selective and pathway-dependent (30–36, 42–46). Some variables were elevated predominantly in AN, others in OB, whereas additional species were selectively depleted in one condition (26–36). This structured heterogeneity likely explains why unsupervised PCA did not produce absolute separation despite clear centroid displacement among groups (37–41). The observed overlap should therefore not be interpreted as a lack of biological specificity, but rather as evidence that sphingolipid remodeling is multifactorial and distributed across several partially independent biochemical axes (37–41).

Overall, these findings suggest that AN and OB should not be viewed simply as conditions characterized by opposite quantitative changes in circulating sphingolipids, but rather as distinct states of coordinated sphingolipid pathway remodeling. In OB, the predominant enrichment of dihydroceramides, together with increased ceramide ratio indices (particularly CER18/24 and CER24:1/24), is consistent with activation of the *de novo* ceramide biosynthetic pathway, a mechanism previously linked to nutrient excess, saturated fatty acid overload, endoplasmic reticulum stress, insulin resistance, and lipotoxicity. This pattern suggests increased flux through serine palmitoyltransferase, ceramide synthases, and downstream ceramide-generating pathways, ultimately favouring the accumulation of bioactive ceramides associated with cardiometabolic dysfunction. In contrast, AN exhibited a markedly different metabolic configuration characterized by enrichment of sphingomyelins, lactosylceramides, GM3 species, S1P, and DhS1P, accompanied by selective increases in very-long-chain ceramides but without evidence of generalized activation of the *de novo* ceramide pathway. Rather than reflecting lipotoxic remodeling, this profile is compatible with preferential channeling of sphingolipid metabolism toward membrane structural lipids and signaling glycosphingolipids, potentially representing an adaptive response to prolonged energy deprivation aimed at preserving membrane integrity, cellular signaling, and survival pathways. The concomitant increase in phosphorylated sphingoid bases (S1P and DhS1P) further supports a metabolic shift favouring prosurvival and homeostatic signaling rather than ceramide-mediated lipotoxicity. Therefore, although both disorders involve extensive remodeling of the sphingolipid network, OB appears to be dominated by activation of ceramide-generating pathways associated with metabolic stress, whereas AN is characterized by redistribution of sphingolipid metabolism toward membrane-associated and signaling lipid species. These findings support the concept that opposite nutritional states engage distinct regulatory nodes within the sphingolipid metabolic network rather than representing mirror-image alterations of the same biochemical pathway (**Figure 14**).

**Figure 14:**
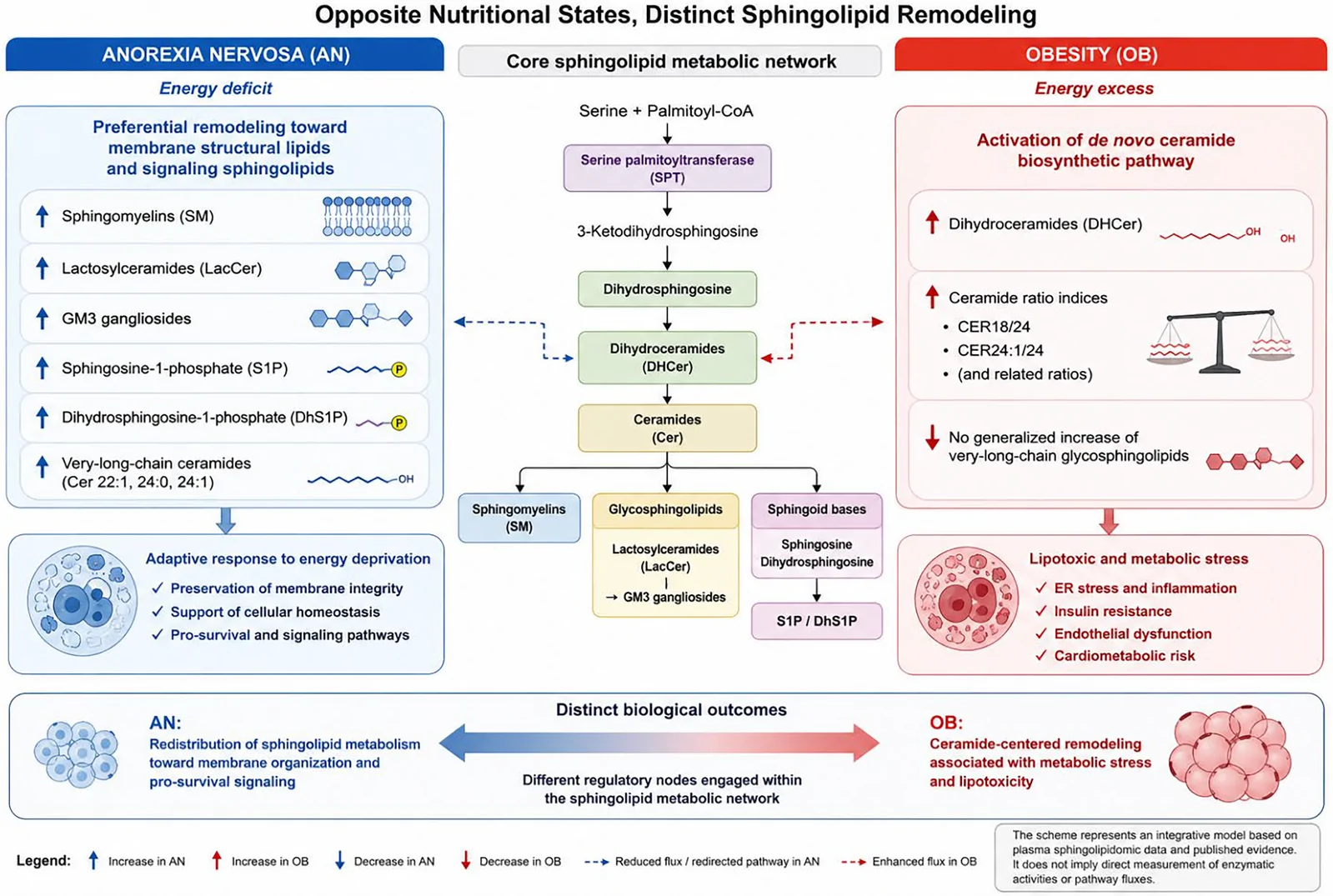
Proposed model of coordinated sphingolipid remodeling across the extremes of energy balance: anorexia nervosa (AN) vs. obesity (OB).

The regression analyses further support the biological relevance of the identified sphingolipid signatures (31–36, 42–46). Multiple sphingolipid variables showed significant associations with BMI, HOMA-IR, TG, HDL-C, CRP, and blood pressure parameters (31–36). Particularly robust associations were observed for DHCer species, ceramide-ratio indices, sphingomyelins, and selected glycosphingolipids (30–36). These findings are highly consistent with previous evidence linking ceramide metabolism to insulin resistance, adipose tissue dysfunction, inflammation, and cardiovascular risk (31–36, 42–46). Importantly, however, the present study extends these concepts to AN, suggesting that sphingolipid dysregulation may also represent a biologically relevant component of severe undernutrition (26–30).

The observation that both AN and OB converge on sphingolipid pathways associated with cardiometabolic regulation is particularly intriguing (31–36, 42–46). Although the two disorders arise from opposite energetic states, both are associated with increased long-term cardiovascular risk (56–59). OB is characterized by classical cardiometabolic dysfunction, including insulin resistance, systemic inflammation, hypertension, and dyslipidemia (31–36, 42–46). AN, in contrast, has traditionally been viewed as metabolically distinct from OB; however, accumulating evidence suggests that severe undernutrition is also associated with cardiovascular abnormalities, endothelial dysfunction, altered autonomic regulation, and persistent metabolic perturbations (56–60). The present findings raise the possibility that sphingolipid remodeling may represent one mechanistic interface linking these apparently opposite conditions to shared downstream cardiometabolic stress pathways (42–46, 52–55).

From a translational perspective, the ceramide ratio indices deserve particular attention (31–36, 42–46). CER18/24 and CER24:1/24 showed some of the largest effect sizes in the entire dataset and strongly contributed to group discrimination (30–36). Ceramide-ratio indices have emerged in recent years as clinically relevant biomarkers of cardiovascular and metabolic risk because they capture relative remodeling of ceramide acyl-chain composition rather than absolute lipid abundance alone (43–46, 61–63). The strong discrimination achieved by these indices in the present study suggests that chain-length remodeling may represent a particularly sensitive marker of nutritional-state-dependent metabolic adaptation (43–46, 61–63).

## 5 Strengths and limitations of the study

The present study also has several methodological strengths. First, AN and OB were investigated within the same study design, analytical workflow, and LC–MS/MS platform, thereby minimizing the substantial methodological heterogeneity that complicates indirect comparisons across independent lipidomic studies. Second, the inclusion of a shared NWH reference group allowed interpretation of both disorder-specific and partially overlapping lipidomic signatures. Third, the integration of univariate, exploratory, clustering, supervised, and regression-based analyses provided convergent evidence supporting the robustness of the observed sphingolipidomic architecture. Fourth, the maintenance of most biologically relevant findings after FDR correction further strengthens the statistical reliability of the results.

Several limitations should nevertheless be acknowledged. The cross-sectional design precludes causal inference regarding the temporal relationship between sphingolipid remodeling and disease development. The sample size, although adequate for detecting robust global effects, may limit the power to detect smaller pairwise differences after correction for multiple testing. Because the study was restricted to women, the findings may not be directly generalizable to male populations. In addition, although the present study identifies strong associations between sphingolipid variables and cardiometabolic parameters, it does not directly assess enzymatic activity, metabolic flux, tissue-specific sphingolipid metabolism, or mechanistic signaling pathways. Future studies integrating lipidomics with transcriptomics, proteomics, stable-isotope metabolic tracing, or functional experimental models will therefore be required to clarify better the biological mechanisms underlying the observed lipidomic remodeling.

There are two additional minor limitations.

Another limitation is that body composition was assessed by standardized bioelectrical impedance analysis rather than dual-energy X-ray absorptiometry (DEXA), which is considered the reference method for body composition assessment. Although BIA is a validated and widely used clinical technique, particularly in patients with AN and OB, its estimates may be less accurate than those obtained with DEXA, especially in the presence of marked alterations in hydration status or body composition.

Finally, educational attainment and socioeconomic status were not formally collected and therefore their potential contribution as residual confounders cannot be completely excluded.

## 6 Conclusion

The present study demonstrates that AN and OB are characterized by profoundly remodeled but biologically distinct plasma sphingolipid signatures. OB was predominantly associated with enrichment of selected dihydroceramides and ceramide-ratio indices consistent with enhanced *de novo* ceramide synthesis and metabolic dysfunction, whereas AN showed a striking enrichment of sphingomyelins, glycosphingolipids, and sphingoid-base-related metabolites consistent with complex adaptive membrane and signaling remodeling. Importantly, most major lipidomic findings remained significant after FDR correction, indicating that the observed signatures reflect robust and biologically coherent metabolic alterations. Although AN and OB represent opposite energetic states, both conditions converge on sphingolipid pathways associated with cardiometabolic regulation and metabolic stress. These findings support the concept that sphingolipids may serve as biologically informative biomarkers and potential mechanistic mediators of metabolic remodeling across the extremes of energy balance.

## Data Availability

The datasets presented in this study can be found in online repositories. The names of the repository/repositories and accession number(s) can be found below: www.zenodo.org.

## Glossary

AN: anorexia nervosa
ANOVA: analysis of variance
BI: body image
BIA: bioelectrical impedance analysis
BHT: butylated hydroxytoluene
BMI: body mass index
BWRP: body weight reduction program
Cer: ceramides
CI: confidence interval
CRP: C-reactive protein
CV: coefficient of variation
DBP: diastolic blood pressure
DEXA: dual-energy X-ray absorptiometry
DHCer: dihydroceramides
DhS1P: dihydrosphingosine-1-phosphate
DhSph: dihydrosphingosine
DSM-5: Diagnostic and Statistical Manual of Mental Disorders, Fifth Edition
EC: Ethical Committee
FDR: false discovery rate
FFM: fat-free mass
FM: fat mass
GM3: monosialodihexosylganglioside 3
HCA: hierarchical clustering analysis
HDL-C: high-density lipoprotein cholesterol
HexCer: hexosylceramides
HOMA-IR: homeostatic model assessment of insulin resistance
HPLC: high-performance liquid chromatography
HR: heart rate
IDF: International Diabetes Federation
IFCC: International Federation of Clinical Chemistry
IQR: interquartile range
IRCCS: Istituto di Ricovero e Cura a Carattere Scientifico
LacCer: lactosylceramides
LC–MS/MS: liquid chromatography–tandem mass spectrometry
LDL-C: low-density lipoprotein cholesterol
LOD: limit of detection
LV: latent variable
MAR: missing at random
mREE: measured resting energy expenditure
NWH: normal-weight healthy
OB: obesity
OLS: ordinary least squares
PCA: principal component analysis
PLS-DA: partial least squares discriminant analysis
Q²: cross-validated predictive ability
QC: quality control
R²: coefficient of determination
REE: resting energy expenditure
S1P: sphingosine-1-phosphate
SBP: systolic blood pressure
SCID-5-CV: Structured Clinical Interview for DSM-5 Disorders, Clinical Version
SE: standard error
SM: sphingomyelins
Std. β: standardized beta coefficient
T-C: total cholesterol
TG: triglycerides
VIP: variable importance in projection
WC: waist circumference.
