# Supplementary material for: Sphingolipid signatures at the extremes of energy balance: a comparative study of obesity and anorexia nervosa in women
Source: Front Endocrinol (Lausanne). 2026 Oct 8;17:1890712. doi: 10.3389/fendo.2026.1890712 (PMC13688175; doi:10.3389/fendo.2026.1890712)
Supplement: Supplementary file 1 [file DataSheet1.pdf]

Supplementary Figure S1. Sequential rationale and complementary roles of the integrated statistical analyses performed in the present study

A complementary analytical workflow to explore, validate and interpret sphingolipidomic differences among Anorexia Nervosa (AN), Normal-Weight Healthy (NWH) and Obesity (OB) groups.

Dataset
114 women
45 sphingolipid variables
(targeted LC–MS/MS)

1 PCA (*Unsupervised*)

Statistical question

Does the overall sphingolipidomic structure naturally separate the study groups without using prior class labels?

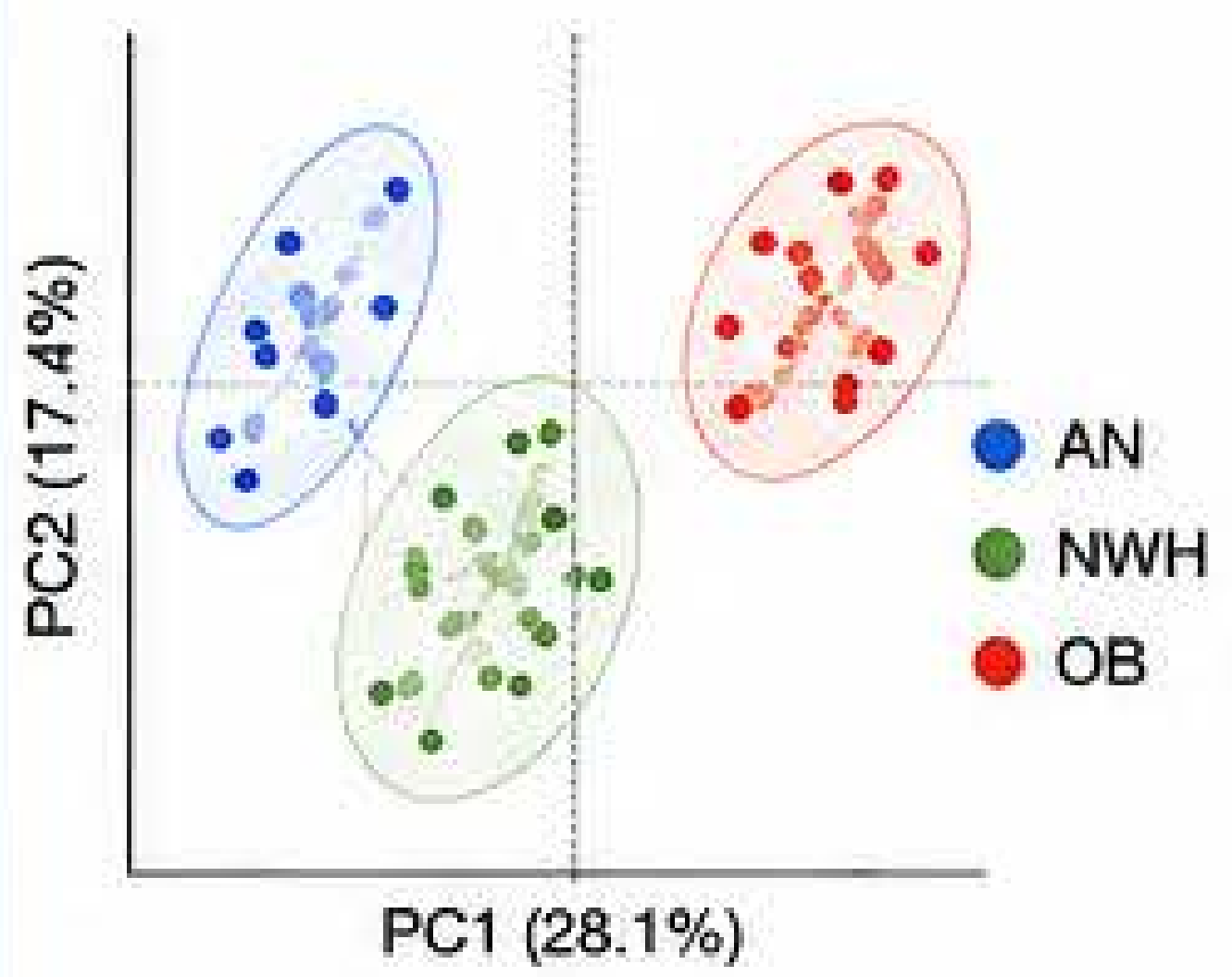

Specific contribution

- Global exploration of covariance structure
- Identification of dominant sources of biological variability
- Unbissed visualization of intrinsic sample clustering

Output

Global metabolic organization of the dataset

2 Univariate statistics + FDR correction

Statistical question

Which sphingolipid species differ significantly among AN, NWH and OB?

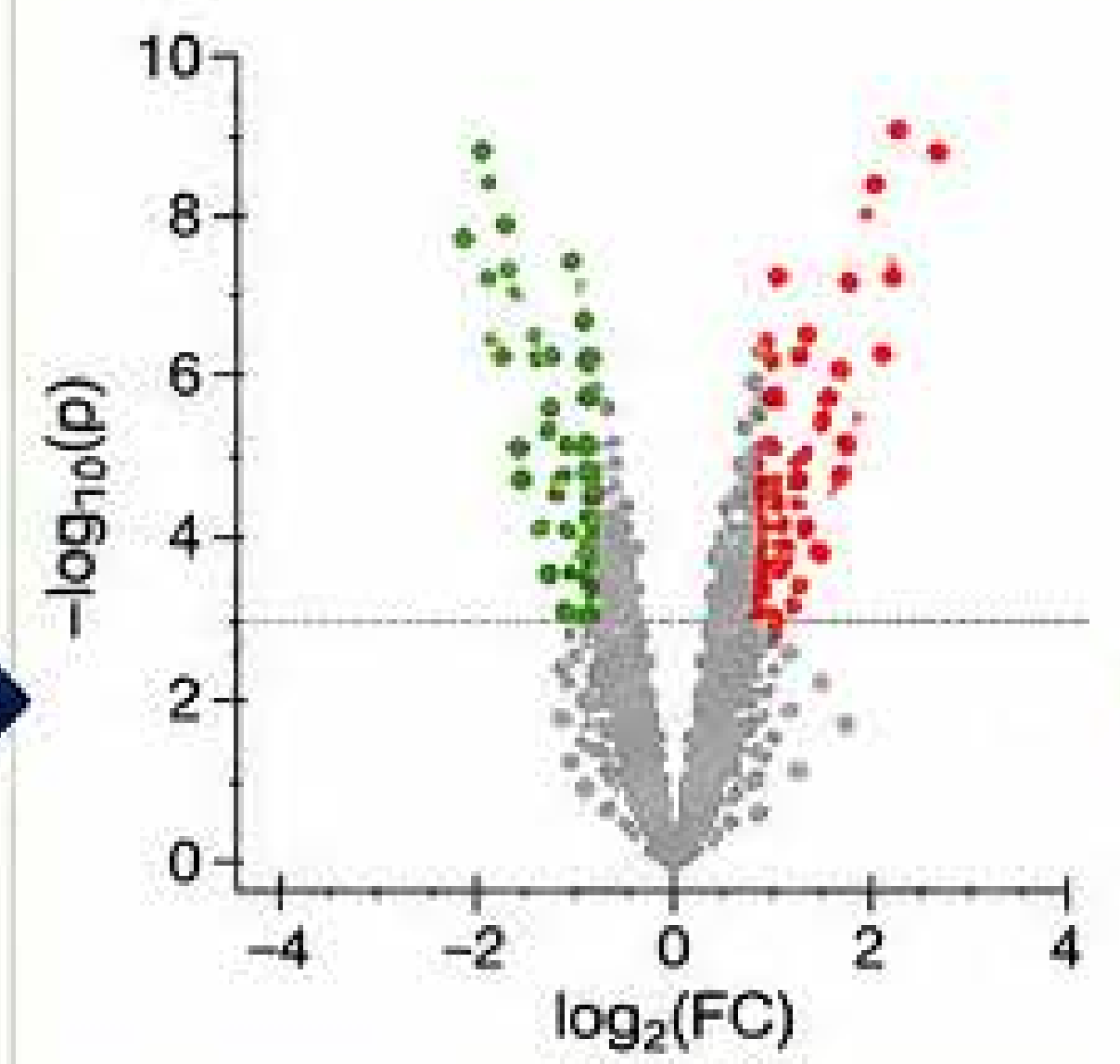

Specific contribution

- Identification of sphingolipids with statistically significant differences
- Control of false discoveries (FDR)
- Selection of biologically informative variables for subsequent analyses

Output

Selection of biologically informative sphingolipids (n = 38 significant variables)

3 Volcano plots (directed contrasts)

Statistical question

Which sphingolipid species show the strongest increases or decreases with effect size and statistical significance in each pairwise comparison?

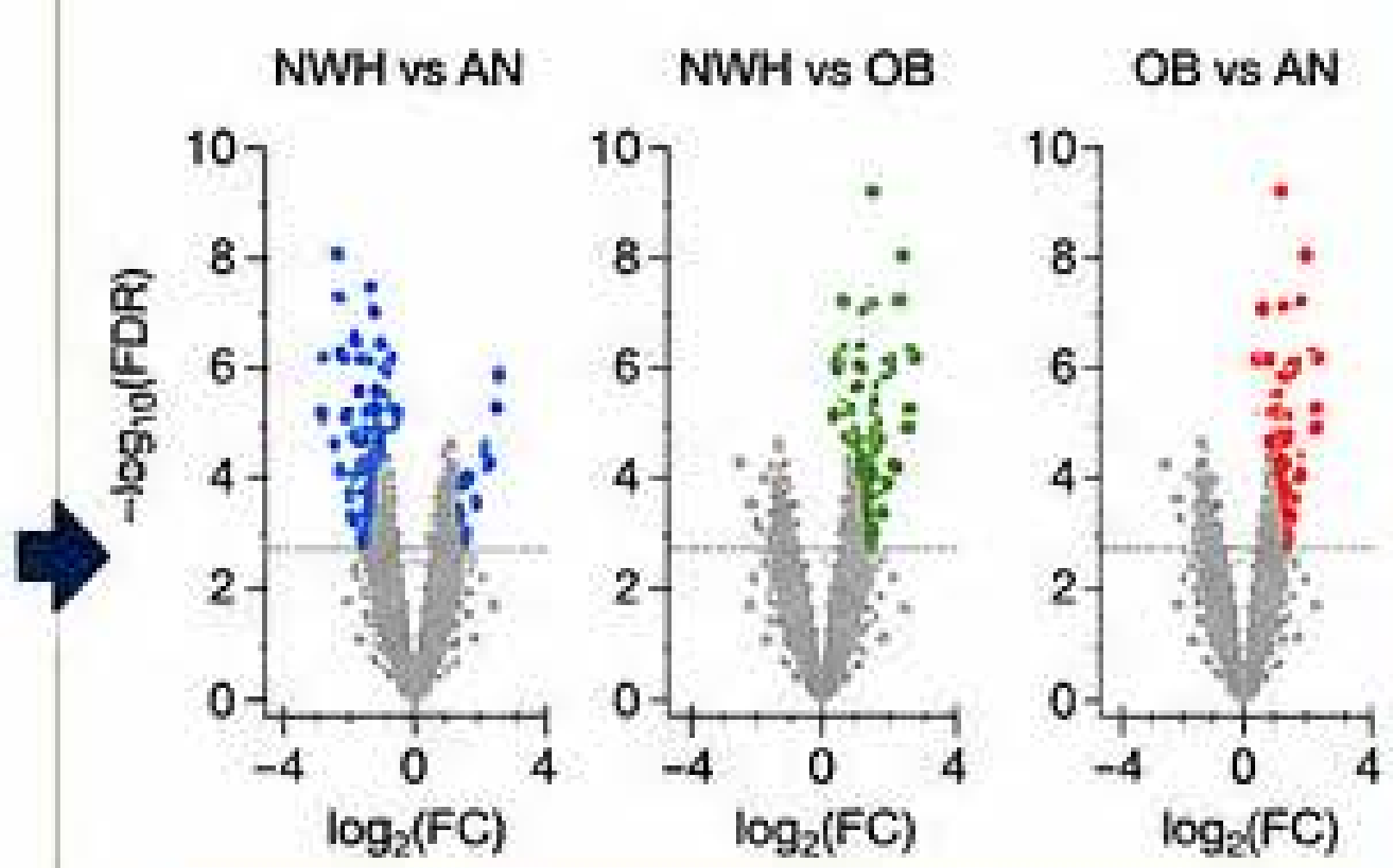

Specific contribution

- Visualization of magnitude (effect size) and significance simultaneously
- Identification of up- and down-regulated species in each pairwise comparison
- Prioritization of biologically relevant lipid species

Output

Directional summary of lipid dysregulation in pairwise contrasts (-- fold-change vs significance landscape)

4 HCA + Heatmap (*unsupervised*)

Statistical question

How are subjects and sphingolipid species hierarchically organized according to similarity?

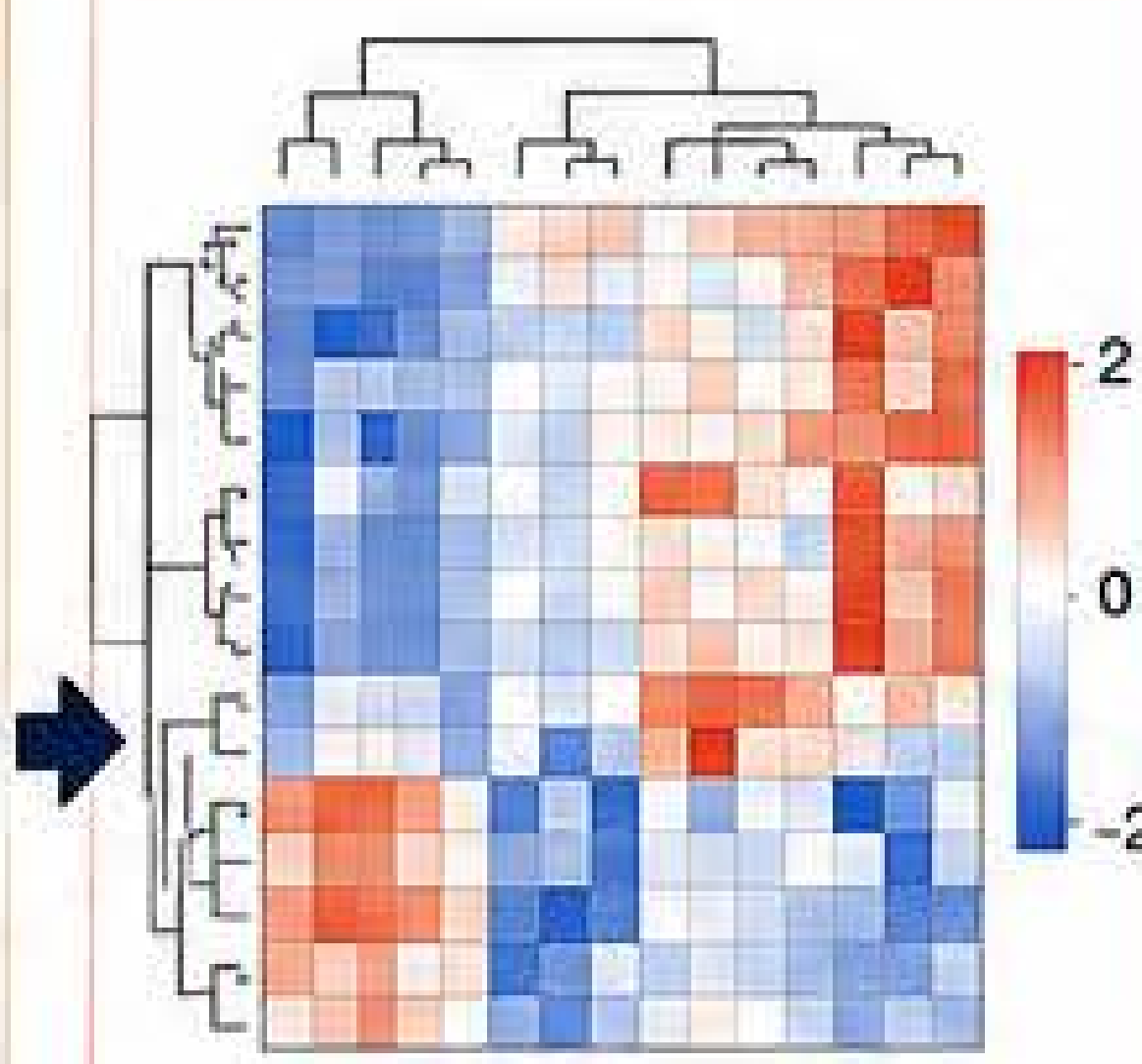

Specific contribution

- Independent validation of clustering
- Visualization of coordinated lipid remodeling
- Identification of sample and lipid clusters

Output

Hierarchical relationships among samples and lipids

5 PLS-DA (*Supervised*)

Statistical question

Can the three nutritional phenotypes be optimally discriminated using multivariate information?

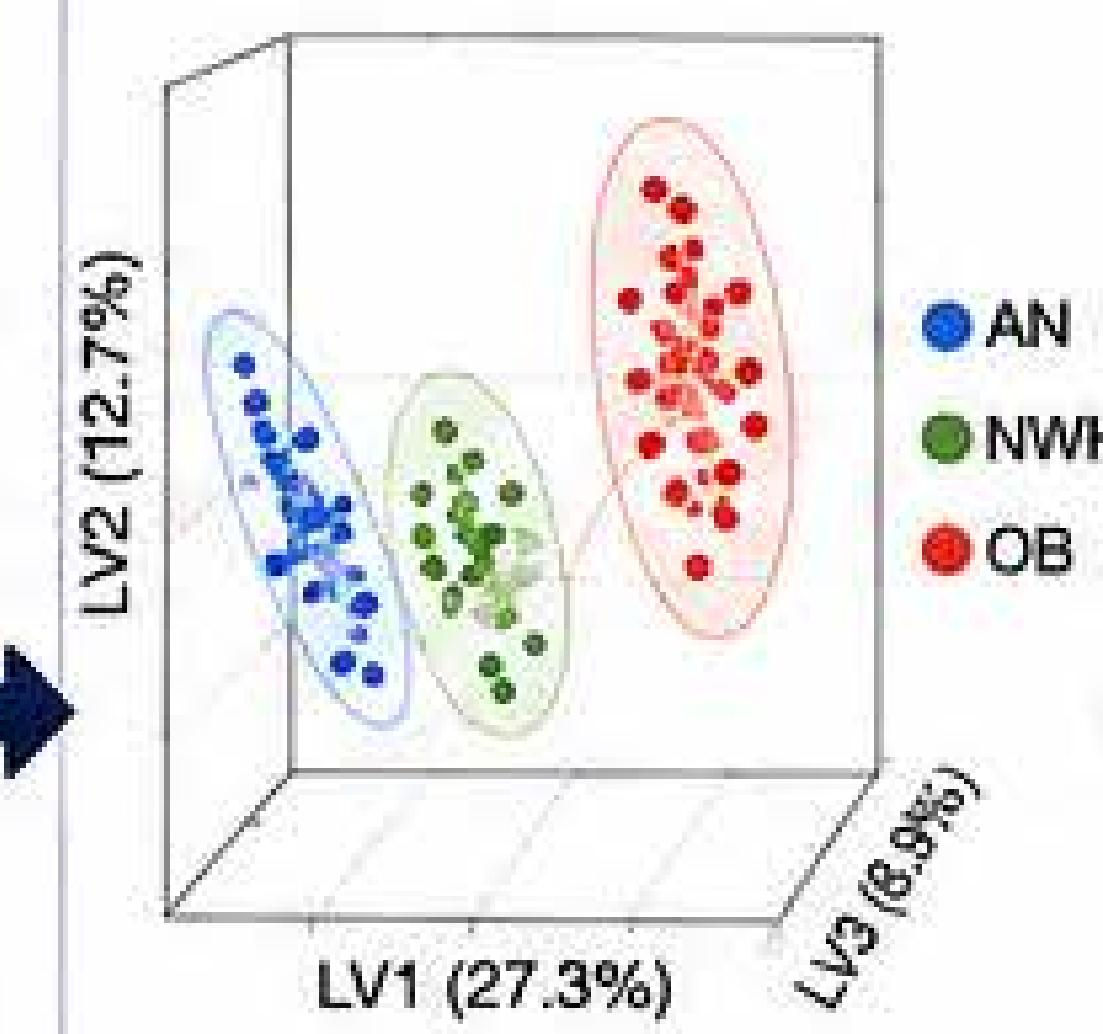

Specific contribution

- Supervised classification
- Assessment of discriminatory performance (accuracy, R^2, Q^2)
- Identification of latent variables maximizing class separation

Output

Robust discrimination among AN, NWH and OB

6 VIP analysis (*from PLS-DA*)

Statistical question

Which sphingolipid species drive the observed group discrimination?

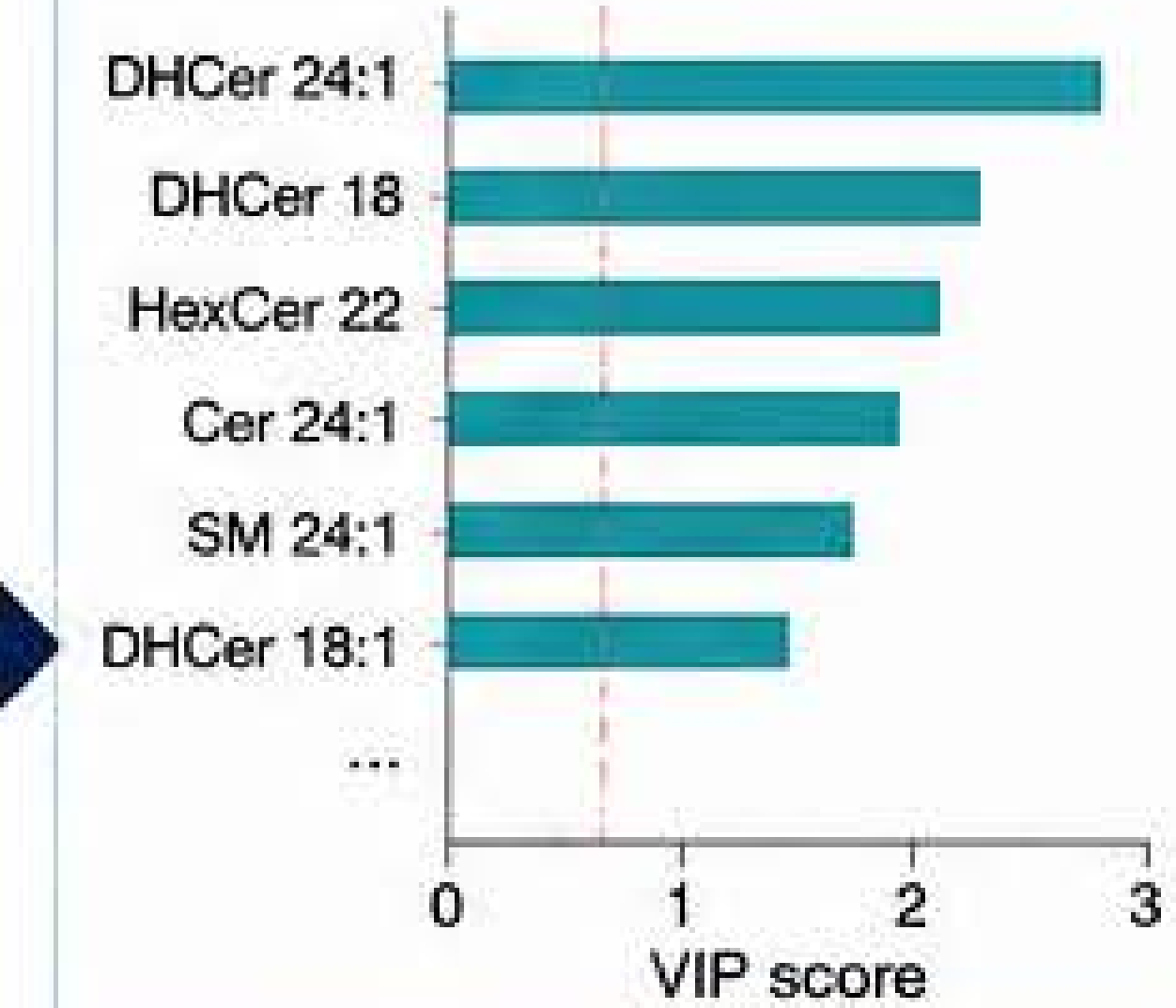

Specific contribution

- Ranking of discriminant sphingolipids
- Identification of the most influential variables
- Biological interpretation and candidate biomarkers

Output

Identification of the most influential sphingolipid species

Integrated biological interpretation

The integrated statistical analyses are intentionally designed as a sequential and complementary workflow. Each method addresses a distinct statistical question and generates unique information that cannot be obtained by the other approaches. The convergence of independent univariate, unsupervised and supervised analyses increases confidence that the observed sphingolipidomic differences represent robust biological signals rather than method-dependent findings.

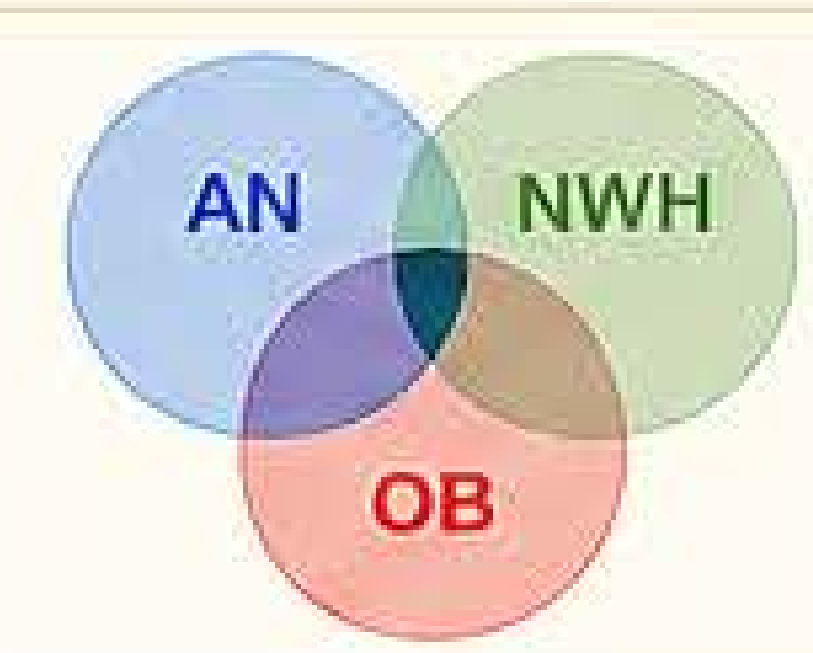

Convergence of methods reinforces the robustness of biological conclusions across the extremes of energy balance.

AN	Anorexia Nervosa	LC–MS/MS	Liquid Chromatography–Tandem Mass Spectrometry	PCA	Principal Component Analysis	PLS-DA	Partial Least Squares Discriminant Analysis	VIP	Variable Importance in Projection
NWH	Normal-Weight Healthy	FDR	False Discovery Rate	HCA	Hierarchical Clustering Analysis			LV	Latent Variable
