# Supplementary material for: Sphingolipid signatures at the extremes of energy balance: a comparative study of obesity and anorexia nervosa in women
Source: Front Endocrinol (Lausanne). 2026 Oct 8;17:1890712. doi: 10.3389/fendo.2026.1890712 (PMC13688175; doi:10.3389/fendo.2026.1890712)
Supplement: Supplementary file 2 [file DataSheet1.docx]

*Kruskal-Wallis rank-sum test: overall p = 0.0171; FDR-adjusted p = 0.0202.*

*Kruskal-Wallis rank-sum test: overall p = <0.0001; FDR-adjusted p = <0.0001.*

*Kruskal-Wallis rank-sum test: overall p = <0.0001; FDR-adjusted p = <0.0001.*

*Kruskal-Wallis rank-sum test: overall p = 0.0061; FDR-adjusted p = 0.0086.*

*Kruskal-Wallis rank-sum test: overall p = <0.0001; FDR-adjusted p = <0.0001.*

*Kruskal-Wallis rank-sum test: overall p = <0.0001; FDR-adjusted p = <0.0001.*

*Kruskal-Wallis rank-sum test: overall p = 0.0006; FDR-adjusted p = 0.0009.*

*Kruskal-Wallis rank-sum test: overall p = <0.0001; FDR-adjusted p = <0.0001.*

*Kruskal-Wallis rank-sum test: overall p = <0.0001; FDR-adjusted p = <0.0001.*

*Kruskal-Wallis rank-sum test: overall p = <0.0001; FDR-adjusted p = <0.0001.*

*Kruskal-Wallis rank-sum test: overall p = <0.0001; FDR-adjusted p = <0.0001.*

*Kruskal-Wallis rank-sum test: overall p = 0.0118; FDR-adjusted p = 0.0152.*

*Kruskal-Wallis rank-sum test: overall p = <0.0001; FDR-adjusted p = 0.0001.*

*Kruskal-Wallis rank-sum test: overall p = <0.0001; FDR-adjusted p = <0.0001.*

*Kruskal-Wallis rank-sum test: overall p = <0.0001; FDR-adjusted p = <0.0001.*

*Kruskal-Wallis rank-sum test: overall p = <0.0001; FDR-adjusted p = <0.0001.*

*Kruskal-Wallis rank-sum test: overall p = 0.0089; FDR-adjusted p = 0.0121.*

*Kruskal-Wallis rank-sum test: overall p = <0.0001; FDR-adjusted p = <0.0001.*

*Kruskal-Wallis rank-sum test: overall p = <0.0001; FDR-adjusted p = <0.0001.*

*Kruskal-Wallis rank-sum test: overall p = <0.0001; FDR-adjusted p = <0.0001.*

*Kruskal-Wallis rank-sum test: overall p = 0.0053; FDR-adjusted p = 0.0077.*

*Kruskal-Wallis rank-sum test: overall p = 0.0093; FDR-adjusted p = 0.0123.*

*Kruskal-Wallis rank-sum test: overall p = 0.0002; FDR-adjusted p = 0.0003.*

*Kruskal-Wallis rank-sum test: overall p = <0.0001; FDR-adjusted p = <0.0001.*

*Kruskal-Wallis rank-sum test: overall p = <0.0001; FDR-adjusted p = <0.0001.*

*Kruskal-Wallis rank-sum test: overall p = <0.0001; FDR-adjusted p = <0.0001.*

*Kruskal-Wallis rank-sum test: overall p = 0.0139; FDR-adjusted p = 0.0170.*

*Kruskal-Wallis rank-sum test: overall p = <0.0001; FDR-adjusted p = <0.0001.*

*Kruskal-Wallis rank-sum test: overall p = <0.0001; FDR-adjusted p = <0.0001.*

*Kruskal-Wallis rank-sum test: overall p = 0.0007; FDR-adjusted p = 0.0011.*

*Kruskal-Wallis rank-sum test: overall p = <0.0001; FDR-adjusted p = <0.0001.*

*Kruskal-Wallis rank-sum test: overall p = 0.0002; FDR-adjusted p = 0.0003.*

*Kruskal-Wallis rank-sum test: overall p = 0.0129; FDR-adjusted p = 0.0161.*

*Kruskal-Wallis rank-sum test: overall p = <0.0001; FDR-adjusted p = <0.0001.*

*Kruskal-Wallis rank-sum test: overall p = <0.0001; FDR-adjusted p = <0.0001.*

*Kruskal-Wallis rank-sum test: overall p = <0.0001; FDR-adjusted p = <0.0001.*

*Kruskal-Wallis rank-sum test: overall p = <0.0001; FDR-adjusted p = <0.0001.*

*Kruskal-Wallis rank-sum test: overall p = <0.0001; FDR-adjusted p = <0.0001.*
