# Supplementary material for: Sphingolipid signatures at the extremes of energy balance: a comparative study of obesity and anorexia nervosa in women
Source: Front Endocrinol (Lausanne). 2026 Oct 8;17:1890712. doi: 10.3389/fendo.2026.1890712 (PMC13688175; doi:10.3389/fendo.2026.1890712)
Supplement: Supplementary file 3 [file Table1.docx]

Table S1. Age-adjusted associations between FDR-significant plasma sphingolipids and related ratios and clinical/metabolic predictors.

Linear regression models were fitted for each of the 38 sphingolipid variables that were significant after Benjamini-Hochberg FDR correction in the global three-group comparison. Each model included one clinical/metabolic predictor and age as covariate. False discovery rate correction was applied globally across all 266 predictor-related tests.

| **Sphingolipid Variable** | **Predictor** | **Beta** | **SE** | **95% CI** | **Std.Beta** | **p** | **AdjR2** | **p-FDR** |
| --- | --- | --- | --- | --- | --- | --- | --- | --- |
| Cer 14 | BMI | -9.76e-05 | 4.29e-05 | -0.0002 to -1.26e-05 | -0.2139 | 0.0248 | 0.0275 | 0.0468 |
| Cer 14 | HOMA-IR | -0.0002 | 0.0002 | -0.0006 to 0.0002 | -0.0774 | 0.4151 | -0.0117 | 0.5208 |
| Cer 14 | DBP | -9.48e-05 | 5.28e-05 | -0.0002 to 9.88e-06 | -0.1722 | 0.0754 | 0.0109 | 0.1270 |
| Cer 14 | SBP | -9.46e-05 | 3.69e-05 | -0.0002 to -2.15e-05 | -0.2410 | 0.0117 | 0.0391 | 0.0239 |
| Cer 14 | CRP | -0.0009 | 0.0010 | -0.0028 to 0.0010 | -0.0879 | 0.3619 | -0.0102 | 0.4650 |
| Cer 14 | HDL-C | 5.19e-05 | 2.92e-05 | -6.03e-06 to 0.0001 | 0.1662 | 0.0786 | 0.0103 | 0.1298 |
| Cer 14 | TG | 4.43e-06 | 1.12e-05 | -1.78e-05 to 2.67e-05 | 0.0375 | 0.6935 | -0.0164 | 0.7468 |
| Cer 18 | BMI | 0.0017 | 0.0004 | 0.0010 to 0.0024 | 0.4296 | <0.0001 | 0.1712 | <0.0001 |
| Cer 18 | HOMA-IR | 0.0083 | 0.0016 | 0.0052 to 0.0115 | 0.4420 | <0.0001 | 0.1870 | <0.0001 |
| Cer 18 | DBP | 0.0023 | 0.0004 | 0.0014 to 0.0031 | 0.4592 | <0.0001 | 0.1922 | <0.0001 |
| Cer 18 | SBP | 0.0009 | 0.0003 | 0.0002 to 0.0015 | 0.2553 | 0.0073 | 0.0520 | 0.0163 |
| Cer 18 | CRP | 0.0358 | 0.0080 | 0.0200 to 0.0516 | 0.3963 | <0.0001 | 0.1434 | <0.0001 |
| Cer 18 | HDL-C | -0.0008 | 0.0003 | -0.0013 to -0.0003 | -0.3027 | 0.0011 | 0.0814 | 0.0034 |
| Cer 18 | TG | 0.0003 | 9.44e-05 | 0.0002 to 0.0005 | 0.3221 | 0.0005 | 0.0935 | 0.0018 |
| Cer 20 | BMI | -0.0020 | 0.0004 | -0.0028 to -0.0011 | -0.4037 | <0.0001 | 0.1935 | <0.0001 |
| Cer 20 | HOMA-IR | -0.0038 | 0.0021 | -0.0079 to 0.0003 | -0.1668 | 0.0702 | 0.0602 | 0.1189 |
| Cer 20 | DBP | -0.0007 | 0.0006 | -0.0018 to 0.0004 | -0.1141 | 0.2291 | 0.0445 | 0.3190 |
| Cer 20 | SBP | -0.0016 | 0.0004 | -0.0023 to -0.0008 | -0.3742 | <0.0001 | 0.1690 | 0.0002 |
| Cer 20 | CRP | -0.0178 | 0.0101 | -0.0378 to 0.0021 | -0.1640 | 0.0795 | 0.0585 | 0.1305 |
| Cer 20 | HDL-C | 0.0012 | 0.0003 | 0.0006 to 0.0018 | 0.3523 | <0.0001 | 0.1583 | 0.0004 |
| Cer 20 | TG | -6.88e-05 | 0.0001 | -0.0003 to 0.0002 | -0.0543 | 0.5587 | 0.0349 | 0.6490 |
| Cer 22 | BMI | -0.0021 | 0.0014 | -0.0050 to 0.0008 | -0.1370 | 0.1532 | 0.0007 | 0.2253 |
| Cer 22 | HOMA-IR | 0.0028 | 0.0067 | -0.0105 to 0.0160 | 0.0391 | 0.6809 | -0.0163 | 0.7371 |
| Cer 22 | DBP | 0.0009 | 0.0018 | -0.0026 to 0.0045 | 0.0501 | 0.6071 | -0.0154 | 0.6728 |
| Cer 22 | SBP | -0.0006 | 0.0013 | -0.0031 to 0.0019 | -0.0455 | 0.6390 | -0.0159 | 0.6994 |
| Cer 22 | CRP | -0.0038 | 0.0325 | -0.0682 to 0.0606 | -0.0112 | 0.9079 | -0.0178 | 0.9336 |
| Cer 22 | HDL-C | 0.0010 | 0.0010 | -0.0009 to 0.0030 | 0.1000 | 0.2917 | -0.0077 | 0.3861 |
| Cer 22 | TG | -3.16e-05 | 0.0004 | -0.0008 to 0.0007 | -0.0080 | 0.9328 | -0.0178 | 0.9434 |
| Cer 24 | BMI | -0.0681 | 0.0105 | -0.0889 to -0.0473 | -0.5303 | <0.0001 | 0.2661 | <0.0001 |
| Cer 24 | HOMA-IR | -0.1758 | 0.0538 | -0.2825 to -0.0691 | -0.2953 | 0.0015 | 0.0760 | 0.0042 |
| Cer 24 | DBP | -0.0389 | 0.0146 | -0.0678 to -0.0100 | -0.2510 | 0.0088 | 0.0482 | 0.0194 |
| Cer 24 | SBP | -0.0394 | 0.0100 | -0.0592 to -0.0197 | -0.3570 | 0.0001 | 0.1120 | 0.0006 |
| Cer 24 | CRP | -0.9225 | 0.2595 | -1.4367 to -0.4082 | -0.3236 | 0.0006 | 0.0907 | 0.0020 |
| Cer 24 | HDL-C | 0.0385 | 0.0075 | 0.0237 to 0.0533 | 0.4386 | <0.0001 | 0.1830 | <0.0001 |
| Cer 24 | TG | -0.0056 | 0.0031 | -0.0117 to 0.0006 | -0.1670 | 0.0767 | 0.0155 | 0.1283 |
| Cer 24:1 | BMI | -0.0095 | 0.0038 | -0.0170 to -0.0019 | -0.2263 | 0.0140 | 0.0950 | 0.0283 |
| Cer 24:1 | HOMA-IR | -0.0160 | 0.0178 | -0.0512 to 0.0192 | -0.0825 | 0.3700 | 0.0512 | 0.4712 |
| Cer 24:1 | DBP | -0.0026 | 0.0048 | -0.0120 to 0.0068 | -0.0511 | 0.5885 | 0.0468 | 0.6664 |
| Cer 24:1 | SBP | -0.0136 | 0.0031 | -0.0198 to -0.0074 | -0.3777 | <0.0001 | 0.1840 | 0.0001 |
| Cer 24:1 | CRP | -0.0356 | 0.0866 | -0.2071 to 0.1359 | -0.0384 | 0.6817 | 0.0457 | 0.7371 |
| Cer 24:1 | HDL-C | 0.0051 | 0.0026 | 1.30e-05 to 0.0103 | 0.1795 | 0.0494 | 0.0771 | 0.0871 |
| Cer 24:1 | TG | 0.0011 | 0.0010 | -0.0008 to 0.0031 | 0.1056 | 0.2512 | 0.0556 | 0.3409 |
| DHCer 16 | BMI | 0.0003 | 0.0001 | 6.12e-05 to 0.0005 | 0.2376 | 0.0115 | 0.0579 | 0.0238 |
| DHCer 16 | HOMA-IR | 0.0015 | 0.0005 | 0.0005 to 0.0024 | 0.2831 | 0.0021 | 0.0835 | 0.0058 |
| DHCer 16 | DBP | 0.0003 | 0.0001 | 2.24e-05 to 0.0005 | 0.2036 | 0.0333 | 0.0421 | 0.0619 |
| DHCer 16 | SBP | 0.0002 | 9.12e-05 | -5.49e-08 to 0.0004 | 0.1865 | 0.0501 | 0.0360 | 0.0876 |
| DHCer 16 | CRP | 0.0071 | 0.0023 | 0.0026 to 0.0116 | 0.2842 | 0.0024 | 0.0818 | 0.0063 |
| DHCer 16 | HDL-C | -0.0002 | 6.90e-05 | -0.0004 to -9.50e-05 | -0.3007 | 0.0011 | 0.0940 | 0.0034 |
| DHCer 16 | TG | 8.66e-05 | 2.62e-05 | 3.47e-05 to 0.0001 | 0.2969 | 0.0013 | 0.0915 | 0.0038 |
| DHCer 18 | BMI | 0.0006 | 6.66e-05 | 0.0004 to 0.0007 | 0.6535 | <0.0001 | 0.4056 | <0.0001 |
| DHCer 18 | HOMA-IR | 0.0027 | 0.0003 | 0.0021 to 0.0032 | 0.6847 | <0.0001 | 0.4586 | <0.0001 |
| DHCer 18 | DBP | 0.0005 | 9.80e-05 | 0.0003 to 0.0007 | 0.4392 | <0.0001 | 0.1704 | <0.0001 |
| DHCer 18 | SBP | 0.0003 | 7.30e-05 | 0.0002 to 0.0005 | 0.4015 | <0.0001 | 0.1393 | 0.0002 |
| DHCer 18 | CRP | 0.0095 | 0.0016 | 0.0063 to 0.0127 | 0.5153 | <0.0001 | 0.2435 | <0.0001 |
| DHCer 18 | HDL-C | -0.0003 | 4.98e-05 | -0.0004 to -0.0002 | -0.5609 | <0.0001 | 0.3013 | <0.0001 |
| DHCer 18 | TG | 0.0001 | 1.75e-05 | 9.01e-05 to 0.0002 | 0.5815 | <0.0001 | 0.3240 | <0.0001 |
| DHCer 18:1 | BMI | 0.0003 | 3.95e-05 | 0.0002 to 0.0004 | 0.5430 | <0.0001 | 0.3536 | <0.0001 |
| DHCer 18:1 | HOMA-IR | 0.0011 | 0.0002 | 0.0007 to 0.0014 | 0.4429 | <0.0001 | 0.2609 | <0.0001 |
| DHCer 18:1 | DBP | 0.0002 | 5.36e-05 | 0.0001 to 0.0003 | 0.3818 | <0.0001 | 0.2023 | 0.0001 |
| DHCer 18:1 | SBP | 0.0002 | 3.68e-05 | 0.0001 to 0.0003 | 0.4438 | <0.0001 | 0.2541 | <0.0001 |
| DHCer 18:1 | CRP | 0.0053 | 0.0009 | 0.0034 to 0.0071 | 0.4605 | <0.0001 | 0.2708 | <0.0001 |
| DHCer 18:1 | HDL-C | -0.0002 | 2.81e-05 | -0.0002 to -0.0001 | -0.4685 | <0.0001 | 0.2847 | <0.0001 |
| DHCer 18:1 | TG | 3.11e-05 | 1.18e-05 | 7.67e-06 to 5.45e-05 | 0.2329 | 0.0097 | 0.1163 | 0.0209 |
| DHCer 24:1 | BMI | 0.0029 | 0.0006 | 0.0017 to 0.0040 | 0.4311 | <0.0001 | 0.1687 | <0.0001 |
| DHCer 24:1 | HOMA-IR | 0.0158 | 0.0025 | 0.0108 to 0.0208 | 0.5095 | <0.0001 | 0.2487 | <0.0001 |
| DHCer 24:1 | DBP | 0.0025 | 0.0007 | 0.0010 to 0.0040 | 0.3059 | 0.0013 | 0.0750 | 0.0039 |
| DHCer 24:1 | SBP | 0.0009 | 0.0006 | -0.0002 to 0.0020 | 0.1542 | 0.1093 | 0.0078 | 0.1710 |
| DHCer 24:1 | CRP | 0.0588 | 0.0132 | 0.0327 to 0.0849 | 0.3956 | <0.0001 | 0.1392 | 0.0001 |
| DHCer 24:1 | HDL-C | -0.0019 | 0.0004 | -0.0027 to -0.0012 | -0.4235 | <0.0001 | 0.1671 | <0.0001 |
| DHCer 24:1 | TG | 0.0010 | 0.0001 | 0.0007 to 0.0013 | 0.5819 | <0.0001 | 0.3284 | <0.0001 |
| SM 16 | BMI | -1.9511 | 0.2694 | -2.4849 to -1.4173 | -0.5680 | <0.0001 | 0.3229 | <0.0001 |
| SM 16 | HOMA-IR | -5.5000 | 1.4016 | -8.2773 to -2.7227 | -0.3455 | 0.0002 | 0.1243 | 0.0006 |
| SM 16 | DBP | -1.2436 | 0.3813 | -1.9992 to -0.4880 | -0.3002 | 0.0015 | 0.0901 | 0.0042 |
| SM 16 | SBP | -1.5605 | 0.2410 | -2.0379 to -1.0830 | -0.5284 | <0.0001 | 0.2763 | <0.0001 |
| SM 16 | CRP | -25.9101 | 6.8372 | -39.4585 to -12.3617 | -0.3399 | 0.0002 | 0.1171 | 0.0009 |
| SM 16 | HDL-C | 1.1168 | 0.1936 | 0.7332 to 1.5004 | 0.4753 | <0.0001 | 0.2328 | <0.0001 |
| SM 16 | TG | -0.1053 | 0.0830 | -0.2697 to 0.0592 | -0.1184 | 0.2075 | 0.0171 | 0.2935 |
| SM 18 | BMI | 0.0613 | 0.1100 | -0.1567 to 0.2794 | 0.0532 | 0.5783 | -0.0041 | 0.6630 |
| SM 18 | HOMA-IR | 1.0155 | 0.4948 | 0.0351 to 1.9959 | 0.1902 | 0.0425 | 0.0299 | 0.0758 |
| SM 18 | DBP | 0.3438 | 0.1305 | 0.0852 to 0.6024 | 0.2474 | 0.0096 | 0.0523 | 0.0208 |
| SM 18 | SBP | 0.0180 | 0.0953 | -0.1709 to 0.2068 | 0.0181 | 0.8509 | -0.0066 | 0.8842 |
| SM 18 | CRP | 2.7957 | 2.4345 | -2.0284 to 7.6198 | 0.1094 | 0.2533 | 0.0049 | 0.3420 |
| SM 18 | HDL-C | -0.0020 | 0.0744 | -0.1494 to 0.1454 | -0.0025 | 0.9787 | -0.0069 | 0.9787 |
| SM 18 | TG | 0.0463 | 0.0278 | -0.0089 to 0.1014 | 0.1551 | 0.0994 | 0.0175 | 0.1593 |
| SM 18:1 | BMI | 0.1487 | 0.0573 | 0.0352 to 0.2622 | 0.2419 | 0.0107 | 0.0435 | 0.0225 |
| SM 18:1 | HOMA-IR | 0.7827 | 0.2595 | 0.2684 to 1.2970 | 0.2748 | 0.0032 | 0.0623 | 0.0081 |
| SM 18:1 | DBP | 0.2859 | 0.0667 | 0.1536 to 0.4181 | 0.3856 | <0.0001 | 0.1294 | 0.0002 |
| SM 18:1 | SBP | 0.1143 | 0.0499 | 0.0155 to 0.2132 | 0.2163 | 0.0238 | 0.0313 | 0.0453 |
| SM 18:1 | CRP | 1.8805 | 1.2993 | -0.6942 to 4.4552 | 0.1379 | 0.1506 | 0.0042 | 0.2251 |
| SM 18:1 | HDL-C | -0.0364 | 0.0397 | -0.1151 to 0.0422 | -0.0867 | 0.3605 | -0.0069 | 0.4650 |
| SM 18:1 | TG | 0.0497 | 0.0143 | 0.0213 to 0.0781 | 0.3122 | 0.0008 | 0.0845 | 0.0025 |
| SM 24 | BMI | -1.4742 | 0.1868 | -1.8444 to -1.1040 | -0.5969 | <0.0001 | 0.3701 | <0.0001 |
| SM 24 | HOMA-IR | -3.2206 | 1.0232 | -5.2481 to -1.1931 | -0.2813 | 0.0021 | 0.0974 | 0.0057 |
| SM 24 | DBP | -0.9769 | 0.2695 | -1.5110 to -0.4429 | -0.3280 | 0.0004 | 0.1209 | 0.0016 |
| SM 24 | SBP | -1.1333 | 0.1709 | -1.4720 to -0.7946 | -0.5337 | <0.0001 | 0.2958 | <0.0001 |
| SM 24 | CRP | -16.5396 | 4.9448 | -26.3380 to -6.7413 | -0.3018 | 0.0011 | 0.1069 | 0.0035 |
| SM 24 | HDL-C | 0.7715 | 0.1395 | 0.4950 to 1.0480 | 0.4567 | <0.0001 | 0.2291 | <0.0001 |
| SM 24 | TG | -0.1132 | 0.0587 | -0.2295 to 0.0032 | -0.1770 | 0.0565 | 0.0487 | 0.0977 |
| SM 24:1 | BMI | -2.5139 | 0.3752 | -3.2573 to -1.7704 | -0.5279 | <0.0001 | 0.3166 | <0.0001 |
| SM 24:1 | HOMA-IR | -6.5776 | 1.9361 | -10.4140 to -2.7411 | -0.2980 | 0.0009 | 0.1307 | 0.0031 |
| SM 24:1 | DBP | -1.7606 | 0.5166 | -2.7842 to -0.7370 | -0.3065 | 0.0009 | 0.1312 | 0.0030 |
| SM 24:1 | SBP | -2.3610 | 0.3127 | -2.9806 to -1.7414 | -0.5766 | <0.0001 | 0.3659 | <0.0001 |
| SM 24:1 | CRP | -28.0359 | 9.5178 | -46.8962 to -9.1757 | -0.2653 | 0.0039 | 0.1099 | 0.0100 |
| SM 24:1 | HDL-C | 1.2943 | 0.2739 | 0.7515 to 1.8371 | 0.3973 | <0.0001 | 0.2010 | <0.0001 |
| SM 24:1 | TG | -0.1318 | 0.1130 | -0.3558 to 0.0921 | -0.1070 | 0.2459 | 0.0519 | 0.3389 |
| HexCer 18:1 | BMI | 0.0006 | 0.0001 | 0.0003 to 0.0008 | 0.3846 | <0.0001 | 0.1801 | 0.0001 |
| HexCer 18:1 | HOMA-IR | 0.0027 | 0.0006 | 0.0016 to 0.0039 | 0.3895 | <0.0001 | 0.1878 | <0.0001 |
| HexCer 18:1 | DBP | 0.0004 | 0.0002 | 0.0001 to 0.0008 | 0.2407 | 0.0102 | 0.0895 | 0.0215 |
| HexCer 18:1 | SBP | 0.0005 | 0.0001 | 0.0003 to 0.0008 | 0.4131 | <0.0001 | 0.2005 | <0.0001 |
| HexCer 18:1 | CRP | 0.0086 | 0.0030 | 0.0026 to 0.0147 | 0.2566 | 0.0055 | 0.0985 | 0.0130 |
| HexCer 18:1 | HDL-C | -0.0004 | 8.77e-05 | -0.0006 to -0.0002 | -0.3915 | <0.0001 | 0.1895 | <0.0001 |
| HexCer 18:1 | TG | 9.81e-05 | 3.51e-05 | 2.87e-05 to 0.0002 | 0.2504 | 0.0060 | 0.0972 | 0.0140 |
| HexCer 20 | BMI | -0.0031 | 0.0011 | -0.0053 to -0.0009 | -0.2581 | 0.0062 | 0.0596 | 0.0141 |
| HexCer 20 | HOMA-IR | -0.0005 | 0.0053 | -0.0109 to 0.0099 | -0.0090 | 0.9242 | -0.0064 | 0.9434 |
| HexCer 20 | DBP | 0.0008 | 0.0014 | -0.0020 to 0.0035 | 0.0520 | 0.5920 | -0.0039 | 0.6664 |
| HexCer 20 | SBP | -0.0016 | 0.0010 | -0.0035 to 0.0004 | -0.1513 | 0.1147 | 0.0159 | 0.1785 |
| HexCer 20 | CRP | -0.0331 | 0.0254 | -0.0833 to 0.0172 | -0.1238 | 0.1955 | 0.0087 | 0.2782 |
| HexCer 20 | HDL-C | 0.0016 | 0.0008 | 0.0001 to 0.0031 | 0.1968 | 0.0356 | 0.0329 | 0.0653 |
| HexCer 20 | TG | -0.0002 | 0.0003 | -0.0007 to 0.0004 | -0.0490 | 0.6044 | -0.0041 | 0.6727 |
| HexCer 22 | BMI | -0.0698 | 0.0108 | -0.0911 to -0.0485 | -0.5306 | <0.0001 | 0.2639 | <0.0001 |
| HexCer 22 | HOMA-IR | -0.0764 | 0.0573 | -0.1900 to 0.0371 | -0.1254 | 0.1850 | 0.0006 | 0.2646 |
| HexCer 22 | DBP | -0.0334 | 0.0151 | -0.0633 to -0.0035 | -0.2104 | 0.0291 | 0.0275 | 0.0545 |
| HexCer 22 | SBP | -0.0357 | 0.0104 | -0.0562 to -0.0151 | -0.3154 | 0.0008 | 0.0821 | 0.0028 |
| HexCer 22 | CRP | -0.8615 | 0.2685 | -1.3935 to -0.3295 | -0.2953 | 0.0017 | 0.0708 | 0.0048 |
| HexCer 22 | HDL-C | 0.0379 | 0.0077 | 0.0226 to 0.0532 | 0.4212 | <0.0001 | 0.1652 | <0.0001 |
| HexCer 22 | TG | -0.0056 | 0.0032 | -0.0119 to 0.0007 | -0.1643 | 0.0820 | 0.0120 | 0.1338 |
| HexCer 24 | BMI | -0.0765 | 0.0146 | -0.1054 to -0.0476 | -0.4507 | <0.0001 | 0.1871 | <0.0001 |
| HexCer 24 | HOMA-IR | -0.0772 | 0.0742 | -0.2243 to 0.0698 | -0.0982 | 0.3001 | -0.0045 | 0.3932 |
| HexCer 24 | DBP | -0.0419 | 0.0195 | -0.0805 to -0.0033 | -0.2046 | 0.0339 | 0.0262 | 0.0625 |
| HexCer 24 | SBP | -0.0569 | 0.0130 | -0.0827 to -0.0311 | -0.3896 | <0.0001 | 0.1344 | 0.0001 |
| HexCer 24 | CRP | -0.8425 | 0.3533 | -1.5426 to -0.1425 | -0.2236 | 0.0188 | 0.0352 | 0.0365 |
| HexCer 24 | HDL-C | 0.0372 | 0.0104 | 0.0166 to 0.0579 | 0.3205 | 0.0005 | 0.0903 | 0.0019 |
| HexCer 24 | TG | -0.0022 | 0.0042 | -0.0105 to 0.0060 | -0.0503 | 0.5962 | -0.0117 | 0.6664 |
| HexCer 24:1 | BMI | -0.0367 | 0.0175 | -0.0712 to -0.0021 | -0.1952 | 0.0379 | 0.0501 | 0.0690 |
| HexCer 24:1 | HOMA-IR | -0.0072 | 0.0814 | -0.1685 to 0.1541 | -0.0082 | 0.9299 | 0.0124 | 0.9434 |
| HexCer 24:1 | DBP | 0.0059 | 0.0217 | -0.0372 to 0.0489 | 0.0259 | 0.7873 | 0.0130 | 0.8343 |
| HexCer 24:1 | SBP | -0.0478 | 0.0147 | -0.0770 to -0.0187 | -0.2962 | 0.0015 | 0.0983 | 0.0042 |
| HexCer 24:1 | CRP | -0.2281 | 0.3949 | -1.0107 to 0.5545 | -0.0547 | 0.5648 | 0.0153 | 0.6503 |
| HexCer 24:1 | HDL-C | 0.0160 | 0.0119 | -0.0076 to 0.0396 | 0.1243 | 0.1829 | 0.0281 | 0.2630 |
| HexCer 24:1 | TG | 0.0041 | 0.0045 | -0.0049 to 0.0131 | 0.0839 | 0.3703 | 0.0195 | 0.4712 |
| LacCer 18 | BMI | 0.0008 | 0.0006 | -0.0003 to 0.0019 | 0.1340 | 0.1622 | 0.0017 | 0.2371 |
| LacCer 18 | HOMA-IR | 0.0072 | 0.0026 | 0.0021 to 0.0123 | 0.2570 | 0.0059 | 0.0512 | 0.0139 |
| LacCer 18 | DBP | 0.0023 | 0.0007 | 0.0009 to 0.0036 | 0.3096 | 0.0011 | 0.0766 | 0.0035 |
| LacCer 18 | SBP | 0.0009 | 0.0005 | -5.28e-05 to 0.0019 | 0.1784 | 0.0635 | 0.0151 | 0.1082 |
| LacCer 18 | CRP | 0.0194 | 0.0128 | -0.0059 to 0.0446 | 0.1447 | 0.1316 | 0.0046 | 0.2023 |
| LacCer 18 | HDL-C | -0.0006 | 0.0004 | -0.0014 to 0.0001 | -0.1533 | 0.1047 | 0.0078 | 0.1668 |
| LacCer 18 | TG | 0.0001 | 0.0001 | -0.0002 to 0.0004 | 0.0696 | 0.4641 | -0.0112 | 0.5611 |
| LacCer 18:1 | BMI | -0.0004 | 0.0002 | -0.0008 to 2.06e-05 | -0.1782 | 0.0622 | 0.0149 | 0.1067 |
| LacCer 18:1 | HOMA-IR | 0.0014 | 0.0010 | -0.0005 to 0.0033 | 0.1357 | 0.1517 | 0.0021 | 0.2253 |
| LacCer 18:1 | DBP | 0.0002 | 0.0003 | -0.0003 to 0.0007 | 0.0718 | 0.4611 | -0.0116 | 0.5601 |
| LacCer 18:1 | SBP | -0.0002 | 0.0002 | -0.0006 to 0.0001 | -0.1134 | 0.2406 | -0.0040 | 0.3333 |
| LacCer 18:1 | CRP | -0.0010 | 0.0048 | -0.0104 to 0.0085 | -0.0197 | 0.8380 | -0.0162 | 0.8741 |
| LacCer 18:1 | HDL-C | 0.0001 | 0.0001 | -0.0002 to 0.0004 | 0.0687 | 0.4694 | -0.0118 | 0.5650 |
| LacCer 18:1 | TG | 3.00e-05 | 5.49e-05 | -7.88e-05 to 0.0001 | 0.0518 | 0.5858 | -0.0139 | 0.6664 |
| LacCer 20 | BMI | -0.0016 | 0.0004 | -0.0023 to -0.0008 | -0.3549 | 0.0001 | 0.1108 | 0.0006 |
| LacCer 20 | HOMA-IR | -0.0016 | 0.0019 | -0.0054 to 0.0022 | -0.0768 | 0.4180 | -0.0081 | 0.5220 |
| LacCer 20 | DBP | -0.0001 | 0.0005 | -0.0012 to 0.0009 | -0.0282 | 0.7719 | -0.0133 | 0.8246 |
| LacCer 20 | SBP | -0.0009 | 0.0004 | -0.0016 to -0.0001 | -0.2261 | 0.0180 | 0.0360 | 0.0352 |
| LacCer 20 | CRP | -0.0135 | 0.0093 | -0.0318 to 0.0049 | -0.1383 | 0.1492 | 0.0048 | 0.2242 |
| LacCer 20 | HDL-C | 0.0007 | 0.0003 | 0.0002 to 0.0013 | 0.2414 | 0.0098 | 0.0452 | 0.0210 |
| LacCer 20 | TG | -0.0001 | 0.0001 | -0.0003 to 0.0001 | -0.0939 | 0.3224 | -0.0052 | 0.4204 |
| LacCer 22 | BMI | -0.0091 | 0.0020 | -0.0131 to -0.0052 | -0.4045 | <0.0001 | 0.1499 | <0.0001 |
| LacCer 22 | HOMA-IR | -0.0053 | 0.0099 | -0.0249 to 0.0143 | -0.0510 | 0.5906 | -0.0097 | 0.6664 |
| LacCer 22 | DBP | -0.0028 | 0.0026 | -0.0080 to 0.0024 | -0.1019 | 0.2940 | -0.0023 | 0.3872 |
| LacCer 22 | SBP | -0.0052 | 0.0018 | -0.0088 to -0.0016 | -0.2672 | 0.0049 | 0.0575 | 0.0118 |
| LacCer 22 | CRP | -0.0718 | 0.0476 | -0.1662 to 0.0226 | -0.1434 | 0.1344 | 0.0079 | 0.2054 |
| LacCer 22 | HDL-C | 0.0035 | 0.0014 | 0.0007 to 0.0063 | 0.2268 | 0.0154 | 0.0400 | 0.0308 |
| LacCer 22 | TG | -0.0007 | 0.0005 | -0.0018 to 0.0003 | -0.1265 | 0.1810 | 0.0039 | 0.2617 |
| LacCer 24 | BMI | -0.0083 | 0.0020 | -0.0123 to -0.0043 | -0.3695 | <0.0001 | 0.1217 | 0.0003 |
| LacCer 24 | HOMA-IR | 0.0027 | 0.0098 | -0.0168 to 0.0222 | 0.0260 | 0.7844 | -0.0130 | 0.8343 |
| LacCer 24 | DBP | -0.0032 | 0.0026 | -0.0084 to 0.0020 | -0.1184 | 0.2226 | -0.0002 | 0.3117 |
| LacCer 24 | SBP | -0.0059 | 0.0018 | -0.0095 to -0.0024 | -0.3080 | 0.0011 | 0.0792 | 0.0035 |
| LacCer 24 | CRP | -0.0405 | 0.0477 | -0.1350 to 0.0540 | -0.0813 | 0.3978 | -0.0072 | 0.5015 |
| LacCer 24 | HDL-C | 0.0025 | 0.0014 | -0.0004 to 0.0053 | 0.1597 | 0.0903 | 0.0123 | 0.1465 |
| LacCer 24 | TG | -0.0005 | 0.0005 | -0.0016 to 0.0006 | -0.0881 | 0.3530 | -0.0058 | 0.4580 |
| LacCer 24:1 | BMI | -0.0461 | 0.0107 | -0.0672 to -0.0249 | -0.3825 | <0.0001 | 0.1370 | 0.0002 |
| LacCer 24:1 | HOMA-IR | -0.0046 | 0.0527 | -0.1091 to 0.0999 | -0.0083 | 0.9305 | -0.0081 | 0.9434 |
| LacCer 24:1 | DBP | -0.0161 | 0.0140 | -0.0439 to 0.0116 | -0.1111 | 0.2512 | 0.0038 | 0.3409 |
| LacCer 24:1 | SBP | -0.0335 | 0.0095 | -0.0522 to -0.0147 | -0.3231 | 0.0006 | 0.0941 | 0.0021 |
| LacCer 24:1 | CRP | -0.2003 | 0.2556 | -0.7067 to 0.3061 | -0.0749 | 0.4348 | -0.0026 | 0.5379 |
| LacCer 24:1 | HDL-C | 0.0153 | 0.0076 | 0.0002 to 0.0305 | 0.1859 | 0.0475 | 0.0270 | 0.0843 |
| LacCer 24:1 | TG | -0.0021 | 0.0029 | -0.0079 to 0.0037 | -0.0669 | 0.4800 | -0.0036 | 0.5751 |
| GM3 16 | BMI | -0.0102 | 0.0076 | -0.0252 to 0.0048 | -0.1280 | 0.1798 | 0.0102 | 0.2613 |
| GM3 16 | HOMA-IR | 0.0185 | 0.0349 | -0.0505 to 0.0876 | 0.0501 | 0.5959 | -0.0035 | 0.6664 |
| GM3 16 | DBP | 0.0073 | 0.0093 | -0.0111 to 0.0258 | 0.0764 | 0.4306 | -0.0004 | 0.5352 |
| GM3 16 | SBP | -0.0106 | 0.0065 | -0.0235 to 0.0023 | -0.1549 | 0.1061 | 0.0175 | 0.1671 |
| GM3 16 | CRP | 0.0813 | 0.1694 | -0.2543 to 0.4169 | 0.0459 | 0.6322 | -0.0039 | 0.6949 |
| GM3 16 | HDL-C | 0.0018 | 0.0051 | -0.0084 to 0.0120 | 0.0333 | 0.7243 | -0.0049 | 0.7769 |
| GM3 16 | TG | -0.0001 | 0.0020 | -0.0040 to 0.0037 | -0.0066 | 0.9448 | -0.0060 | 0.9519 |
| GM3 18:1 | BMI | 0.0007 | 0.0002 | 0.0003 to 0.0011 | 0.3146 | 0.0007 | 0.1027 | 0.0024 |
| GM3 18:1 | HOMA-IR | 0.0045 | 0.0009 | 0.0027 to 0.0062 | 0.4276 | <0.0001 | 0.1906 | <0.0001 |
| GM3 18:1 | DBP | 0.0007 | 0.0003 | 0.0002 to 0.0012 | 0.2632 | 0.0055 | 0.0716 | 0.0130 |
| GM3 18:1 | SBP | 0.0006 | 0.0002 | 0.0002 to 0.0009 | 0.2854 | 0.0024 | 0.0843 | 0.0063 |
| GM3 18:1 | CRP | 0.0111 | 0.0047 | 0.0019 to 0.0203 | 0.2211 | 0.0190 | 0.0528 | 0.0367 |
| GM3 18:1 | HDL-C | -0.0005 | 0.0001 | -0.0008 to -0.0003 | -0.3513 | 0.0001 | 0.1301 | 0.0005 |
| GM3 18:1 | TG | 6.36e-05 | 5.47e-05 | -4.48e-05 to 0.0002 | 0.1086 | 0.2474 | 0.0165 | 0.3393 |
| GM3 20 | BMI | -0.0034 | 0.0009 | -0.0052 to -0.0017 | -0.3514 | 0.0002 | 0.1044 | 0.0006 |
| GM3 20 | HOMA-IR | -0.0005 | 0.0043 | -0.0090 to 0.0080 | -0.0109 | 0.9090 | -0.0179 | 0.9336 |
| GM3 20 | DBP | -0.0007 | 0.0011 | -0.0030 to 0.0016 | -0.0598 | 0.5398 | -0.0146 | 0.6325 |
| GM3 20 | SBP | -0.0026 | 0.0008 | -0.0041 to -0.0010 | -0.3052 | 0.0013 | 0.0732 | 0.0038 |
| GM3 20 | CRP | -0.0179 | 0.0208 | -0.0591 to 0.0232 | -0.0830 | 0.3894 | -0.0112 | 0.4932 |
| GM3 20 | HDL-C | 0.0011 | 0.0006 | -0.0002 to 0.0023 | 0.1597 | 0.0911 | 0.0079 | 0.1469 |
| GM3 20 | TG | -1.06e-05 | 0.0002 | -0.0005 to 0.0005 | -0.0042 | 0.9648 | -0.0180 | 0.9684 |
| GM3 22 | BMI | -0.0063 | 0.0044 | -0.0150 to 0.0023 | -0.1381 | 0.1490 | 0.0056 | 0.2242 |
| GM3 22 | HOMA-IR | 0.0117 | 0.0201 | -0.0282 to 0.0516 | 0.0549 | 0.5630 | -0.0102 | 0.6503 |
| GM3 22 | DBP | 0.0040 | 0.0054 | -0.0066 to 0.0146 | 0.0723 | 0.4570 | -0.0082 | 0.5576 |
| GM3 22 | SBP | -0.0019 | 0.0038 | -0.0094 to 0.0056 | -0.0482 | 0.6179 | -0.0110 | 0.6820 |
| GM3 22 | CRP | -0.0686 | 0.0977 | -0.2622 to 0.1250 | -0.0674 | 0.4838 | -0.0088 | 0.5771 |
| GM3 22 | HDL-C | 0.0023 | 0.0030 | -0.0036 to 0.0081 | 0.0722 | 0.4465 | -0.0080 | 0.5474 |
| GM3 22 | TG | -0.0012 | 0.0011 | -0.0034 to 0.0010 | -0.1025 | 0.2793 | -0.0026 | 0.3715 |
| GM3 24 | BMI | -0.0073 | 0.0013 | -0.0099 to -0.0047 | -0.4693 | <0.0001 | 0.2086 | <0.0001 |
| GM3 24 | HOMA-IR | -0.0156 | 0.0066 | -0.0288 to -0.0025 | -0.2178 | 0.0200 | 0.0384 | 0.0382 |
| GM3 24 | DBP | -0.0043 | 0.0018 | -0.0078 to -0.0008 | -0.2291 | 0.0169 | 0.0410 | 0.0336 |
| GM3 24 | SBP | -0.0052 | 0.0012 | -0.0075 to -0.0028 | -0.3865 | <0.0001 | 0.1365 | 0.0002 |
| GM3 24 | CRP | -0.0958 | 0.0317 | -0.1587 to -0.0330 | -0.2786 | 0.0031 | 0.0669 | 0.0081 |
| GM3 24 | HDL-C | 0.0028 | 0.0010 | 0.0009 to 0.0048 | 0.2676 | 0.0040 | 0.0630 | 0.0101 |
| GM3 24 | TG | -0.0010 | 0.0004 | -0.0017 to -0.0003 | -0.2542 | 0.0064 | 0.0558 | 0.0145 |
| GM3 24:1 | BMI | -0.0099 | 0.0050 | -0.0199 to 0.0001 | -0.1838 | 0.0526 | 0.0316 | 0.0915 |
| GM3 24:1 | HOMA-IR | -0.0255 | 0.0234 | -0.0718 to 0.0208 | -0.1022 | 0.2774 | 0.0088 | 0.3708 |
| GM3 24:1 | DBP | 0.0017 | 0.0063 | -0.0107 to 0.0141 | 0.0257 | 0.7910 | -0.0013 | 0.8349 |
| GM3 24:1 | SBP | -0.0137 | 0.0042 | -0.0221 to -0.0053 | -0.2964 | 0.0016 | 0.0842 | 0.0045 |
| GM3 24:1 | CRP | -0.1678 | 0.1130 | -0.3916 to 0.0561 | -0.1405 | 0.1403 | 0.0176 | 0.2133 |
| GM3 24:1 | HDL-C | 0.0038 | 0.0034 | -0.0030 to 0.0107 | 0.1046 | 0.2666 | 0.0092 | 0.3582 |
| GM3 24:1 | TG | -0.0008 | 0.0013 | -0.0034 to 0.0018 | -0.0559 | 0.5534 | 0.0013 | 0.6457 |
| S1P | BMI | -0.0244 | 0.0091 | -0.0423 to -0.0064 | -0.2497 | 0.0083 | 0.0504 | 0.0184 |
| S1P | HOMA-IR | -0.0268 | 0.0427 | -0.1114 to 0.0578 | -0.0593 | 0.5317 | -0.0079 | 0.6259 |
| S1P | DBP | 0.0029 | 0.0114 | -0.0197 to 0.0255 | 0.0248 | 0.7989 | -0.0109 | 0.8366 |
| S1P | SBP | -0.0033 | 0.0081 | -0.0194 to 0.0127 | -0.0397 | 0.6815 | -0.0099 | 0.7371 |
| S1P | CRP | -0.1590 | 0.2073 | -0.5699 to 0.2518 | -0.0734 | 0.4447 | -0.0061 | 0.5474 |
| S1P | HDL-C | 0.0233 | 0.0059 | 0.0116 to 0.0350 | 0.3490 | 0.0001 | 0.1125 | 0.0006 |
| S1P | TG | 0.0003 | 0.0024 | -0.0044 to 0.0051 | 0.0132 | 0.8892 | -0.0113 | 0.9203 |
| DhSph | BMI | 0.0006 | 9.29e-05 | 0.0004 to 0.0007 | 0.4930 | <0.0001 | 0.2375 | <0.0001 |
| DhSph | HOMA-IR | 0.0018 | 0.0005 | 0.0009 to 0.0027 | 0.3488 | 0.0001 | 0.1203 | 0.0006 |
| DhSph | DBP | 0.0004 | 0.0001 | 0.0002 to 0.0007 | 0.3072 | 0.0012 | 0.0878 | 0.0035 |
| DhSph | SBP | 0.0005 | 8.06e-05 | 0.0003 to 0.0006 | 0.4923 | <0.0001 | 0.2339 | <0.0001 |
| DhSph | CRP | 0.0066 | 0.0023 | 0.0021 to 0.0112 | 0.2684 | 0.0043 | 0.0677 | 0.0106 |
| DhSph | HDL-C | -0.0003 | 6.55e-05 | -0.0004 to -0.0002 | -0.4114 | <0.0001 | 0.1688 | <0.0001 |
| DhSph | TG | 5.56e-05 | 2.67e-05 | 2.61e-06 to 0.0001 | 0.1924 | 0.0399 | 0.0341 | 0.0717 |
| DhS1P | BMI | -0.0202 | 0.0039 | -0.0279 to -0.0125 | -0.4481 | <0.0001 | 0.1867 | <0.0001 |
| DhS1P | HOMA-IR | -0.0466 | 0.0193 | -0.0849 to -0.0084 | -0.2231 | 0.0172 | 0.0382 | 0.0340 |
| DhS1P | DBP | -0.0085 | 0.0052 | -0.0189 to 0.0018 | -0.1567 | 0.1054 | 0.0113 | 0.1668 |
| DhS1P | SBP | -0.0104 | 0.0036 | -0.0176 to -0.0033 | -0.2684 | 0.0047 | 0.0581 | 0.0114 |
| DhS1P | CRP | -0.2413 | 0.0934 | -0.4263 to -0.0562 | -0.2410 | 0.0111 | 0.0449 | 0.0230 |
| DhS1P | HDL-C | 0.0148 | 0.0026 | 0.0098 to 0.0199 | 0.4812 | <0.0001 | 0.2232 | <0.0001 |
| DhS1P | TG | -0.0014 | 0.0011 | -0.0035 to 0.0008 | -0.1173 | 0.2154 | 0.0015 | 0.3031 |
| CER16/24 | BMI | 0.0058 | 0.0020 | 0.0019 to 0.0097 | 0.2717 | 0.0041 | 0.0552 | 0.0101 |
| CER16/24 | HOMA-IR | 0.0165 | 0.0093 | -0.0019 to 0.0348 | 0.1664 | 0.0781 | 0.0102 | 0.1298 |
| CER16/24 | DBP | -0.0007 | 0.0025 | -0.0056 to 0.0043 | -0.0253 | 0.7952 | -0.0174 | 0.8360 |
| CER16/24 | SBP | 0.0025 | 0.0018 | -0.0010 to 0.0060 | 0.1379 | 0.1533 | 0.0006 | 0.2253 |
| CER16/24 | CRP | 0.0932 | 0.0447 | 0.0046 to 0.1819 | 0.1970 | 0.0394 | 0.0204 | 0.0713 |
| CER16/24 | HDL-C | -0.0034 | 0.0013 | -0.0061 to -0.0007 | -0.2326 | 0.0132 | 0.0371 | 0.0268 |
| CER16/24 | TG | 0.0008 | 0.0005 | -0.0002 to 0.0018 | 0.1437 | 0.1292 | 0.0030 | 0.1998 |
| CER18/24 | BMI | 0.0016 | 0.0002 | 0.0012 to 0.0020 | 0.6029 | <0.0001 | 0.3442 | <0.0001 |
| CER18/24 | HOMA-IR | 0.0053 | 0.0011 | 0.0032 to 0.0074 | 0.4241 | <0.0001 | 0.1668 | <0.0001 |
| CER18/24 | DBP | 0.0009 | 0.0003 | 0.0003 to 0.0015 | 0.2823 | 0.0031 | 0.0608 | 0.0081 |
| CER18/24 | SBP | 0.0008 | 0.0002 | 0.0004 to 0.0013 | 0.3684 | <0.0001 | 0.1167 | 0.0004 |
| CER18/24 | CRP | 0.0243 | 0.0052 | 0.0139 to 0.0347 | 0.4091 | <0.0001 | 0.1492 | <0.0001 |
| CER18/24 | HDL-C | -0.0009 | 0.0002 | -0.0012 to -0.0006 | -0.4805 | <0.0001 | 0.2187 | <0.0001 |
| CER18/24 | TG | 0.0002 | 6.28e-05 | 8.23e-05 to 0.0003 | 0.2983 | 0.0013 | 0.0741 | 0.0039 |
| CER24:1/24 | BMI | 0.0068 | 0.0017 | 0.0034 to 0.0102 | 0.3521 | 0.0001 | 0.1187 | 0.0006 |
| CER24:1/24 | HOMA-IR | 0.0216 | 0.0082 | 0.0054 to 0.0378 | 0.2411 | 0.0096 | 0.0550 | 0.0208 |
| CER24:1/24 | DBP | 0.0015 | 0.0023 | -0.0030 to 0.0060 | 0.0643 | 0.5067 | -0.0002 | 0.6003 |
| CER24:1/24 | SBP | 0.0011 | 0.0016 | -0.0021 to 0.0042 | 0.0637 | 0.5078 | -0.0002 | 0.6003 |
| CER24:1/24 | CRP | 0.1160 | 0.0395 | 0.0377 to 0.1944 | 0.2704 | 0.0041 | 0.0681 | 0.0101 |
| CER24:1/24 | HDL-C | -0.0041 | 0.0012 | -0.0065 to -0.0018 | -0.3116 | 0.0007 | 0.0947 | 0.0024 |
| CER24:1/24 | TG | 0.0013 | 0.0005 | 0.0004 to 0.0022 | 0.2541 | 0.0063 | 0.0614 | 0.0143 |

**Legend.** Beta indicates the unstandardized regression coefficient for the predictor of interest in an age-adjusted model. SE, standard error; CI, confidence interval; Std.Beta, standardized beta coefficient; AdjR2, adjusted coefficient of determination for the full model; p-FDR, Benjamini-Hochberg false discovery rate-adjusted p-value. Green-shaded rows indicate associations significant after FDR correction (p-FDR < 0.05).

**Statistical note.** For each sphingolipid variable, separate age-adjusted ordinary least-squares linear regression models were fitted with BMI, HOMA-IR, diastolic blood pressure (DBP), systolic blood pressure (SBP), C-reactive protein (CRP), HDL cholesterol (HDL-C), or triglycerides (TG) as the predictor of interest. Thus, each sphingolipid-predictor pair was analyzed in an individual model of the form: sphingolipid concentration/value = beta0 + beta1 × predictor + beta2 × age + error. The reported p-value refers to beta1. Standardized beta coefficients were obtained by refitting the same model after z-standardization of the outcome, predictor, and age. False discovery rate correction was applied globally across all 266 predictor-related tests using the Benjamini-Hochberg procedure. No additional adjustment for group status was applied, because the purpose of this analysis was to quantify phenotype-associated clinical/metabolic gradients across the overall study population.
